# Emerging Risk Biomarkers in Cardiovascular Diseases and Disorders

**DOI:** 10.1155/2015/971453

**Published:** 2015-04-08

**Authors:** Ravi Kant Upadhyay

**Affiliations:** Department of Zoology, DDU Gorakhpur University, Gorakhpur 273009, India

## Abstract

Present review article highlights various cardiovascular risk prediction biomarkers by incorporating both traditional risk factors to be used as diagnostic markers and recent technologically generated diagnostic and therapeutic markers. This paper explains traditional biomarkers such as lipid profile, glucose, and hormone level and physiological biomarkers based on measurement of levels of important biomolecules such as serum ferritin, triglyceride to HDLp (high density lipoproteins) ratio, lipophorin-cholesterol ratio, lipid-lipophorin ratio, LDL cholesterol level, HDLp and apolipoprotein levels, lipophorins and LTPs ratio, sphingolipids, Omega-3 Index, and ST2 level. In addition, immunohistochemical, oxidative stress, inflammatory, anatomical, imaging, genetic, and therapeutic biomarkers have been explained in detail with their investigational specifications. Many of these biomarkers, alone or in combination, can play important role in prediction of risks, its types, and status of morbidity. As emerging risks are found to be affiliated with minor and microlevel factors and its diagnosis at an earlier stage could find CVD, hence, there is an urgent need of new more authentic, appropriate, and reliable diagnostic and therapeutic markers to confirm disease well in time to start the clinical aid to the patients. Present review aims to discuss new emerging biomarkers that could facilitate more authentic and fast diagnosis of CVDs, HF (heart failures), and various lipid abnormalities and disorders in the future.

## 1. Introduction

Cardiovascular diseases are increasing day by day due to over utilization of fats or due to genetic reasons. It is a leading cause of morbidity and mortality from infancy to old age. Though conventional risk prediction algorithms are made available on presence of major cardiovascular risk factors identified in diseased population, authentic and accurate biomarkers of CVDs are lacking. It not only delayed clinical diagnosis but also increased risk manifold and resulted in accidental death of patients. Therefore, an early identification and treatment of risk factors are much needed to accelerate disease prevention and morbidity improvement [1]. Numerous risk scores have been developed to predict cardiovascular risk. These scores are based on observations of the relative degree of importance of individual major risk factors. Till the date numerous physiological biomarkers based on serum lipid, glucose and hormone biomarkers serum lipid, glucose and hormone profile have been identified that are associated with increased cardiovascular risks. Some of them are simple traditional biomarkers based on lipid profile and risk factors. More often, levels of plasma, serum, and blood are proved to be best cardiovascular risk biomarkers [2]. These markers display cellular lipid interactions and physiological functions of serum lipid bearing proteins and assist in clinical decision making and authenticated risk type [3]. There are so many established cardiovascular risk markers based on confirmed clinical outcomes related to biomolecules, its structure, and functions. There are new mini- and microlevel clinical factors associated with an elevated prospective risk of developing coronary heart diseases. However, various physical factors if known can work as biophysical markers, but all these are not enough to evaluate the disease and status of emerging risks in patients, hence, other biomarkers to be included in risk analysis. Many of these biomarkers, alone or in combination, can be incorporated into risk prediction models to determine whether their addition increases the model's predictive ability. Moreover, various cardiovascular risk prediction models have been updated by incorporating traditional risk factors and molecular, immunological genetic, imaging, and biophysical factors for more authentic and reliable estimation of cardiovascular risk.

**Figure 1:**
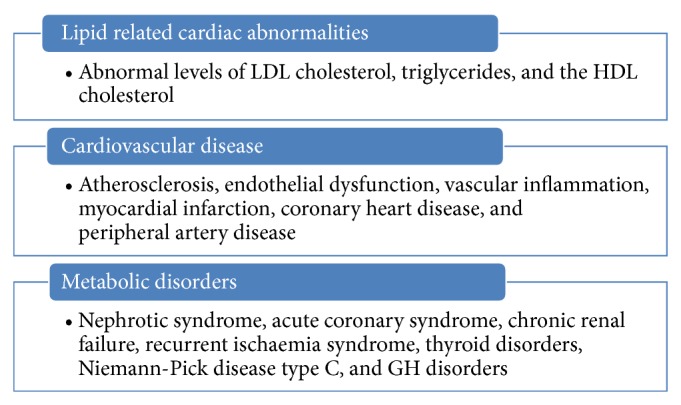
Showing important lipid abnormalities and metabolic disorders related to human cardiovascular disease.

However, to establish risk status measurement of a standard lipid profile, including total cholesterol, LDL (low-density lipoprotein) cholesterol, HDL (high-density lipoproteins) cholesterol, and triglycerides, is recommended from an integral component of approaches to cardiovascular risk prediction. These old markers, such as elevated LDL cholesterol, hypertension, diabetes, and low LDL cholesterol, smoking, and family history can predict premature coronary heart diseases in man. In addition, numerous risk scores have been developed to predict coronary heart disease risks or cardiovascular risk. These scores are based on observations of the relative degree of importance of individual major risk factors. Most important prediction is made by Framingham 10-year risk score which is commonly used to predict cardiovascular event over the next ten years in the primary prevention of disease. Hence, a need persists for diagnosis of CVDs at two stages: first category of patients stratified as low risk (Framingham 10-year risk score >10%) requires less risk identification, modification, and treatment method, but patients stratified as high risk (Framingham 10-year risk score >20%) need intensive risk factor identification. For more appropriate judgment of CVDs, this score incorporates age, total cholesterol, HDL cholesterol, smoking status, systolic blood pressure, and gender [4]. On the basis of scores obtained in patients, these are classified in three groups as scores of <10% low, intermediate 10–20%, and high >20% risk (Table 1). Last category of patients is confirmed as atherosclerotic disease patients and needs early intensive clinical care and factor modification [4] (Figure 1). More specifically, patients with a 10-year risk >20% or with diabetes are considered to be coronary heart disease risk equivalents in terms of the approach to risk modification [5] (Table 1).

Homozygous familial hypercholesterolemia (HoFH) is associated with severe hypercholesterolemia and premature cardiovascular morbidity and mortality. More often, increased cardiovascular risk has also been associated with the presence of obesity, hypertriglyceridemia, chronic kidney disease, and elevated levels of Lp(a) (Table 1). These patients show abnormal levels of LDL cholesterol, triglycerides, and the HDL cholesterol. Another category of patients is associated with hypertriglyceridemia, low LDL cholesterol, and small dense LDL particles. Furthermore, chronic cholelithiasis and primary biliary cirrhosis are associated with hypercholesterolemia due to elevations in systemic levels of rare lipoprotein X, with xanthomata, and hyperviscosity. Moreover, level of saturated fat is inversely associated with atherosclerosis progression in postmenopausal women, whereas polyunsaturated fat (PUFA) and carbohydrates were positively associated [6].

Coronary heart disease is also associated with monocytosis, high diabetics, hypertension, and chronic kidney diseases. The efflux capacity of high-density lipoprotein (HDL) with cultured macrophages associates strongly and negatively with coronary artery disease status, indicating that impaired sterol efflux capacity might be a marker and perhaps mediator of atherosclerotic burden [7] (Table 1). More often, myeloperoxidase may contribute to the generation of dysfunctional HDL with impaired ABCA1 efflux capacity in humans with atherosclerosis. Quantification of chlorotyrosine and oxidized methionine in circulating HDL might be useful indicators of the risk of cardiovascular disease that are independent of HDL cholesterol [7] (Table 1). A fasting profile that incorporates measurements of the total cholesterol, LDL cholesterol, HDL cholesterol, and triglycerides is preferred to simple measurements of total cholesterol alone. However, conventional risk prediction algorithms are prepared based on the presence of major cardiovascular risk factors identified by population studies including hypercholesterolemia, hypertension, diabetes, smoking, low levels of HDL cholesterol, and intermediate-risk patients (10-year risk 10–20%) and may require further investigation to categorize their cardiovascular risk. In addition, assessment of non-HDL cholesterol, total HDl cholesterol and triglyceride, and HDL cholesterol ratios are recommended as secondary measures for risk assessment. Despite the use of risk prediction scores, some patients stratified as low risk experience clinical events.

**Figure 2:**
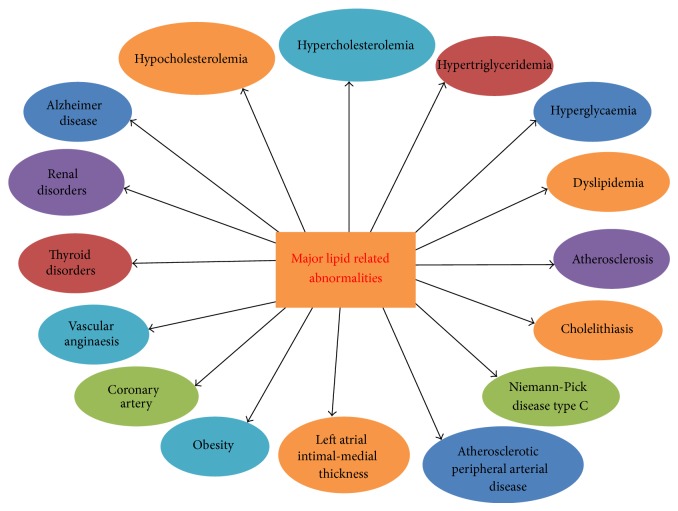
Showing major lipid abnormalities responsible for various cardiovascular disorders found in human.

More often, hyperglycaemia or type 1 diabetes plays a major role in increased incidence of CVD and mortality in individuals. Patients facing type 1 diabetes showed increase of premature mortality, primarily from cardiovascular disease (CVD) [8]. It also indicates that severe lipid disorders may occur in patients with type 1 diabetes, but the occurrence of elevated high-density lipoprotein cholesterol is positively associated with longevity of these patients (Figures 1 and 2). Similarly, nonrenal hypertension by itself is a significant risk factor for CVD but if adequately treated does not appear to mitigate against longevity [8]. In old ages (55–60) measurement of blood pressure and anthropometric and biochemical parameters such as total cholesterol (TC), triglycerides (TG), high-density lipoprotein (HDL), and low density lipoprotein (LDL) assist in finding high risk of CVD, dyslipidemia, and metabolic disorders in patients [8] (Table 1).

Atherosclerosis is the main cause of death in the world through causing ischemic heart disease (IHD) (Figures 1 and 2). It is peripheral arterial disease (PAD), most prevalent, morbid, and mortal disease [9]. It is one of the most common disorders among the elderly, because of depression prevailed in the old age and rates of very high atherosclerosis [10]. Atherosclerosis is characterized by endothelial dysfunction, vascular inflammation, and the buildup of lipids, cholesterol, calcium, and cellular debris within the intima of the walls of large and medium size arteries [11]. Therefore, new emerging biomarkers of myocardial remodeling can develop to identify asymptomatic hypertensive patients at risk for diastolic dysfunction and diastolic heart failure [12]. In addition, lipidemic, hemostasiological, and hemodynamic indicators associated with the risk of cardiovascular death in high- and very high-risk patients. The high levels of von Willebrand factor, D-dimer, ADP-induced platelet aggregation, triglycerides, end-diastolic volume, end-diastolic dimension, and ventricular septal thickness are independent predictors of cardiovascular death in very high-risk patients (Figure 1). These indicators bear out a close relationship between lipid metabolic and hemostatic disturbances and between endothelial dysfunction and intracardiac hemodynamic worsening in these patients [13] (Table 1).

Heart rate (HR) at rest is associated with adverse cardiovascular events [14] mainly it is affected due to high LDL/HDL cholesterol levels [15]. LDL cholesterol plays a pivotal role in the formation and clinical expression of atherosclerotic cardiovascular disease. There is an important connection between HDL and immunity in atherosclerosis [16]. LDL is major transporter of cholesterol in the circulation to peripheral tissues where the cholesterol is used for maintenance of cell membranes. Cholesterol is either reutilized for lipophorin formation or excreted in the bile. LDL cholesterol is directly related to pathogenesis of atherosclerosis and as a therapeutic target to reduce CVD risks. HDL proteins in the systemic circulation consist of core of esterified cholesterol and triglyceride surrounded by surface monolayer of phospholipid and a range of lipoproteins. The HDL also integrates innate and adaptive immunity because during infections or acute conditions high-density lipoproteins cholesterol levels decrease very rapidly and HDL particles increase [16]. Thus, low HDL cholesterol levels predict sever CVD risks, More often, ability of HDL to influence cholesterol availability in lipid rafts in immune cells results in the modulation of Toll-like receptors, MHC-II complex, and B and T cell receptors, while specific molecules shuttled by HDL such as sphingosine-1-phosphate (S1P) contribute to immune cells trafficking [16]. It has also been tried to correlate lipid abnormalities with hypertension, diabetes, and cardiovascular diseases. Moreover, various cardiovascular risk prediction models have been updated by incorporating traditional risk factors and molecular, immunological genetic, imaging, and biophysical factors for more authentic and reliable estimation of cardiovascular risk. Many of these biomarkers, alone or in combination, have been incorporated into risk prediction models to determine whether their addition increases the model's predictive ability. Similarly, myocardial infarction can be assessed by using circulating microRNAs level in patients [17] and Omega-3 Index as a risk factor for cardiovascular diseases [18] (Tables 1 and 2).

**Figure 3:**
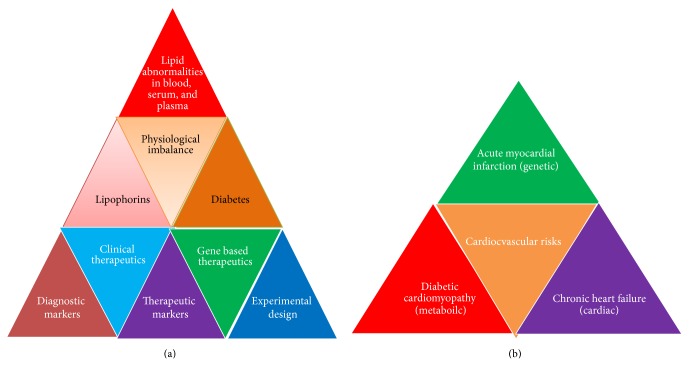
(a) and (b) showing interrelationship of lipid abnormalities and associating risk factors with diagnostic and therapeutic measures.

Despite recent treatment advances and clinical methods available, there is an increase in cardiovascular diseases (CVD) mortality cases every year. There are terrible reports on cases of hypercholesterolemia management as the percentage of individuals with LDLc plasma concentration has been alarmingly increased and cardiovascular risk sets are very high. Therefore, both diagnostic and additional therapeutic strategies are highly needed to evaluate CVDs and other lipid abnormalities. Hence, more prompt and continuous efforts are needed to develop new biomarkers for achieving high diagnostic accuracy to predict CVD risks. In addition, good preventive therapies are also needed to stop rising death toll due to CVDs. Thus, risk stratification and assessment of cardiovascular risks in cardiac patients are important areas of research in clinical biology [19]. It warrants further investigations to determine ultramodern emerging risk biomarkers for CVD for more appropriate risk assessment [20] (Figure 1). These biomarkers will not only improve clinical decision making but also help in exploring CVD risk types in man. Therefore, after seeing the severity and massive increase in CF cases both diagnosis and CVD therapeutics will be highly needful. This paper addresses the applications of various types of emerging biomarkers for prediction of CVDs in patients and therapeutic markers of very high clinical importance (Figures 2 and 3).

## 2. Brain Function and Lipids

Cholesterol is an essential component of both the peripheral and central nervous systems of mammals. Brain cholesterol is synthesized* in situ* by astrocytes and oligodendrocytes and is almost completely isolated from other pools of cholesterol in the body, but a small fraction can be taken up from the circulation as 27-hydroxycholesterol or via SR-BI. Glial cells synthesize native HDL-like particles, which are remodeled by enzymes and lipid transfer proteins, presumably as it occurs in plasma. The major apolipoprotein constituent of HDL in the CNS is apolipoprotein E, which is produced by astrocytes and microglia. Apolipoprotein A-I, the major protein component of plasma HDL, is not synthesized in the CNS but can enter and become a component of CNS lipoproteins. Low HDLc levels have been shown to be associated with cognitive impairment and various neurodegenerative diseases. On the contrary, no clear association with brain disorders has been shown in genetic HDL defects, with the exception of Tangier disease. Mutations in a wide variety of lipid handling genes can result in human diseases, often with a neuronal phenotype caused by dysfunctional intracellular lipid trafficking. Transient global cerebral ischemia, one of the consequences of cardiac arrest and cardiovascular surgery, usually leads to delayed death of hippocampal cornu ammonis 1 (CA1) neurons and cognitive deficits. Currently, there are no effective preventions or treatments for this condition. Omega-3 polyunsaturated fatty acids (*ω*-3 PUFAs) have been shown to have therapeutic potential in a variety of neurological disorders [21]. N-acylethanolamines (NAEs) having role in lipid signaling in brain and highlighting multipotential actions on neuronal cell death and neuroinflammatory pathways can become good biomarker for lipid based disorders in several groups of patients [22] (Figure 2).

## 3. Lipid Abnormalities and Cardiovascular Risks

Cardiovascular diseases are prevalent in human population and most of them are related to diet but genetic lipid abnormalities such as hypercholesterolemia, hypertriglyceridemia, HDL metabolism disorders, and combined hyperlipidemias are more severe. In addition, diseases like dyslipidemia/hyperlipidemia, atherosclerosis, familial hypercholesterolemia, hypertriglyceridemia, and diabetes are also prevalent in pediatric groups. Hypertriglyceridemia is commonly found in patients with chronic renal failure which is largely due to decreased activity of lipoprotein and hepatic lipase and selective enrichment with apolipoprotein C-II. Each of them contributes to reduced hydrolysis of triglyceride-containing particles (Table 1). Triglyceride abnormalities are more commonly encountered in patients treated with peritoneal dialysis, which may reflect the presence of glucose in dialysate. Elevated levels of Lp(a) and low HDL cholesterol are also encountered and contribute to the accelerated rate of cardiovascular disease. Cardiac implications are also related to hypoglycaemia in patients with diabetes [23]. Moreover, both type 1 and type 2 diabetes are considered to be high-risk conditions and have stringent cholesterol targets. Similarly, common cholesterol disorders, mainly dyslipidemia, were also found to be specific to the pediatric diabetes population [1] (Figure 3). Altered serum lipid level is the most important risk factor for coronary artery disease (CAD) (Table 1).

Lipid droplets (LDs) are simple, inert lipid micelles. These also hold proteins and behave as organelles in a myriad of cellular processes. These are heterogeneous in nature with different sizes and compositions of phospholipids, neutral lipids, and proteins. These lipid droplets create ruckus in streaming blood and cause blood vascular problems. All lipids related abnormalities are responsible for cardiovascular mortality, fatal myocardial infarction, and brain stroke. More often, few proteins such as fat-specific protein (Fsp27), fat storage-inducing transmembrane (FIT) proteins, and seipin and ADP-ribosylation factor 1-coat protein complex I (Arf-COPI) are involved in the regulation of LD formation, expansion, and morphology [24]. Nephrotic syndrome is associated with elevated levels of LDL cholesterol, triglycerides, and Lp(a). It occurs as a result of increased hepatic apolipoprotein B synthesis, due to reduced oncotic pressure and reduced catabolism of LDL and lipoprotein lipase activity. Similarly, hypothyroidism is associated with elevated levels of LDL cholesterol and triglyceride, either in isolation or in combination. Reductions in LDL receptor expression and activity, biliary cholesterol excretion, and lipoprotein lipase activity underlie the lipid abnormalities. Hyperthyroidism is associated with excessive activity of each of these factors and is, therefore, typically associated with low levels of LDL cholesterol and triglycerides. Abdominal obesity is also an important visible sign and display of elevated levels of VLDL (very high-density lipoprotein) and of triglycerides and low levels of HDL cholesterol. For fighting obesity, calorie burning by slow and regular exercise results in weight loss. Overweight and obesity are complex health problems that mostly affect adults. There are many health conditions associated with overweight and obesity including hypertension, coronary heart disease, and type 2 diabetes. Obesity can be cut down by making dietary modification and therapeutic lifestyle changes (TLC). TLC is an effective lifestyle therapy targeting low-density lipoprotein cholesterol (LDL), a risk factor for coronary heart disease. Along with lowering LDL, TLC also improves risk factors associated with the metabolic syndrome and diabetes, including blood pressure, high-density lipoprotein cholesterol (HDL), serum triglycerides, blood glucose, and weight status (Figures 2 and 3). There are so many associating factors which can assist in emerging risks for cardiovascular diseases [25] (Table 1).

**Figure 4:**
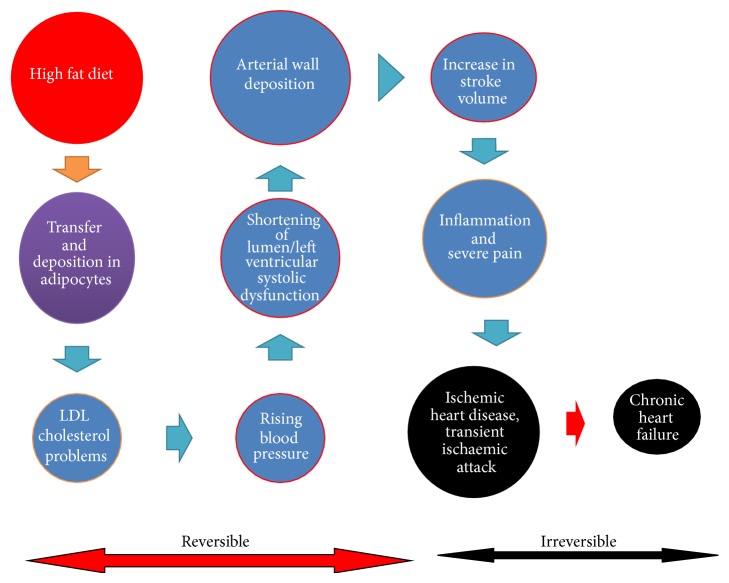
Showing successive progression of transient ischemic attack and chronic heart failure in man.

Cardiovascular risks such as defects in angiogenesis/vasculogenesis or vessel repair are major complications of coronary artery disease (CAD) which are mostly seen in aged people. Similarly, CVD risks have also increased in women during pregnancy which is an important issue for management of their cardiovascular health [26]. Cannabis-associated myocardial infarction is observed in young man with normal coronary arteries [27]. In developed countries, there is a large population that shows an increased frequency of atherosclerosis (ATH) mainly systemic lupus erythematosus (SLE). There are paradoxical reports on CAD in South Asian Ethnicity and Cardiovascular Risks [28], but most of atherosclerotic risk factors and atherosclerotic postoperative events are associated with low inflammation in abdominal aortic aneurysms [29]. Similarly, severity of subclinical cardiovascular disease among those with nonalcoholic fatty liver has been alarmingly increased [30] with a significant acute myocardial infarction. Hence, there is an urgent need of potential novel cardiac biomarkers for prediction of acute myocardial infarction [31]. More often, highly sensitive cardiac biomarkers are needed to explore cardiovascular risks, morbidity, and mortality in elderly men [32]. These are also required to predict acute coronary syndrome [33] and for prediction or finding level and type of risk and its assessment in postinfarction heart failure [34] and vasculogenic erectile dysfunction [35]. However, by predicting value of serum soluble ST2 and interleukin-33 are used for risk stratification and prognosis in patients with acute myocardial infarction [36] while circulating biomarkers such as different factors, proteins, ions, stimulators of heart muscles, or deactivators can predict heart failure [37] (Figure 4). Few important body activities such as inflammation, obesity, thrombosis, and autoantibodies also display procardiovascular risks [38] and act as emerging biomarkers [39] (Table 1). Similarly, sedentary behaviour was also proved as an emerging risk factor for cardiometabolic diseases in children and youth [40].

## 4. Hypertension and Cardiovascular Risks

Hypertension and dyslipidemia are the most prevalent cardiovascular risk factors, with approximately 350 million people having these concomitant conditions throughout globe. Hypercholesterolemia in midlife is related to an increased risk of Alzheimer's disease (AD) in later life. Another possible mechanism, hypercholesterolemia, may be associated with hypoperfusion through the progression of atherosclerosis [41]. Similarly, dyslipidemias is characterized mainly by elevated levels of total cholesterol and low-density lipoproteins in cardiovascular patients. Higher triglyceride levels and lower high-density lipoproteins are encountered 2 and 1.5 times more frequently, respectively. Age-related changes and metabolic hepatic disorders associated with alcohol abuse and consequences of prior infectious diseases play an important role in the pathogenesis of dyslipidemias in patients over 40–45 years of age [42]. Vascular dementia is caused by stroke that occurs due to hypertension. More often for evaluation of lipid related disorders demographic, diagnostic, and medication-related factors are associated with BP (blood pressure) and LDLc goal attainment in patients with concomitant hypertension and dyslipidemia stratified by body mass index BMI. Many more variations were found in therapeutic care in patients with concomitant hypertension and dyslipidemia across different BMI groups. Further, the presence of high body mass index (BMI) has a negative effect on the achievement of blood pressure (BP) and low-density lipoprotein cholesterol (LDLc) targets. Hence, string markers may be needed for improving these disparities [43] (Figure 3). In addition, concentrations of total cholesterol, LDL cholesterol, HDL cholesterol, triglycerides, apolipoprotein A-I, apolipoprotein B, and lipoprotein in serum of patients are biomarkers of primary hypertension and with hyperhomocysteinemia [44] (Table 1).

## 5. Diabetes and Cardiovascular Risks

Cardiovascular disease is more prevalent in type 1 and type 2 diabetes and continues to be the leading cause of death among adults with diabetes. Diabetes coexists as a more severe risk factor with other associating risk factors, in particular with dyslipidemia. It increases cardiovascular risks due to increased levels of triglycerides, low levels of high-density lipoprotein cholesterol, and postprandial lipidemia. Dyslipidemia is mostly observed in patients with type 2 diabetes or metabolic syndrome [49]. In addition, atherosclerotic vascular disease (AVDs) shows obstruction in streaming blood functions due to arterial thickness and high blood pressure. In AVDs, lipid metabolism plays a central role. However, measurement of arterial stiffness provides assessment of endothelial dysfunction and diagnosis of atherosclerotic burden in patients with MetS [50]. Therefore, total serum *γ*-glutamyl transferase activity (GGT) represents the impact of metabolic disease on vascular injury and atherosclerosis [165]. It acts as important biomarkers of arteriosclerosis in the Multiethnic Study of Atherosclerosis (MESA) [45]. Similarly, *β*-trace protein from GFR marker is also used as cardiovascular risk predictor [51]. There are other markers such as occurrence of a fatty kidney and ectopic lipid in obesity-related renal disease that is also associated with CVDs and are emerging risk predictors [166] (Figure 3). Lipid abnormalities are also analyzed by MS [167] and MALDI mass spectrometry imaging and lipidomics for clinical diagnosis [52]. Similarly, human serum proteome analysis has been emerged as a new source of markers for knowing metabolic disorders [53] (Table 1). Similarly, defects in angiogenesis/vasculogenesis or vessel repair are major complications of coronary artery disease (CAD).

Among all different types of cardiovascular diseases, atherosclerosis is the main cause of death in the world through causing ischemic heart disease (IHD). This is also associated with endothelial dysfunction, monocyte accumulation, endothelial apoptosis, and thrombus formation which affect cardiovascular functions. Atherosclerosis is partially related to altered serum lipid level, and cholesterol deposition arterial wall that is one of the most important risk factors for coronary artery disease (CAD). In atherosclerosis, the LDL is an important biomarker while high-density lipoproteins are associated with atherosclerotic kidney disease [54]. There occurs a strong inverse association between low levels of high-density lipoprotein cholesterol (HDLc) and increased risk of IHD. On the other hand, a plasma level of HDLc has a strong hereditary basis. It is a genetic defect that reduces the level of HDLc and has a role of ATP binding cassette protein (ABCA1), apolipoprotein A1 (ApoAI), and lecithin cholesteryl acyltransferase. Lipoprotein lipase (LPL) and angiopoietin-like 3 proteins (ANGPTL3) are associated with low HDLc. Other potentially important candidates involved in low HDLc syndromes are metabolic disorders including sphingomyelin phosphodiesterase 1 and glucocerebrosidase. However, molecular variations occur in many genes such as ABCAI and ApoAI, TRIB1 and apolipoprotein E (ApoE), lipoprotein lipase (LPL), WW domain-containing oxidoreductase (WWOX), hepatic lipase (HL), lecithin cholesteryl acyltransferase (LCAT), and some linkage analysis have been associated with reduced HDL status. Low HDLc syndrome has a strong genetic basis and is correlated with an increased risk of CAD [55] (Figure 3). More often, blood genomic profiling is applied to atherosclerosis and transient, ischemic stroke patients to predict the risk [46, 47]. Besides, atherosclerosis [168] cerebral revascularization in the endovascular tissue [169] is important clinical issue in present time [168] (Table 1). Both alcohol and cardiovascular risks are proved double-edged sword which cause high fatality in man [170].

Disturbances in lipid metabolism which occur during hypothyroidism lead to the formation of gallstones. Moreover, both gallstone and bile duct stone in hypothyroidism are more prominent indicator of lipid related disorders [56]. There seems an association between thyroid disorders and the presence of bile duct stones [56]. Similarly, hepatic inflammation due to common carotid intima-media thickness [57], recurrent ischaemia syndrome [171], coronary artery disease [172], diabetes and stroke [58], and hypertension is important cardiovascular pathogenesis which needs high grade diagnosis and appropriate therapeutic targets [173, 174]. There are so many methods known which can establish association of inflammatory markers with cerebral vasoreactivity and carotid atherosclerosis in transient ischemic attack [175]. Similarly, *α*4*β*7 Integrin (LPAM-1) is also found to be related to atherosclerosis progression [48]. Serum metabolic profiling [176] and serum total p-cresyl sulfate level are measured to predict CVD risks mainly in angina patients [177] (Figure 4). There are some accessory factors such as alcohol, smoking, obesity, arterial thickness, BMI, and respiration rate which increase cardiovascular severity many times [178]. Patients with type 2 diabetes mellitus (T2DM) frequently exhibit macrovascular complications of atherosclerotic cardiovascular (CV) disease [179]. Low levels of HDL cholesterol (HDL-C) independently contribute to CV risk and show increased risk of coronary heart disease but high-density lipoproteins (HDL) are found protective against atherosclerosis. However, therapeutic targets can be achieved by increasing concentrations of HDLc [180]. Atrial fibrillation (AF) is a very common tachyarrhythmia and is becoming increasingly prevalent venous thrombosis. Besides stroke, altered cerebral blood flow in AF and cerebral microbleeds from anticoagulation may enhance the risk of dementia [59]. More often, levels of microRNAs play emerging roles in adipogenesis and obesity [60] while P255Galectin-3 was found to be a new promising cardiac biomarker in sports endurance [181]. White blood cell (WBC) count has been associated with diabetic risk, but it needs correlation of independent risk factors. Both WBC count and glucose metabolism can predict diabetes in middle-aged and elderly people. In addition, loss of weight, smoking cessation, lipid-modifying therapies, and control of postprandial plasma glucose and HbA1c may ameliorate the chronic low-grade inflammation [182] (Table 1).

## 6. Biomarkers of Coronary Artery Disease

Clinical reports presented massive prevalence of coronary artery disease (CAD) in human youths and aged females and males, due to missed or delayed diagnosis. Major cardiovascular disorders are being recognized earlier in life in new ages because of genetic abnormalities. From studies, it is also made clear that CVDs risk factor varies from person to person but pathogenesis is realized in similar manner. There are many known markers available to predict CAD or CVDs including acute coronary syndromes [183, 184] but seeing severity and rising morbidities in all age groups upgraded biomarkers are to be needed for an earlier assessment of CAD (coronary artery disease) patients. There are many emerging biomarkers for coronary artery diseases which can diagnose the CVD. These important biomarkers are inflammatory mediators [185], spectral analysis of electrocardiography [186] vitamin D status and cardiometabolic [64], aortic wave reflection and pulse pressure amplification, and coronary CT angiography [62]. These are more appropriate risk markers which can evaluate status of coronary artery diseases in old ages [187] (Figure 4). Similarly, automated quantification of epicardial adipose tissue (EAT) and manual assessment of coronary CT angiography can establish correlation with coronary artery disease [62] and work as clinically important biomarkers in acute coronary syndrome [63] (Table 1).

Peripheral artery disease (PAD) is a marker disease for generalized atherosclerosis and represents one of the world's major causes of morbidity and mortality [188]. Moreover, peripheral venous blood flow rate also functions as important coronary sinus biomarkers [189]. Similarly, level of proinflammatory markers (hs-CRP, IL-6, and ICAM-1) and pregnancy associated plasma protein-A (PAPP-A) in serum also clearly display myocardial infarction (MI). No doubt, hs-CRP is the potential marker to discriminate cases of UA from controls while PAPP-A is the reliable marker which can discriminate the cases of MI from UA and controls [61]. Similarly, *β*2-microglobulin, cystatin C, and creatinine are important risk markers of symptomatic peripheral artery diseases [190]. P194Serum uric acid is independent predictor of decreased number of circulating proangiogenic progenitor cells in asymptomatic coronary artery disease patients [191], while P733regulatory B cells from patients with coronary artery disease display numerical and functional alterations, a novel immune defect in atherosclerosis [192]. Similarly, phospholipase A2 enzymes can predict ischemic events after acute coronary syndromes [193], while plasminogen-plasmin level is considered as an important biomarker of fibrinolysis [194]. Similarly, atrial fibrosis is a* risk* stratifier for atrial fibrillation [195], while mitogen-activated protein kinases [196] and intercellular adhesion molecule1 gene polymorphism are good markers of coronary heart diseases [197] mainly for atherosclerosis [196]. Similarly, aortic vascular functions are also used for assessment of cardiovascular risks [198] and provide stratification in asymptomatic severe aortic stenosis [199]. Besides this, traditional cardiac risk factors are also used as markers for disease diagnosis which are too old to clear out major reasons of cardiovascular problems in different groups of man. More often, several nontraditional biomarkers, including proinflammatory high-density lipoprotein (piHDL) and leptin, have been individually associated with subclinical ATH in SLE. In addition few important biomarkers are combined into a risk profile to predict increased risk of cardiovascular disease in patients with SLE [174] (Figure 3). However, lipid related markers [200] or emerging lipoprotein risk factors are used for prediction and assessment of cardiovascular risks in all age groups [20] (Tables 1 and 2).

## 7. Serum Biomarkers

### 7.1. Triglyceride to HDL Cholesterol

Triglyceride to high-density lipoprotein cholesterol ratio, total cholesterol to high-density lipoprotein cholesterol ratio, and low ankle brachial index in an elderly population can predict risks of cardiovascular diseases [80]. The associations of triglyceride (TG) to high-density lipoprotein cholesterol ratio (HDLc) and total cholesterol (TC) to HDLc ratio and low ankle brachial index (ABI) are important biomarkers which predict CVDs [80]. Although triglyceride levels are obtained in most standard lipid panels, but it can be used in CVD risk prediction by using some other lipid profile related markers. Increasing evidence suggests that both fasting and nonfasting triglyceride levels predict prospective cardiovascular risk. Therefore, triglyceride HDL cholesterol ratio has become increasingly important, providing mixed dyslipidemic patterns in the setting of obesity and the metabolic syndrome [80]. A triglyceride: HDL cholesterol ratio >3.5 appears to be associated with increased cardiovascular risk. Similarly, a significant increase in total cholesterol, high-density lipoprotein cholesterol, low-density lipoprotein cholesterol (LDL-C), apolipoprotein B (ApoB), and lipoprotein (a) (Lp(a)) and a significant decrease in apolipoprotein A1 (ApoA1), ApoA1/ApoB ratio, and PON1 activity/HDLc ratio are used as important biochemical markers [81]. Total cholesterol, LDL-C, ApoB, Lp(a), and ApoA1/ApoB ratios are also considered as good biomarkers for prediction of CVDs in man [201] (Table 2). More often, a high plasma triglyceride concentration, low HDL cholesterol concentration, and increased concentration of small dense LDL-cholesterol particles are an indication of diabetic dyslipidemia. The lipid changes associated with diabetes mellitus are attributed to increased free fatty acid flux secondary to insulin resistance. Insulin resistance and type 2 diabetes are associated with a clustering of interrelated plasma lipid and lipoprotein abnormalities, which include reduced HDL cholesterol, a predominance of small dense LDL particles, and elevated triglyceride levels. In fact patients with higher level of LDL, triglycerides and total cholesterol (hyperlipidemia), affected with type 2 diabetes mellitus (T2DM) remain at higher risk of coronary artery disease [201]. Total cholesterol, LDL-C, ApoB, Lp(a), and ApoA1/ApoB ratios are also considered as good biomarkers for prediction of CVDs in man [201] (Table 2).

### 7.2. Lipid-Lipoprotein Ratio

Lipid-lipoprotein ratio can predict severe cardiovascular risks in both early age and old age patients. Moreover, it was also established in population studies that increasing levels of oxLDL are associated with greater cardiovascular risk. Therefore, measurement of antibodies against oxidized forms of LDL (oxLDL) may play a role in risk stratification. But it remains unclear whether oxLDL provides an incremental or greater ability to predict risk compared with measuring LDL cholesterol. More often, LDL cholesterol does not account for the entire cohort of atherogenic lipid particles within the systemic circulation. Therefore, calculation of non-HDL cholesterol has become increasingly popular because it represents the full complement of atherogenic lipids. Similarly, lipoprotein particle size and number, as determined by nuclear magnetic resonance, are used as an alternative approach to evaluation of lipids abnormalities related to cardiovascular risk. However, both low levels of LDL cholesterol and the number of small LDL particles increased or decreased are an indicator of cardiovascular risks. These small, dense LDL particles can be used to establish relationship with the metabolic syndrome and abdominal obesity in adults. In a similar fashion, raising the concentration of small HDL particles appeared to predict the clinical benefit of drugs on clinical events in patients with established coronary heart disease. Hence, there is need to increase standardization, validation, and authentication of newly emerging biomarkers in cardiovascular risk prediction and monitoring of therapeutic response (Table 2).

Besides measurement of LDL and HDL cholesterol, quantification of lipid particles, apolipoprotein B (ApoB), and apolipoprotein (AI) provides a direct measure of atherogenic and therapeutic role in human population. More often, number of lipid particles may directly influence the accumulation of lipid within the artery wall. However, levels of ApoB and ApoAI or ratio of ApoB : ApoAI are strongest predictor of myocardial infarction and statin is a predictor of slowing progression of coronary atherosclerosis. In addition, assessment of ApoB and ApoAI may provide incremental risk prediction when used in place of LDL and HDL cholesterol, respectively. More specifically, using an apolipoprotein-based approach to risk prediction will become a cost-effective method.

Apolipoproteins are very heterogeneous protein family, implicated in plasma lipoprotein structural stabilization, lipid metabolism, inflammation, or immunity. Hence, apolipoprotein composition and structure may contribute to elucidating lipoprotein roles in atherogenesis. It may also assist in developing new therapeutic strategies for the treatment of lipoprotein-associated disorders. Apolipoprotein E effects free high-density lipoproteins and cholesterol metabolism. Therefore, characterizing the apolipoprotein component of plasma VLDL, LDL, and HDL fractions from patients can predict the vulnerability of disease in atherosclerotic patients. The higher levels of AP SAA found in patients elucidate the role of LDL as AP SAA carrier into the subendothelial space of artery wall, where AP SAA accumulates and may exert noxious effects [202]. Similarly, lipoprotein-associated phospholipase A2 (Lp-PLA2) circulates on LDL particles and is thought to be involved in the promotion of inflammation, as a result of the release of arachidonic acid metabolites. Moreover, elevated levels of Lp-PLA2 are found to be associated with an increased risk of cardiovascular events in patients which later on evoke cardiovascular disease. Lp-PLA2 levels have also been demonstrated to be reduced by statins, suggesting that they may potentially be a target for therapies in addition to being used as a marker of cardiovascular risk [82] (Table 2).

### 7.3. Lipophorins as Biomarkers

Different types of lipids, that is, diacylglycerol, phospholipids, cholesterol, and hydrocarbons, are transported between the different systems of the body by lipophorins. A lipid transfer protein from human plasma has been characterized which show microheterogeneity [202]. A major function attributed to high-density lipoproteins (HDL) is the ability to remove excess cholesterol from extra hepatic cells, the first step of reverse cholesterol transport pathway (1). HDLp can promote the net efflux of cellular cholesterol. The removal of cellular cholesterol is led by lipoprotein particles solely depending on the size and lipid composition. In removal of extra cholesterol, cell membranes also play a significant role that depends on desorption of cholesterol from the membrane role into aqueous compartments surrounding the cell where it becomes accessible for incorporation into appropriate acceptors. The driving force for the removal of cholesterol from the plasma membrane is the establishment of a concentration gradient from the membrane to the acceptor particle. By this mechanism, HDLp functions in a passive capacity as a cholesterol acceptor for the plasma membrane cholesterol [203]. The interaction of high-density lipoproteins (HDL) with the HDL receptor stimulates the translocation of cholesterol from intracellular pools to the plasma membrane where the cholesterol becomes available for removal of appropriate receptor. Removal of intracellular cholesterol results in increased sterol synthesis, increased expression of LDL receptors, and decreased cholesterol esterification [204]. Cholesterol removal is also depending on efflux of cellular cholesterol and concentration of ACAT (acyl-coenzyme A: cholesterol acyltransferase) [83] and HDL protein [84]. Both can work as important markers for confirming concentration and condition of intracellular cholesterol associating with some CVD. However, entire mechanism of lipoprotein cholesterol mixing with cellular cholesterol and subsequent ACAT activation is still unknown. Moreover, lipid carrier protein was made after the confirmation of cytosolic factors present in the cells which were required for microsomal synthesis of cholesterol or which could accelerate the transfer or exchange of phospholipids between membrane preparations. Thus, most of the lipid exchange, or binding of specific phospholipids, unesterified fatty acids, and/or less polar lipids, occurs with the help of lipophorins (Figure 4). Physiologically FABP (fatty acid binding proteins) also binds with cholesterol and enhances cholesterol transfer between membranes [205, 206] and is found in liver and intestinal cytosol (Table 2). If measured by using ELISA, these could also appropriately assist in establishment of CVDs in man.

In mammals, different M-FABPs are found in different tissues [85] and cells [207]. Its main function is to transport fatty acids through the cytosol to mitochondria for subsequent beta oxidation. It also moves to the muscle cells inside which lipid oxidation takes place. Muscle cells contain high concentration of FABP that also favors beta-oxidation, rate of lipid utilization, and intracellular content of FABP. In rat heart 5% of the total cytosolic proteins are FABP. This concentration of FABP also favors beta-oxidation, rate of lipid utilization, and intracellular content of FABP. Biosynthesis of FABPs starts according to its presence in intracellular pool, and its expression takes place by sterol regulatory DNA elements and their binding with proteins [208]. These DNA regulatory elements also control lipid homeostasis by intracellular lipid transport [86] which could also emerge as CVD risk markers. There is a possibility that in obese patients and pregnant women FABP concentration may increase many folds which also led to a significant increase in amount of its mRNA. This is also true that FABPs are sole carrier molecules whose activity is controlled by gene expression after attaining a specific mRNA level. After rising up to a maximal concentration, it starts decreasing rapidly to a very low level. This represents resting state and no further rapid utilization of fatty acids may occur [87]. Interestingly, expression of different FABP [209] reflects the ability to functional utilization of energy in body muscles [210]. A similar state of FABP, its mRNA, and lipid utilization may also predict the state of CVDs in human if fully explored (Table 1).

Fatty acid binding proteins carry fatty acid molecules to the cell [211] which are found in the cytosol of a variety of mammalian tissues [212] and show high binding affinities [213] with other molecules also. The interaction between fatty acids and FABP is thought to be involved in the regulation of intracellular FFA levels [214]. X-ray crystallographic studies have demonstrated that FA binding involves highly specific interactions between the FA and amino acid residues at a site that lies buried within the FABP proteins. Thus, binding of long chain fatty acids occurs at a site that appears to be designed specifically to a nonspecific site on the protein surface. Cholesterol can be taken up from lipoproteins in the circulation by mechanism involving desorption transfer of cholesterol from the lipoprotein to the plasma membrane bilayer or by receptor mediated uptake [208]. Low density lipoprotein and its receptor make the transport of cholesterol after proper binding. Besides extracellular transport intracellular transport is also important for cholesterol association with proteins, and it also enhances phospholipid binding and aggregation in annexins by their core domain [215]. As an essential constituent cells can reutilize protein bound cholesterol for signal transduction, adhesion, and motility [216]. Inside the cell free cholesterol or extra cholesterol is esterified by an enzyme, that is, acyl-coenzyme A: cholesterol acyltransferase [217–219], which also helps in cholesterol transport in a cyclic way constitutively between the cell interior and the plasma membrane [220]. No doubt, cholesterol and lipoprotein interactions in intracellular pools and on membrane surface may contribute a lot in assessment of cholesterol-associated pathogenesis in CVD patients [221] (Table 2).

### 7.4. LDL Cholesterol Levels

Raised low-density lipoprotein cholesterol (LDLc) plasma concentration is a major risk factor for atherosclerotic cardiovascular disease. Moreover, a sharp rise in LDLc plasma concentration indicates high cardiovascular risk and needs earlier clinical care [222]. Similarly, autosomal dominant hypercholesterolemia (ADH) is characterized by markedly elevated level of LDL cholesterol that leads to premature morbidity and mortality from atherosclerotic cardiovascular disease (ASCVD). Similarly, familial combined hyperlipidemia (FCHL) is characterized by variable elevations of total cholesterol, triglycerides, or LDL cholesterol and a high risk of premature ASCVD [223] (Table 2). There is strong relationship between plasma levels of small dense low-density lipoprotein cholesterol (sdLDLc) and risk for incident coronary heart disease (CHD) [224]. Low-density lipoprotein receptor-related protein-1 (LRP1) is a large endocytic and signaling receptor that is widely expressed in the liver, where it plays an important role in regulating the plasma levels of blood coagulation factor VIII by mediating its uptake and subsequent degradation. Blood coagulation factor VIII is a key plasma protein that is deficient in hemophilia A and circulates in complex with von Willebrand factor. In the vasculature, LRP1 also plays protective role from the development of aneurysms. Mice in which the lrp1 gene is selectively deleted in vascular smooth muscle cells develop a phenotype similar to the progression of aneurysm formation in human patient, revealing that these mice are ideal for investigating molecular mechanisms associated with aneurysm formation. LRP1 protects against elastin fiber fragmentation by reducing excess protease activity in the vessel wall [225]. These proteases include high-temperature requirement factor A1, matrix metalloproteinase 2, matrix metalloproteinase-9, and membrane associated type 1 matrix metalloproteinase. In addition, LRP1 regulates matrix deposition, in part, by modulating levels of connective tissue growth factor. However, pathway LRP1 has important role in thrombosis that may assist in effective intervention in patients [225]. Similarly, small dense LDL is considered as an emerging risk factor for cardiovascular disease [226] (Table 2).

LDL cholesterol is not only cycled to the cell surface but also transported to the ER [227] where cholesterol may become esterified by the enzyme (acyl-coenzyme A: cholesterol acyltransferase (ACAT)) [228]. This esterification is activated as a means to detoxify excess free cholesterol and the esters are deposited in cytosolic lipid droplets. Two pathways have been implicated in the delivery of endocytosed LDL cholesterol to the ER (endoplasmic reticulum). First pathway involves a retrograde route through the Golgi complex while another pathway is cytochalasin D inhibitable. This retrograde connection from the endosome to the ER bypasses the Golgi and could be protein mediated. Intracellular cholesterol trafficking could occur by at least three potential mechanisms: (a) spontaneous diffusion, (b) protein carrier mediated diffusion, and (c) vesicular transport. The Golgi complex has been proposed to play a role in the relocation of cholesterol from lysosomes to other cellular membranes [228]. Uptake of low-density lipoprotein increased Golgi complex concentration, with the greatest increase occurring in the TGN (trans-Golgi network). It has been shown that cholesterol moves bidirectionally between the plasma membrane and intracellular compartments including the ER and lysosomes [229].

In intact cells, a carrier protein could facilitate cholesterol transport by increasing the rates of cholesterol desorption from the lipid bilayer and/or stabilizing the cholesterol after it leaves the bilayer. One candidate protein involves in transport of the cholesterol from small intestine to the blood and various parts of the body after cholesterol absorption from the diet. As an alternate to a specific carrier cholesterol transport to the ERC (endocytic recycling compartment) could be facilitated by many cytoplasmic proteins with hydrophobic binding pockets that provide a platform that shields cholesterol from the aqueous cytoplasm. A similar molar stoichiometry of 1 : 1 has been obtained in case of SCP2 (sterol carrier protein-2) mediated transfer of cholesterol and its accumulation by mitochondrion which is unable to further metabolize the sterol. Unesterified fatty acids are very important metabolic fuels and as key intermediate metabolic products of lipid metabolism. These also perform very important vital role in signal transduction and modulation of ionic channel opening. Cells also require a constant influx of free fatty acids for lipid resynthesis and metabolic energy. Fatty acids also act as second messengers. Other important nutrients to the cells are transported with other carrier proteins and do not require special flux or quick raft. But certainly for the protein bound absorption and transport of cholesterol molecules pH may also play an important role that may work as a special marker about streaming cholesterol.

### 7.5. HDLp and Apolipoproteins Level

Both HDLp and its components also indicate emerging risks of cardiovascular diseases vis a vis apolipoprotein level. Measurements of apolipoproteins B, apolipoproteins A-I, and lipid particle size also work as cumulative indicator and correlate with cardiovascular risks. In particular, the paraoxonase (PON) family of enzymes, which circulates predominantly on HDL particles, plays a role in the antioxidant and anti-inflammatory activities of HDL. Hence, low levels of PON activity are associated with elevated systemic levels of markers of oxidative stress and with a greater likelihood of clinical events. Over all examination of lipoproteins in patient's serum also elucidate cardio-metabolic risk stratification [88]. Recently, the cholesterol efflux capacity from macrophages was also proven to be an excellent metric of HDL functionality. It has a strong inverse relationship with the risk of angiographically documented coronary artery disease, independent of the HDL and apolipoprotein A1 levels, although it may not actually predict the prospective risk for cardiovascular events. Thus, improving the quality of HDL may represent a better therapeutic target than simply raising the HDL level and assessment of HDL function may prove informative in refining our understanding of HDL-mediated atheroprotection [230]. Thus, measurement of standard lipid profile, including total cholesterol, LDL cholesterol, HDL cholesterol, and triglycerides is recommended to form an integral component of approaches to cardiovascular risk prediction. Moreover, patients with established atherosclerotic disease are considered to be at high risk and should undergo intensive treatment and risk factor modification. Therefore, modification of high LDL and low level of high-density lipoprotein (HDL) in cardiovascular atheroprotection is highly needful (Table 2).

More specifically, correlation between non-high-density lipoprotein and low-density lipoprotein cholesterol can be used as important markers [89]. There is an inverse relationship between HDL levels and the risk for coronary artery disease, which is independent of the low-density lipoprotein levels. Similarly, both detection and treatment of atherogenic low-density lipoproteins [231] and non-high-density lipoprotein cholesterol versus apolipoprotein B assist in cardiovascular risk stratification [232]. Thus, measuring lipid profiles total cholesterol and low-density lipoprotein-cholesterol (LDLc) levels in the fasting state in blood plasma provides more accurate diagnosis. However, triglycerides, apolipoprotein B (ApoB), and non-high-density lipoprotein cholesterol (non-HDLc) are also suitable parameters to assess cardiovascular risk and to guide lipid-lowering therapy [233]. Most commonly, clinicians easily find an explanatory detective note by using lipid estimation in both the fasting and nonfasting states. In most cases, postprandial lipid profiles in combination with ApoB are as useful as fasting lipid profiles for the differentiation between familial lipid disorders, such as heterozygous familial hypercholesterolemia, familial combined hyperlipidemia, and familial hypertriglyceridemia [233]. ATP-binding cassette transporter A1 (ABCA1) mediates cholesterol efflux to lipid-free apolipoprotein AI (ApoAI) and Apolipoprotein E (ApoE). ABCA1 is an essential regulator of high-density lipoproteins (HDLp) and reverse cholesterol transport. It also acts as an important biomarker for atherosclerosis. ABCA1, via its control of ApoE lipidation, has a role in Alzheimer's disease (AD) and has impact on A*β* deposition and clearance in AD model mice [234]. Moreover, knockdown of FKBP51 showed reduced lipid accumulation and expression of adipogenic genes. It acts as a key regulator of adipogenesis via the Akt/p38 pathway and as a potential target in the treatment of obesity and related disorders [235] (Table 2).

Exchangeable apolipoproteins play an important role in systemic lipid metabolism, especially for lipoproteins with which they are associated. Recently, emerging evidence has suggested that exchangeable apolipoproteins, such as apolipoprotein A4 (ApoA4), apolipoprotein A5 (ApoA5), apolipoprotein C3 (ApoC3), and apolipoprotein E (ApoE), also exert important effects on intracellular lipid homeostasis. There is a close link between lipid metabolism in adipose tissue and liver because the latter behaves as the metabolic sensor of dysfunctional adipose tissue and is a main target of lipotoxicity. There occurs an energy balance between these two major lipogenic organs which is intimately involved in the pathogenesis of obesity and nonalcoholic fatty liver disease (NAFLD). Moreover, intracellular function of exchangeable apolipoproteins is found to be involved in triglyceride metabolism in adipocytes and hepatocytes. These apolipoproteins may act as mediators of crosstalk between adipose tissue and liver, thus influencing development of obesity and hepatosteatosis. There is an important physiological role of exchangeable apolipoproteins and which could also develop as therapeutic markers and intervene in obesity and its related disorders [236] (Table 2).

### 7.6. Sphingolipids as Biomarkers

Sphingolipids have been recently elucidated to be not only mere components of the plasma membrane but also bioactive mediators which can induce various biological responses. Among these lipids, sphingomyelin (SM) and sphingosine-1-phosphate (Sph-1-P) are proposed to be involved in the pathogenesis of atherosclerosis [91]. SM is abundant in atherosclerotic lesions and Sph-1-P is bound to HDL and attributes to the antiatherosclerotic properties of HDL partly [91]. Therefore, Sph-1-P and SM could become useful biomarkers for atherosclerotic disorders. However, at present, the measurement of Sph-1-P and SM levels has not been brought into clinical practice, because of difficulty in measuring these sphingolipids more precisely, rapidly, and conveniently. But it is true that level of sphingolipids can more accurately predict acute coronary syndrome. Similarly, Sph-1-P and SM levels in the brain display sphingolipid metabolism that contributes to several neurological disorders. Moreover, alterations in sphingolipid metabolism contribute to several neurological disorders. Sphingolipids also affect intracellular free calcium concentration ([Ca^2+^]_i_), mobility of peptidergic secretory vesicles, and signalling pathways involved in alterations of calcium homoeostasis. Exogenously added cell-permeable sphingosine-like lipids exert complex Ca^2+^-dependent effects on astrocytes and likely alter their homeostatic function* in vivo* [90]. No doubt sphingolipids may act as future biomarkers for confirmation of atherosclerotic disorders (cardiovascular disease) [200] and several neurological lipid disorders [90] (Table 2).

### 7.7. Lipid Transfer Particles as Biomarkers

Lipoproteins contain LTPs and cholesterol and hydrocarbons as important constituents. Due to change in pH, ionic effects, temperature, and high oxidation rate induce release of LTPs from HDL which is transferred to other proteins during active flight. There are few proteins which transfer LTPs in man as well as in lower animals during speedy metabolic rate. Cholesteryl ester transfer protein (CETP) is a lipid transfer protein I (LTP I) that remains involved in the lipid regulation of lipoproteins. It is responsible for the facilitated transfer of core lipoprotein lipids, cholesteryl ester, and triglycerides and approximately one-third of the coat lipoprotein lipids, phosphatidylcholine, between different plasma lipoproteins [237]. Further, cholesterol ester transfer is mediated by lipid transfer protein as influenced by changes in the charge characteristics of plasma lipoproteins [238]. This protein also facilitates the transfer of water-insoluble drugs between different lipoprotein subclasses. Similarly, phospholipid transfer protein (PLTP), also known as lipid transfer protein-2 (LTP-2), mediates transfer of phospholipids between high-density lipoproteins (HDL) [238]. However, fluorometric assay of LTP particles may provide status of CVD disease and will work as biomarker for lipid abnormalities in man (Table 2).

Phospholipid transfer protein (PLTP) is a plasma protein that transfers phospholipids and also performs modulation of HDL particle size [238]. PLTP may be important* in vivo* in the recycling of PC from mature HDL to nascent HDL that binds to acceptors of cholesterol from peripheral tissue and participates in reverse cholesterol transport to the liver [238]. Thus, triacylglycerol transfer by LTP is induced by structural modulation of substrate-carrying lipid particles such as higher integration of apolipoproteins [239]. The substrate-specific rate of the human plasma lipid transfer protein (LTP) reaction is performed by using pyrene-labeled substrate lipid analogues which function like probes for various classes of lipids [240]. Hence, relationship between the cholesterol ester (CE) transfer activity of lipid transfer protein (LTP) and its affinity with lipid and lipoprotein particles is too important to find disease prevalence due to metabolic dysfunctions in man [241]. Activation of human plasma lipid transfer protein (LTP) is activated by apolipoproteins. Pyrene-labeled cholesteryl ester is used as a probe substrate for the transfer reaction between lipid microemulsions, of triglyceride and phosphatidylcholine [242] More often, apolipoprotein AI (ApoAI) is liberated from human high-density lipoprotein (HDL) without exposure to organic solvents or chaotropic salts by the action of isolated insect hemolymph lipid transfer particle (LTP) [243]. Thus, LTP-catalyzed vectorial lipid transfer can be used to introduce significant modifications into isolated LDL particles and become a novel mechanism to find lipid metabolomics in patients suffering from various diseases like diabetes, hypo- and/or hypercholesterolemia, and triglyceridemia, which result in changes in their plasma lipoprotein-lipid composition and concentration [237]. Modulation of substrate selectivity in plasma lipid transfer protein depends on structural variation of lipid particle [244] (Table 2).

More often, LTP-induced alteration in HDL protein density distribution depends on the LTP concentration and incubation time. Further, it is conceivable that the structural arrangement of HDL particle lipid and apoprotein components isolated from human plasma may not represent the most thermodynamically stable arrangement of lipid and protein [245]. The hydrophobic lipid components of lipoproteins, cholesteryl ester, and triglyceride are transferred between all lipoproteins by a specific plasma glycoprotein, termed lipid transfer protein (LTP). LTP facilitates lipid transfer by an exchange process in which cholesteryl ester and triglyceride compete for transfer. Thus, LTP promotes remodeling of the lipoprotein structure and plays an important role in the intravascular metabolism of these particles and in the lipoprotein-dependent pathways of cholesterol clearance from cells. The properties of LTP, its mechanisms of action, its roles in lipoprotein metabolism, and its modes of regulation are reviewed along with recent data that suggest a possible role for this protein in directly modifying cellular lipid composition [246]. Similarly, concentration of neutral lipids in the phospholipid surface of substrate particles determines lipid transfer protein activity that directly indicates metabolic status of lipids [247] (Table 2).

Human plasma lipid transfer protein (LTP) plays important role in lipoprotein metabolism and transfer endogenous cholesteryl ester and triacylglycerol. This has been confirmed that if purified human LTP is injected into rats the plasma activity increases between 1.5- and 4-fold 6 h after the injection. It causes significant increase in serum ApoB, and no significant changes in serum total cholesterol, free cholesterol, triacylglycerols, ApoAI, ApoE, or ApoAIV were noted. An increase was observed in cholesterol level in very-low density and low-density lipoproteins (VLDL and LDL) that also decrease in large-sized ApoE-rich HDL. LDL in the presence of LTP leads to the combined contribution of VLDL, LDL, and HDL to the hepatic uptake. It shows profound effects of LTP on the chemical composition of HDL subspecies, the size of HDL, and the plasma turnover and hepatic uptake of cholesteryl esters originally present in ApoAI-rich HDL [248]. In addition, a unique subclass of the high-density lipoproteins (HDL) which contains a potent lipid transfer inhibitor protein (LTIP) that inhibits cholesteryl ester, triglyceride, and phospholipid transfer mediated by the lipid transfer protein, LTP-I, and phospholipid transfer mediated by the phospholipid transfer protein, LTP-II, has been isolated. This HDL subclass not only inhibited cholesteryl ester transfer from HDL to LDL or VLDL but also inhibited cholesteryl ester transfer from HDL to HDL [249]. Thus, altered lipoprotein free cholesterol (FC) content imposes effect on the transfer of cholesteryl ester (CE) and triglyceride (TG) from very low-density (VLDL), low-density (LDL), and high-density (HDL) lipoproteins by the plasma-derived lipid transfer protein (LTP). The FC content of VLDL and HDL was selectively altered by incubating these lipoproteins with FC/phospholipid dispersions of varying composition. Lipid transfer between lipoproteins is integral to the process of reverse cholesterol transport, in which lipoprotein FC levels are a potent, positive regulator of the pathways involved in sterol clearance. FC may modulate lipid transfer by altering the availability of CE and TG (triglyceride) to LTP (lipid transfer protein) at the lipoprotein surface [250]. In human two lipid transfer proteins have been purified and immunoprecipitated [251]. These are designated as lipid transfer proteins 1 and 2 having molecular weight Mr 69,000 and 55,000 each of which facilitates the transfer of radiolabelled cholesteryl ester, triacylglycerol, and phosphatidylcholine between plasma lipoproteins. Besides these two lipoproteins, a high-density lipoprotein also serves as cholesterol carrier in human small intestine epithelial cells. The receptor binding and interactions also depend on the lipid composition of the lipoprotein [252]. Lipid transfer particle formation is reversible [253–255] and its catabolism depends on the ligand size [92, 256] (Table 2). A slow transportation of lipid also indicates an increase in cholesterol amount and slow sequestration of cholesterol that shows severe lipid abnormalities.

### 7.8. Serum Ferritin Level

Serum ferritin (SF) level is an important parameter because it has relationship with aggregation of metabolic disorders in nondiabetic elderly patients [93]. It could work in association with various parameters, including blood pressure (BP), height, weight, lipid profiles, blood glucose (BG), body mass index (BMI), fasting insulin (FINS), serum uric acid (SUA), and the urinary albumin/creatinine ratio (UACR) for prediction of CVD risks in man. By including other parameters, such as insulin resistance (HOMA-IR), *β*-cell function (HOMA-*β*), quantitative insulin sensitivity check index (QUICKI), and disposition index (DI), a number of lipid metabolic disorders or abnormalities can be established [93] (Li et al., 2014). The SF levels in nondiabetic, elderly individuals with metabolic disorders may be significantly related to the clustering of the metabolic disorders. Dyslipidemia, obesity, disorders of purine metabolism, and insulin resistance may be important risk factors for higher SF levels in the elderly [93] (Table 2).

### 7.9. Plasma Levels of High-Density Lipoprotein Cholesterol (HDLc)

The plasma levels of high-density lipoprotein cholesterol (HDLc) have an inverse relationship to the risks of atherosclerosis and cardiovascular disease (CVD) [66]. A novel locus is identified in genome for HDL that could potentially do biological regulation of HDL metabolism in healthy-longevous subjects. A possible regulatory variant upstream of NLRP1 that is associated with HDL in these elderly Long Life Family Study (LLFS) subjects may also contribute to their longevity and health. NLRP1 intergenic SNPs show a potential regulatory function in Encyclopedia of DNA Elements (ENCODE). NLRP1 plays an important role in the induction of apoptosis, and its inflammasome is critical for mediating innate immune responses. NLRP1 intergenic SNPs have also been associated with immunity/inflammasome disorders which highlights the biological importance of this chromosomal region [66]. In addition, Framingham risk score (FRS) provides a practical approach for using clinical information, basic biochemical tests, and more specialized tests, such as carotid ultrasound and coronary artery calcium scoring, to identify groups of patients at greater risk for atherosclerotic cardiovascular disease than suggested by the FRS [66] (Table 2).

### 7.10. Immunohistochemical Biomarkers

A number of immunohistochemical markers are to be used in histological diagnosis of the heart diseases and valvular impairments. No doubt, blood and cardiac muscle functions may ease finding of pathological changes and sudden cardiac death [257]. Immunohistochemical methods are used to find structural and functional abnormalities of a slow or fast beating heart [257]. Therefore, clinic-pathological studies of primary cardiac sarcoma or intimal sarcoma by using immunohistochemical analysis, fluorescence* in situ* hybridization, real-time polymerase chain reaction, and array-comparative genomic hybridization may provide functional abnormalities. Similarly, immunohistochemical detection of MDM2 overexpression can easily detect intimal sarcoma that also confirms molecular aberration [94]. Histopathologic examination of the aortic arch showed significant information regarding atherosclerotic lesion [11]. Serum MFAP4 acts as a biomarker of stable atherosclerotic disease [95]. Similarly, localization of microfibrillar-associated protein-4 (MFAP4) in human tissues and clinical evaluation of serum MFAP4 are associated with various cardiovascular conditions [95]. Similarly, N-cadherin and connexin-43 are used in the diagnosis of arrhythmogenic right ventricular cardiomyopathy (ARVC) [96]. However, for diagnosis of ARVC plakoglobin is used in immunohistochemical analysis with a relatively high sensitivity and specificity in ARVC [96]. Intravascular papillary endothelial hyperplasia (IPEH) is a benign intravascular pathogenesis with features mimicking other benign and malignant vascular proliferations. However, histomorphological feature of IPEH can display papillary structure covered with hyperplastic endothelial cells within the vascular lumen [97] (Table 2).

Similarly, immunohistochemical and ultramicroscopic analyses of presence of different associations of proteins to lipids function as antigens in adipocytes as well as inside intimedia of arterial wall. This can be localized and lipid protein association could be used as antigens to develop good biomarkers for CVDs. More often, antibodies generated against such antigens may act as good therapeutic biomarkers which can also display disease state in the animals as well as in human beings. However, immunocytochemical localization of lipid binding proteins, factors, or any other lipid related disorders can be established by using polyclonal antibody specific to CBPs (cholesterol-carrying proteins) in immunofluorescence and immunoblot techniques to detect the cellular location of these proteins in various tissues. Therefore, presence of large number of CBPs or cholesterol-carrying proteins or intracellular zymogene like dense granules scattered throughout the apical area of microvillus cells also indicates presence of CVDs. Presence of these proteins can be detected by SDS-PAGE gel electrophoresis and immunoblotting and immunocytochemical methods which clearly display presence of large amount of cholesterol trafficking and its absorption and deposition in arterial walls as well as in circulating blood in form of LDL or HDL cholesterol. Interestingly, lipid protein ratio if confirmed by immunohistochemical methods may assist in numeration of CVDs severity at an earlier stage (Table 2).

Lipoproteins also serve as carrier vehicles of sterols [212, 258] and assist in transport of different types of lipids, that is, diacylglycerol [259], phospholipids [260], cholesterol [98, 261], and hydrocarbons [251, 262]. These proteins also accelerate the transfer or exchange of lipids [263] across/between the membranes [258, 264] and bind to surface receptors [265, 266]. These proteins also play important role in intracellular distribution of cholesterol [87, 98, 267] and maintain cholesterol homeostasis inside cells [268]. If immunohistochemical localization of cholesterol binding proteins (StarD5 cholesterol binding proteins) in human tissues mainly in liver cells [99] and sterol regulatory element binding protein-2 in rat brain [101] is being done, it can assist in tracking the status of CVDs in pregnant women (Table 2).

Additionally, Western blot and immunohistochemical analysis of Rho, Rho-kinase 1 (ROCK1), and ROCK2 expression and phosphorylation of myosin phosphatase may adjudge myocardial I/R ratio which predict suppression of myocardial apoptosis and inflammation by inhibiting the Rho/ROCK pathway [100]. Angiogenic action of thrombin in rabbit model can display acute myocardial infarction on the basis of histopathologic analysis, immunohistochemical staining for endothelial markers CD31 and vascular endothelial growth factor-A, and electron microscopy examination performed on excised hearts. Intramyocardial administration of thrombin seems to promote angiogenesis and improve cardiac function of the ischemic myocardium, which may provide a new therapeutic approach in patients with myocardial ischemia [269] (Table 2).

### 7.11. Oxidative Stress-Related Biomarkers

Intensified oxidative modification of proteins and increased concentration of advanced oxidation protein products (AOPPs) are used as biomarkers for identifying different pathological states, especially etiopathogenesis. These biomarkers contribute to biochemical and clinical changes undergoing the condition of increased OS (oxidative stress) and are of high prognostic utility. These biomarkers easily indicate status of diabetes and its complications (diabetic nephropathy) with additional risk of cardiovascular diseases [102]. Similarly, circulating OGN and NGAL/MMP9 complex are promising biomarkers that are expressed in vulnerable atherosclerotic plaques and may have incremental value for prediction of MACE within 1 year after coronary angiography [103]. In elderly prediabetics, SOD-1 and TAS seem to reflect the first symptoms of oxidative stress, while TBARS are later biomarkers of oxidative stress [105]. No doubt, oxidative stress indicates high risk of developing CVD mostly related to carbohydrate metabolism disorders. Endothelial dysfunction in chronic inflammatory diseases [270] is also related to increased plasma DPP4 activities [271] and vitamin D [272]. Oxidative stress is a concomitant factor in the pathogenesis of MetS that induces oxidative PTM as carbonylation which is genetically induced [104] (Table 2).

The brain is known to be sensitive to oxidative stress and lipid peroxidation which contribute too many disease processes. It is directly related to psychiatric illness mainly schizophrenia, bipolar disorder, and major depressive disorder. Therefore, lipid peroxidation uncovers therapeutic targets and acts as biomarkers of psychiatric disease [273]. Central nervous system is the most metabolically active organ of the body characterized by high requirement for oxygen and relatively low antioxidative activity. Lipid peroxidation makes neurons and glia highly susceptible to destruction by reactive oxygen/nitrogen species and neurodegeneration [106]. Moreover, both F2-IsoPs and F4-NPs act as markers of lipid peroxidation caused by the free radicals which causes oxidative stress in the pathogenesis of human neurodegenerative diseases [106]. These are confirmed as novel biomarkers of oxidative stress and are advantageous in monitoring their formation to better display the involvement of oxidative stress in neurological diseases [106]. Similarly, oxidative stress plays a key role in the pathogenesis of diabetic cardiomyopathy (DCM). However, cardiac structure changes, apoptosis, superoxide production, NADPH oxidase subunits expression (gp91phox, p47phox, and p67phox), and related regulatory factors such as high blood glucose level and reduced body weight are considered to be good markers of DCM. FPE may have therapeutic potential for STZ-induced diabetic cardiomyopathy through preventing myocardial apoptosis via attenuation of oxidative stress that is probably mediated by JNK and P38 MAPK signaling pathways [176] (Table 2).

### 7.12. Inflammatory Biomarkers

Inflammation plays important roles at all stages of atherosclerosis. It is operated by cell-secreted factors due to antigenic interactions; hence, inflammatory biomarkers are considered potential biomarkers for predicting cardiovascular disease [107]. Inflammatory molecules secreted after antigen or pathogen interactions of oxidation of lipids from various immune cells are considered to be the best markers. Different types of chemokines, chemokine receptors, and inflammatory lipids act as important biomarkers in atherosclerosis. Therefore, both inflammation and endothelial dysfunction have been implicated in the pathogenesis of atherosclerotic vascular disease [28]. Similarly, inflammation can predict recurrent cardiovascular events in CAD patients. In addition, some other noble markers are LDL-C assessment with proprotein convertase subtilisin kexin type 9 monoclonal antibody can predict thrombolysis in myocardial infarction [112]. Similarly, *γ*′ fibrinogen acts as novel marker of thrombotic disease [109] while interleukin-1 receptor-like 1 (ST2) and interleukin-33 are important markers which are used to determine and assess cardiovascular risk [110]. Interleukin-1 receptor-1 (IL1R1) and its ligand, IL1*β*, are upregulated in cardiovascular disease, obesity, and infection. Moreover, higher level of IL1R1 transcripts in platelets from obese individuals of the Framingham Heart Study (FHS) display disease status. Additionally, IL1*β* levels are increased in atherosclerotic plaques and in bacterial infections. Both IL1*β* and IL1R1 activate platelets and megakaryocytes to promote atherothrombosis [111]. These minor chemokines could develop as highly sensitive biomarkers to predict cardiac problems by using in different bioassays [274] (Table 2).

Chemokines are a diverse group of molecules with important implications for the development of solid tissues and normal function of the immune system [108]. Chemokines play important roles in atherosclerotic vascular disease. These are expressed not only by cells of the vessel wall but also by emigrated leukocytes. These are secreted at the sites of inflammation and exert multiple functions beyond cell recruitment. However, change in the chemokine concentration is possible after involvement of a complex system that can have important and dangerous consequences leading to diseases. The specific implications of the various chemokines in CV diseases have been elucidated [108]. Chemokine systems have major effects on the initiation and progression of atherosclerosis by controlling the trafficking of inflammatory cells* in vivo* through interaction with their receptors. More often, population of plasmacytoid dendritic cells in peripheral blood works as important cellular biomarkers of atherosclerosis [275]. Elevated IL-6 levels may reflect a state that promotes vascular inflammation and development of subclinical atherosclerosis independent of traditional cardiovascular risk factors [28] and play a vital role for complement in heart disease [276]. Similarly, complement C3 is used as a marker of cardiometabolic risk in psoriasis [113]. More often, increased levels of the proinflammatory cytokine IL-6 are associated with impaired endothelial function which is assessed by FMD (flow-mediated dilation).

Chemokine receptor-5 (CCR5) has been reported to be an active participant in the late stage of atherosclerosis and has the potential as a prognostic biomarker for plaque stability [114]. Few other important inflammatory biomarkers are chemokine CCL2, CCL3, CCL5, and CXCL10 which are expressed during early inflammatory process [115]. Similarly, IL-1*β*, RAGE, and FABP4 are good markers of metabolic inflammation and related pathologies [116, 277]. The cytokine mainly midkine supports neutrophil trafficking during acute inflammation by promoting adhesion via *β*2 integrins (CD11/CD18) [278]. Similarly, atypical chemokine receptors are also important markers of CAD [117]. However, both pre- and procytokines which work as basis of inflammatory mediators of the disease process can be used as additional biomarkers in various bioassays. Therefore, systemic levels of adhesion molecules (VCAM-1, ICAM-1) chemokines (MCP-1) and cytokines (IL-1, TNF-*α*) can better assist in assessment of CV related risks [117]. The secreted chemokines also act as biomarkers of hypertension [279] and inflammation/oxidation markers are considered to be best makers from disease diagnosis point of view [280]. Hence, both secretory and circulating levels of inflammatory factors are proved important biomarkers [281] (Table 2).

Similarly, very high levels of CRP are routinely used to measure disease activity in patients with chronic inflammatory syndromes. C-reactive protein (CRP) is one of the most important biomarkers for arteriosclerosis and cardiovascular disease [118]. More often, elevated levels of CRP detected by high sensitivity assay have been reported to predict cardiovascular outcome in population studies of both primary and secondary preventions. CRP affects cell cycle and inflammatory process in cardiac myocytes [282]. Moreover, baseline serum C-reactive protein (CRP) [282] acts as a marker of systemic inflammation, secreted mainly by the liver in response to stimulation by IL-6. High-sensitivity CRP has been increasingly employed in algorithms to predict risk in addition to conventional risk factors (low < 1 mg/L, intermediate 1–3 mg/L, and high > 3 mg/L). Other than traditional Framingham risk score, Reynolds Risk Score integrates CRP measurements that reports improved risk prediction. It remains to be determined whether CRP plays a role in atherosclerosis or is simply a marker of inflammation. C-reactive proteins, fibrinogen, and platelet activation factors are also used in prediction [283] of acute coronary syndromes in cardiovascular diseases [284]. Further understanding of homeostatic macrophage function may lead to new therapeutic approaches to treat ischemia, hypertension, and metabolic disorders [285]. However, relationship among changes in C-reactive protein and cardiovascular disease risk factors with lifestyle interventions can predict the disease [126]. On the other hand, inflammatory lipids are biologically active molecules with crucial impacts on the function of various cell types, including immune cells in health and disease, mainly chronic inflammation in the case of atherosclerosis [108] (Table 2).

Myeloperoxidase (MPO) is a leukocyte-derived prooxidant enzyme that is released from granules of neutrophils and monocytes [119]. MPO and its oxidant products, nitrotyrosine, and chlorotyrosine have been identified in atherosclerotic plaque and at the site of plaque rupture and play important role in the genesis of atherosclerosis [119]. MPO promotes a number of pathological events involved in plaque formation and rupture, including uptake of oxidized lipid by macrophages, impaired nitric oxide bioavailability. MPO levels independently predict outcomes in patients presenting with acute coronary syndromes or for evaluation of chest pain of suspected cardiac etiology and endothelial dysfunction [119]. Platelet-activating factor acetylhydrolase (PAF-AH) is related to oxidation of phospholipids, and it is widely accepted as anti-inflammatory enzyme that exists in multimolecular forms and acts as an inflammatory markers [119] (Table 2).

Lipid peroxidation also takes place in neurons and damages it. Neurons are especially susceptible to oxidative stress changing signal transduction and information processing mechanisms [286]. Posttraumatic stress disorder (PTSD) is a complex of symptoms developed in a patient after traumatic event. It causes hyperactivation of neurons under stress factors influence, so-called excitotoxicity, followed by oxidative stress (OS) because of an accumulation of free radicals [286]. PTEN is a lipid and protein phosphatase that regulates a diverse range of cellular mechanisms. PTEN is mainly present in the cytosol and transiently associates with the plasma membrane to dephosphorylate PI(3,4,5)P3, thereby antagonizing the PI3-kinase signaling pathway. Compartmentalized PTEN has a role in developing and mature neurons in health and disease [287]. Inflammatory expression profiles in monocyte to macrophage differentiation in patients with systemic lupus erythematosus are deeply associated with atherosclerosis [288].

There are so many known emerging biomarkers for acute heart conditions [121] such as acute myocardial infarction and heart failure. These are associated with significant morbidity and mortality but were found to be slow predictors [121]. Hence, for rapid diagnosis based on risk stratification, it needs strong biomarkers. However, inflammatory factors such as ICAM-1, VCAM-1, TNF-*α*, IL-6, and CRP play key roles in mediating vascular inflammation and blocking RAAS negatively modulates the levels of these inflammatory molecules [128]. Some of these inflammatory markers are clinically associated with CVD events. However, more efforts are to be required to establish long-term effects of RAAS (renin-angiotensin-aldosterone system) inhibition on vascular inflammation, vascular cells regeneration, and CVD clinical outcomes. RAAS has important role in vascular inflammation, mainly vascular cells response to RAAS. Thus, inhibition of RAAS signaling can predict vascular inflammation-associated CVDs [128]. However, novel biomarkers are to be needed which could predict and assist in tailoring of appropriate therapy to high-risk patients. Galectin-3 is an active biomarker found in inflammatory and fibrotic processes and is a marker of mortality (Table 2).

### 7.13. ST2 as a Cardiovascular Risk Biomarker

ST2, is a member of the interleukin-1 receptor family, is released from cardiac myocytes under mechanical strain. It exists in a transmembrane (ST2L) and a soluble form (sST2) due to alternative splicing. Soluble ST2 (sST2) concentrations are associated with adverse cardiac events in high-risk cohorts [289]. Soluble ST2 (sST2), a member of the IL-1 receptor family, has been proposed as a novel biomarker with predictive value for heart failure and mortality in patients suffering from cardiovascular diseases [110]. Association of sST2 with all-cause and cardiovascular mortality in a large show low-risk population-based cohort. In a low-risk population, sST2 does not associate with traditional cardiovascular risk factors or nonfatal cardiovascular events but it was found higher in African Americans and is associated with increased all-cause and cardiovascular mortality [289]. Several groups have reported sST2 elevations in serum of cardiovascular disease patients [110]. There is consisting evidence that sST2 is independently predictive for mortality in patients with heart failure or myocardial infarction. In addition to its potential as a biomarker for adverse cardiovascular events, ST2 is considered to play a causal role in chronic cardiovascular diseases such as atherosclerosis and heart failure [110]. sST2 is a biomarker for adverse cardiovascular events. It also plays a potential mechanistic role in IL-33/ST2 pathway in chronic cardiovascular diseases such as atherosclerosis and heart failure [110]. Cardiovascular interventions affect clinical characteristics on plasma sST2 expression levels. Hence, strong determinant of sST2 levels needs to be taken into account when exploring sST2 as predictor of future cardiovascular events [110] (Table 2).

Soluble ST2 (sST2), an interleukin-1 receptor family member, is an emerging risk indicator for patients with cardiovascular disease. Soluble ST2 is a marker of biomechanical strain for which the natural ligand is interleukin-33 (IL-33) [120]. These are used predictive markers for non-ST-elevation myocardial infarction (NSTEMI). Moreover, elevated ST2 predicts adverse outcome in non-ST-elevation myocardial infarction but does not significantly improve risk stratification for established markers. Interleukin-33 was not related to adverse events [120]. More often, concentrations of serum biomarkers (sST2), amino-terminal proB-type natriuretic peptide (NT-proBNP), and transthoracic echocardiography and sST2 in combination are used as strong predictor of mortality in patients presenting with acute dyspnea, particularly those with preserved LVEF, and may be useful for triage and risk stratification of this challenging group [290]. ST2 has prognostic utility in patients with acute dyspnea and preserved left ventricular ejection fraction [290]. Soluble ST2 reflects activity of an interleukin-33-dependent cardioprotective signaling axis and is a diagnostic and prognostic marker in acute heart failure [291]. ST2 acts as an important cardiovascular risk biomarker [122]. It is a potent marker of risk in chronic heart failure and when used in combination with NT-proBNP offers moderate improvement in assessing prognosis beyond clinical risk scores. High-sensitivity ST2 is used for prediction of adverse outcomes in chronic heart failure [291]. ST2 is released by stressed cardiac myocytes and also predicts mortality in heart failure and myocardial infarction. Copeptin is a stable arginine vasopressin precursor associated with increased risk of heart failure. It may also be useful to exclude acute myocardial infarction [121] (Table 2).

ST2 is a member of the interleukin-1 (IL-1) receptor family discovered in a classical translational science fashion that exists in two forms, a transmembrane receptor (ST2L) as well as a soluble decoy receptor (sST2) [122]. The ligand of ST2 is IL-33, which is involved in reducing fibrosis and hypertrophy in mechanically strained tissues. In* in vitro* and* in vivo* models, ST2L transduces the effects of IL-33, while excess sST2 or abnormalities in ST2 signaling leads to cardiac hypertrophy, fibrosis, and ventricular dysfunction [122]. Clinically, in patients with symptomatic heart failure (HF), elevated concentrations of sST2 are strongly associated with severity of the diagnosis and powerfully predict increased risk of complications, independent of other established or emerging biomarkers. sST2 testing has also been shown to predict onset of symptomatic HF in patients with acute myocardial infarction, while in community-based subjects, sST2 values independently predict future HF, cardiovascular disease events, and mortality (Table 2).

Soluble ST2 is a marker of cellular stress and injury whose natural ligand is interleukin-33. Elevated ST2 and IL-33 were both associated with increased mortality. ST2 demonstrated incremental value over contemporary risk markers but IL-33 did not. ST2 has a potential role in risk stratification using a multimarker approach [123]. ST2 is involved in cardioprotective signaling in the myocardium and has been identified as a potentially promising biomarker in heart failure (HF) [292]. More specifically, ST2 level of patients also clearly display the heart failures. ST2 was modestly associated with functional capacity and was significantly associated with outcomes in a well-treated cohort of ambulatory patients with HF although it did not significantly affect reclassification of risk [292]. ST2 and galectin-3 (Gal-3) are compared head-to-head for long-term risk stratification in an ambulatory heart failure (HF) population [293]. HF risk is also associated with other risk factors including N-terminal proB-type natriuretic peptide [293] (Table 2).

ST2 and Gal-3 are promising biomarkers of myocardial fibrosis and remodeling in HF. Increased soluble ST2 predicts long-term mortality in patients with stable coronary artery disease [294]. There is a prognostic performance of multiple biomarkers in patients with non-ST-segment elevation acute coronary syndrome [33]. Circulating concentrations of soluble ST2, growth differentiation factor-15 (GDF-15), and high-sensitivity troponin I (hsTnI) are associated with incident atrial fibrillation (AF). These biomarkers improve current risk prediction models including AF risk factors, B-type natriuretic peptide (BNP), and C-reactive protein (CRP) [124]. Moreover, prognostic performance of ST2 is associated with cardiovascular death (CVD) and heart failure (HF) in patients with non-ST-elevation acute coronary syndrome (NSTE-ACS). ST2 has predictive role in non-ST-elevation acute coronary syndrome [295]. Myocytes that are subjected to mechanical stress secrete ST2, a soluble interleukin-1 receptor family member that is associated with HF after STE-ACS. ST2 correlates weakly with biomarkers of acute injury and hemodynamic stress but is strongly associated with the risk of HF after NSTE-ACS. This biomarker and related pathway merit potential therapeutic targets for patients with ACS at risk for cardiac remodeling [295]. Growth differentiation factor-15 (GDF-15), soluble ST2 (sST2), and high-sensitivity troponin I (hsTnI) are emerging predictors of adverse clinical outcomes [125]. More often, circulating concentrations are related to the development of kidney disease in the community. Higher circulating GDF-15 is associated with incident renal outcomes and improves risk prediction of incident CKD. GDF-15 also indicates renal injury that may be associated with cardiovascular stress or CVD [125] (Table 2).

In a community-based cohort, circulating hsTnI concentrations were associated with incident AF (atrial filtration). None of the novel biomarkers evaluated improved AF risk discrimination or reclassification beyond standard clinical AF risk factors and biomarkers. Copeptin (C-terminal provasopressin), MR-proADM (midregional proadrenomedullin), and MR-proANP (midregional proatrial natriuretic peptide) are complementary prognostic markers for CV death and HF in patients. These are emerging biomarkers which show wider application for therapeutic decision making [33]. Soluble suppression of tumorigenicity 2 (sST2) has emerged as a strong prognostic biomarker in patients with heart failure and myocardial infarction. Similarly, cardiac stress biomarkers are associated with LVH (left ventricular hypertrophy) and LVSD (left ventricular systolic dysfunction) but may have limited clinical utility as screening tools [134]. Thus, integration of biomarkers could predict cardiovascular stress with left ventricular hypertrophy and dysfunction [134] (Table 2).

Biomarkers for predicting cardiovascular events in community-based populations have not consistently added information to standard risk factors [296]. Thus, prognostic utility of novel biomarkers may predict cardiovascular stress [296]. To determine the prognostic value of 3 novel biomarkers such as soluble ST2, growth differentiation factor-15, and high-sensitivity troponin I, they are used to find cardiovascular stress, in the Framingham Heart Study. For better investigation of disease, combined ability of the biomarkers should be utilized to predict adverse outcomes, for which a multimarker score composed of the 3 biomarkers in addition to B-type natriuretic peptide and high-sensitivity C-reactive protein is to be prepared. Multiple biomarkers of cardiovascular stress are detectable in ambulatory individuals and add prognostic value to standard risk factors for predicting death and overall cardiovascular events [296] (Table 2).

## 8. Imaging Biomarkers

Imaging plays important role in diagnosis and classification of acute stroke. Imaging biomarkers assist in the prediction of the clinical outcome and the risk of hemorrhagic complications [65]. However, multimodal imaging is used as diagnostics based on anatomical findings which enable characterization of the ischemic brain tissue and the cerebral hemodynamic state. More often, perfusion imaging also enables the detection and quantification of the irreversibly damaged infarct tissues at risk [65]. Thus, parameters derived from perfusion studies can serve as surrogate markers for stroke severity and the occurrence of hemorrhagic complications. More often, ^31^P magnetic resonance spectroscopy (MRS) and magnetic resonance imaging (MRI) are used to investigate cardiac energy status and function. Similarly, carotid ultrasound and coronary artery calcium scoring are used to identify groups of patients at greater risk for atherosclerotic cardiovascular disease than suggested by the FRS (Framingham risk score) [297]. Imaging also assists patients with diabetes mellitus (DM) which are at severely increased risk of developing atherosclerosis. Moreover, assessment of carotid atherosclerosis can be made by using intraplaque neovascularization and plaque ulceration using quantitative contrast-enhanced ultrasound in asymptomatic patients with diabetes mellitus [67]. Intraplaque neovascularization (IPN) and plaque ulceration are markers of the vulnerable plaque, which also show an increased risk of rupture that may lead to cardiovascular events. Similarly, prevalence of subclinical carotid atherosclerosis, intraplaque neovascularization (IPN), and plaque ulceration in asymptomatic patients with DM can be established by quantitative contrast-enhanced ultrasound, magnetic resonance imaging (MRI), and ultrasound (US) and computed tomography (CT) (Table 2). Moreover, anatomical imaging is done by using ultrasound (US) and computed tomography (CT) to predict CVD events. Molecular imaging is also done by using magnetic resonance (MR), near-infrared fluorescence (NIRF), bioluminescence, single-photon emission computed tomography (SPECT), and positron emission tomography (PET) to establish potential disease mechanisms and the development of advanced molecular imaging techniques [68].

However, cardiac and skeletal magnetic resonance imaging is applied for the early detection and follow-up of myocardial and skeletal muscle tissue changes (oedema, inflammation, and fibrosis) in IIM [69]. Similarly, association between left atrial (LA) volume and function measured with feature-tracking cardiac magnetic resonance (CMR) cleary display development of heart failure (HF) in asymptomatic individuals [298]. Lower global PLAS and higher LAVImin, measured using CMR feature tracking, are used as independent markers to notice HF incidents in a multiethnic population of asymptomatic individuals [298]. Thus, imaging modalities and serum biomarkers can establish pathophysiology of the unstable carotid plaque in high-risk stroke patients [71]. Changes in aortic wall material properties, such as stiffness, have been shown to accompany onset and progression of various cardiovascular pathologies [72]. Similarly, pulse wave velocity (PWV) and propagation along the aortic wall have been shown to depend on the wall stiffness (i.e., stiffer the wall, higher the PWV) and can potentially enhance the noninvasive diagnostic techniques. However, conventional clinical methods involve a global examination of the pulse traveling between femoral and carotid arteries that provide an average PWV estimate. Such methods may not prove effective in detecting wall focal changes as entailed by a range of cardiovascular diseases [72]. More often, morphological and physiological assessment of the valve and ascending aorta is performed with transthoracic echocardiography and MRI. BAV is associated with endothelial dysfunction [73] and shows correlations with inflammatory markers and endothelial progenitor cell counts [73]. Therefore, interactions between inflammatory activation and endothelial dysfunction selectively modulate valve disease progression in patients with bicuspid aortic valve [73]. Bicuspid aortic valve (BAV) is associated with increased risk of valvular degeneration and ascending aortic aneurysm formation and rupture. The evaluation of diffuse myocardial fibrosis has a great potential in large-scale diseases, including their initial phases, with the possibility to identify patients at risk for subsequent development of clinical heart failure. It is also used to assess repeatedly the stage and progression of cardiac diseases and to monitor the effect of treatment. Cardiovascular magnetic resonance using late gadolinium enhancement (LGE) provides assessment of myocardial tissue* in vivo* [299]. Takayasu arteritis (TA) is a chronic granulomatous vasculitis of autoimmune etiology characterized by narrowing of aorta and its main branches. It is one of the rarest diseases but the third common cause of vasculitis in pediatric age group, clinical features, angiographic findings, and outcome of children with TA referred to a single centre in South India over a 9-year period (Table 2).

Coronary angiography is used to detect and quantify the extent of obstructive coronary artery disease. Angiography guides the use of medical and revascularization strategies. Moreover, the number of diseased vessels predicts the subsequent incidence of clinical events in large registries. The presence of multivessel disease indicates patients who are likely to benefit from bypass surgery. However, angiography is limited as it requires an invasive procedure, yields images of the lumen, not the vessel wall, and provides no information on plaque composition. Noninvasive ultrasonic imaging permits measurement of intimal-medial thickness (IMT) in the carotid and femoral arteries. However, increasing carotid IMT is associated with risk factors, atherosclerosis in other vascular territories, and clinical events. This type of imaging can be applied in large populations spanning a broad range of cardiovascular risk. It detects early changes in the artery wall or sometimes remains unclear and lumen gets deposited and it is investigated in advance stage or mature atherosclerosis at the specific site. Intravascular ultrasound (IVUS) places a high-frequency ultrasound transducer in close proximity to the endothelial surface, generating high-resolution images of the entire vessel wall. Imaging throughout the length of an arterial segment permits precise volumetric measurement of the extent of disease (Table 2).

Detection and quantitation of coronary calcification by CT correlate with the extent of coronary artery disease and prospective risk of clinical events. CT detects lumen stenoses, with particular accuracy in saphenous vein grafts. Hence, characterization of the remodeling pattern of the artery wall and the multifocal nature of plaque rupture assist in finding acute coronary syndromes. Further, increasing resolution permits visualization of the coronary arteries by computer tomography (CT). Thus, ongoing technological advances, which improve imaging resolution, may permit visualization of plaque within the vessel wall. However, imaging shows limitations such as radiation exposure, requirement for heart rate control, and intravenous contrast imaging. Imaging is poor in regions containing stents. More often, magnetic resonance (MR) characterizes the extent and composition of atherosclerosis in the aorta and carotid arteries. Suboptimal resolution currently limits precise imaging in the coronary arteries. Advances in intravascular imaging, supported by the use of vascular coils, may permit coronary imaging. A number of novel intravascular modalities provide alternative techniques for characterization of coronary atherosclerosis. Optical coherence tomography (OCT) detects reflected light and generates a high-resolution image of the artery well. OCT detects the degree of inflammatory activity within plaque and measures the thickness of the fibrous cap. Palpography and elastography determine strain of fibrous caps overlying atherosclerotic plaque while thermography demonstrates increase in temperature at the site of inflamed, vulnerable plaques. Development of molecular-targeted imaging enables the characterization of pathological cascades involved in plaque formation and rupture.

## 9. Physiological Markers

Multiple physiological factors are involved in the etiology of cardiovascular disease (CVD). Pathological changes occur in a variety of cell types long before symptoms become apparent and diagnosis is made. Dysregulation of physiological functions is associated with the activation of immune cells, leading to local and finally systemic inflammation that is characterized by production of high levels of reactive oxygen species (ROS). Patients suffering from inflammatory diseases often present with diminished levels of antioxidants either due to insufficient dietary intake or, and even more likely, due to increased demand in situations of overwhelming ROS production by activated immune effector cells like macrophages [127]. Besides this, physiological disbalances also occur due to alterations in the level of proteins, amino acids, carbohydrates, lipids, enzymes, vitamins, and metal ions. More often, metabolic disbalances play a major role in disease onset and progression. Several central signaling pathways involved in the regulation of immunological, metabolic, and endothelial function are regulated in a redox-sensitive manner. During cellular immune response, interferon *γ*-dependent pathways are activated such as tryptophan breakdown by the enzyme indoleamine 2,3-dioxygenase (IDO) in monocyte-derived macrophages, fibroblasts, and endothelial and epithelial cells. Neopterin, a marker of oxidative stress and immune activation, is produced by GTP-cyclohydrolase * *I * *in macrophages and dendritic cells. Nitric oxide synthase (NOS) is induced in several cell types to generate nitric oxide (NO). NO, despite its low reactivity, is a potent antioxidant involved in the regulation of the vasomotor tone and of immunomodulatory signaling pathways. NO inhibits the expression and function of IDO [127]. The RAAS through its physiological effectors plays a key role in promoting and maintaining inflammation. Inflammation is an important mechanism in the development and progression of CVD such as hypertension and atherosclerosis. It plays main role in regulating blood pressure in hypertension, RAAS has proinflammatory and profibrotic effects at cellular and molecular levels, and, therefore, blocking RAAS provides beneficial effects for the treatment of cardiovascular and renal diseases. Interestingly, inhibition of RAAS (renin-angiotensin-aldosterone system) positively influences vascular functions and improving CVD outcomes. The beneficial vascular effects of RAAS inhibition are likely due to decreasing vascular inflammation, oxidative stress, endothelial dysfunction, and positive effects on regeneration of endothelial progenitor cells [300] (Table 2).

Function of NOS requires the cofactor tetrahydrobiopterin (BH4), which is produced in humans primarily by fibroblasts and endothelial cells. Highly toxic peroxynitrite (ONOO(−)) is formed solely in the presence of superoxide anion (O_2_ (−)). Neopterin and kynurenine to tryptophan ratio (Kyn/Trp), as an estimate of IDO enzyme activity, are robust markers of immune activation* in vitro* and* in vivo*. Both of these diagnostic parameters are able to predict cardiovascular and overall mortality in patients at risk [127]. There occurs a positive association between fibrinogen levels and risk of cardiovascular diseases or risk of bleeding (e.g., DIC) when screening for possible acquired or congenital deficiency [301]. W.H.O recognized International Standard (IS) have been approved for Fibrinogen Plasma and commercial fibrinogen assay kits are available in market for for diagnosing CVD risks in patients [301] (Table 2).

Similarly, salivary diagnostics are also developed by utilizing techniques for identifying saliva-based molecular indicators of disease [302]. Moreover, oral fluids were found to be easily accessible noninvasive alternative to traditional diagnostic avenues and not just an essential component of the digestive process [302]. Determining saliva as a credible means of evaluating health status represents a considerable leap forward in health care, one that could lead to enormous translational advantages and significant clinical outcomes [302]. Similarly, renin-angiotensin system (RAS) plays a crucial role in the regulation of physiological homeostasis and diseases such as hypertension, coronary artery disease, and chronic renal failure. In this cascade, the angiotensin-converting enzyme (ACE)/angiotensin II (Ang II)/AT1 receptor axis induces pathological effects, such as vasoconstriction, cell proliferation, and fibrosis, while the ACE2/Ang-(1-7)/Mas receptor axis is protective for end-organ damage [129]. It is true that altered expression profile of the ACE2/Ang-(1-7)/Mas axis in the heart and the kidney of bGH mice could contribute to the increased incidence of hypertension, cardiovascular, and renal alterations in animal models [129] (Table 2).

Lipoproteins were also found to be associated with an increased incidence of cardiovascular diseases for decades. Moreover, these lipid carrier proteins perform physiological function and have role in the development of cardiovascular diseases [303]. ErbB2 is an interacting protein (Erbin which is widely expressed and participates in inhibition of several intracellular signaling pathways). Its mRNA has been found to be present in relatively high levels in the heart. Erbin has its important physiological role in the heart mainly in cardiac hypertrophy because it is an inhibitor of pathological cardiac hypertrophy, and its inhibition is mediated by modulating ERK signaling [130]. Endothelial MPs (EMPs) are also confirmed as potential biomarkers for COPD [131]. More specifically, circulating MPs also have several physiological functions* in vivo*, such as intercellular crosstalk, and increase in EMPs in COPD seems to play a role in the pathophysiology of this disease [131]. COPD patients show endothelial injury in the pulmonary capillary vasculature that leads to lung destruction, evoking cardiovascular diseases which is the main cause of death of individuals [131]. Left ventricular hypertrophy (LVH) is a strong cardiovascular risk marker in end stage renal disease (ESRD) patients [131]. FokI and BsmI polymorphisms of vitamin D receptor (VDR) gene are regarded as reliable markers of disturbed vitamin D signaling pathway which is also associated with end stage renal disease (ESRD) [304]. Similarly, lower vitamin D status during gestation may be associated with cardiovascular disease risk later in life [305]. Serum concentrations of factors I, II, V, VII, VIII, IX, X, XI, and XII, D-dimers, prothrombin time, INR, aPTT, activity of proteins C and S, antithrombin III, and platelet count are used to evaluate hemostasis in pregnancy [132]. STIM1 plays an essential role in normal cardiac function, mainly homeostasis in the adult heart, which may be important for the regulation of ER and mitochondrial function [133] (Table 2).

Chronic heart failure (CHF) impairs nitric oxide- (NO-) mediated regulation of skeletal muscle O_2_ delivery-utilization matching such that microvascular oxygenation falls faster (i.e., speeds PO2mv kinetics) during increases in metabolic demand. Conversely, exercise training improves (slows) muscle PO2mv kinetics following contractions onset in healthy young individuals via NO-dependent mechanisms [306]. Few other associating markers to assess CVD abnormalities and cardiac functions in pregnant women aortic pulse wave velocity, blood pressure, fasting glucose, HDL, LDL, or C-reactive protein levels are measured. Moreover, during pregnancy higher neonatal 25(OH)D_3_ was associated with higher fasting insulin, triglyceride, and cholesterol (in women) concentrations and with a higher risk of overweight at 35 y of age but not with other adult cardiovascular disease risk factors. Imaging system, using novel enhancer, activated the reporter gene in pressure overload-induced failing hearts [307]. These enhancers are involved in pathological conditions. Noninvasive and quantitative live imaging reveals a potential stress-responsive enhancer in the failing heart [307] (Table 2).

## 10. Anatomical Markers

MRI-visible perivascular spaces (PVS) are potential neuroimaging markers of cerebral small vessel disease, but their functional significance and mechanisms remain uncertain [308]. There is an association between PVS and cognitive impairment and other MRI markers of small vessel disease, in a patient cohort of ischaemic stroke/transient ischaemic attack (TIA). PVS do not have an independent association with cognitive impairment in patients with ischaemic stroke or TIA [308]. Hence, due to increasing complexity in electrophysiology (EP) procedures, the use of electroanatomic mapping systems (EAMS) works as a supplement to fluoroscopy that is used in common practice. Similarly, white matter signals fluctuation on T2-weighted BOLD contrast images are associated with aging and cerebral small vessel disease (SVD) [308]. Therefore, for accuracy assessment of catheter guidance technology in electrophysiology procedures a new 3D-based fluoroscopy navigation system is developed for electroanatomic mapping [74]. NAWM (normal appearing white matter) was found to have graded increases in cardiac pulsations due to age and SVD, independently. Cardiac pulsatility in resting BOLD data may provide a complementary dynamic measure of integrity to add to static FLAIR anatomical images [75] (Table 2).

Clinically, the sight-threatening complications of diabetes are diagnosed and treated based on visible retinal lesions (e.g., dot-blot hemorrhages or retinal neovascularization). These are also indicators of CV diseases in most old age cases, because increasing sugar level affects eye sight and on the other side it also enhances cardiovascular diseases mainly HF [76]. Thus, there remains an urgent need for imaging biomarkers that are abnormal before retinal lesions are visibly apparent and are responsive to treatment. However, MRI biomarkers are used for evaluation of treatment efficacy in preclinical diabetic retinopathy [76]. Similarly, there are diabetes-predictive amino acid score (DM-AA score) that predicts development of CVD and its functional consequences. More often, branched-chain and aromatic amino acids act as novel markers of CVD development and an early link between diabetes and CVD susceptibility can be established [77]. Therefore, mbDPN enlargement, obscurity, surface irregularity, and heterogeneity in echo can serve as the markers indicating nerve ending problems in the diabetic feet of patients under ultrasound interrogation [170] (Table 2).

Cardiac adiposity is associated with coronary artery disease (CAD), obesity, type 2 diabetes, metabolic syndrome, nonalcoholic fatty liver disease, and chronic kidney disease, as well as with CVD risk factors such as lipids, hypertension, obesity markers, and carotid atherosclerosis [78]. It occurs due to lifestyle and CVD drugs on cardiac adipose tissue. However, epicardial adipose tissue exerts cardioprotective properties, but in cases of pathological enlargement, epicardial fat can lead to myocardial inflammation and dysfunction as well as left ventricular hypertrophy and coronary artery disease (CAD) due to paracrine actions that include increased production of reactive oxygen species and atherogenic and inflammatory cytokines [78]. Infantile hemangioma had the classical architecture of capillaries [79]. Therefore, automatic quantification and characterization of coronary atherosclerosis are being done by using computed tomography coronary angiograph, it also shows cross-correlation with intravascular ultrasound virtual histology [309]. Moreover, both plaque volumes and plaque types indicate coronary artery disorders [309]. Small vessel disease (mainly hypertensive arteriopathy and cerebral amyloid angiopathy (CAA)) is an important cause of spontaneous intracerebral haemorrhage (ICH) [195]. It is caused due to enlarged perivascular spaces (EPVS) which are promising neuroimaging marker of small vessel disease. By contrast, basal ganglia EPVS severity is associated with markers of hypertensive arteriopathy [195], atrial fibrillation (AF). Moreover, persistent and long-standing persistent AF may result in electroanatomical changes in the left atrium and deposition of fibrous tissue. It needs AF remodeling for better treatment and detection [310]. Atrial fibrosis occurs due to complex interactions among several cellular and neurohumoral mediators. Hence, atrial substrate modification may increase the risk of thromboembolic complications, including stroke [311] (Table 2).

Adipose tissue is a heterogeneous organ with remarkable variations in fat cell metabolism depending on the anatomical location. However, the pattern and distribution of bioactive lipid mediators between different fat depots and their relationships have important role in formation of complex diseases. However, by using LC-MS/MS-based metabolomics-lipidomics, a range of specialized proresolving mediators (SPM) including resolvin (Rv) D1, RvD2, protectin (PD) 1, lipoxin (LX) A4, and the monohydroxy biosynthetic pathway markers of RvD1 and PD1 (17-HDHA), RvE1 (18-HEPE), and maresin 1 (14-HDHA) have been characterized in human subcutaneous (SC) adipose tissues. The “classic” eicosanoids prostaglandin (PG) E_2_, PGD_2_, PGF2*α*, leukotriene (LT) B_4_, 5-hydroxyeicosatetraenoic acid (5-HETE), 12-HETE, and 15-HETE were also identified in SC fat. SC fat from patients with peripheral vascular disease (PVD) exhibited a marked deficit in PD1 and 17-HDHA levels. In addition, augmented levels of eicosanoids and SPM were observed in SC fat surrounding foot wounds. However, SC PGF2*α* profile, body mass index (BMI), and PGE_2_ indicate fat related disorders. In adipose tissue, mediators RvD2 and LXA_4_ were identified in lower levels than the proinflammatory LTB_4_. Further, diverse distributions of bioactive lipid mediators depend on the localization of human fat depots that uncover a specific SPM pattern closely associated with PVD (Table 2).

Histologically specialised cardiomyocytes are responsible for initiation and propagation of the cardiac impulse. If any disturbance occurs in paranodal area of the terminal crest, and the atrioventricular ring tissues, sinus, and atrioventricular nodes, it provides insights into exploring cardiac implications in CVD patients mainly related to outflow tract tachycardias [135]. The upregulation of galectin-3 occurs in in hypertrophied hearts and the development of fibrosis. Hence, increased galectin-3 levels are associated with poor long-term survival in end-stage heart failure (HF) [136]. Therefore, a relationship between plasma galectin-3 levels and the myocardial tissue expression of galectin-3 is reported in patients with end-stage HF [136]. Plasma galectin-3 levels correlate with the ejection fraction and are elevated in patients with HF. However, the myocardial expression of galectin-3 does not correlate with the ventricular ejection fraction [136] (Table 2).

### 10.1. Therapeutic Biomarkers

Several novel biomarkers of different pathophysiologic pathways as predictors of cardiovascular pathogenesis, disease, and mortality risks are known. Most of these biomarkers are used for identifying cardiovascular heart disease, stroke, and congestive heart failure. However, for finding clinical outcomes all factors such as age of patients, race, sex, education, study year, smoking, abdominal obesity, diabetes, serum total cholesterol, systolic blood pressure, and previous hospitalization are counted for CVD analysis. cTnI is a key biomarker used in hs-cTnI (troponin I) assay, associated with increased cardiovascular death in a community sample when evaluated in a multiple biomarker analysis [70]. Moreover, an increased level of cardiac troponin I is associated with high odds of cardiovascular death [70]. Similarly, NT-proBNP and sST2 are promising biomarkers for identifying patients with little potential to gain significant survival benefit from ICD therapy [156]. Implantable cardioverter defibrillator (ICD) therapy improves survival in patients at high sudden cardiac death (SCD) risk [156] (Table 2).

CVD is also associated with thyroid abnormalities that can be reversed with administration of thyroid replacement therapy. Hence, to find lipid abnormalities measurement of thyroid-stimulating hormone level is also important for initial assessment of patients with dyslipidemia. Similarly, treatment of the underlying cause of nephritic syndrome is accompanied by improvement of lipid levels. Use of standard lipid-lowering therapies can be effective, although patients must be carefully monitored for adverse effects. Therefore, reversing or preventing systemic oxidative damage in experimental and natural aging can assist in finding primary target for the development of effective therapeutic strategies to prevent or treat age-related vascular disorders [157]. Use of endocannabinoid receptor antagonists results in reduction in weight and in waist circumference, in association with lowering triglycerides and raising HDL cholesterol. Adiponectin has antiatherosclerotic properties and is also produced in the local coronary circulation [158] (Table 2).

ECS (endocannabinoid system) plays important role in modulating the function of mitochondria that has a pivotal role in maintaining cellular and systemic energy homeostasis, in large part due to their ability to tightly coordinate glucose and lipid utilization [312]. However, mitochondrial dysfunction is often associated with peripheral insulin resistance and glucose intolerance, as well as the manifestation of excess lipid accumulation in the obese state. ECS may impact upon mitochondrial abundance and/or oxidative capacity and obesity-induced perturbations in metabolic function. It has potential implications in pathogenesis of metabolic disorders and in developing therapies targeting the ECS [312]. Abdominal aortic aneurysm (AAA) disease diagnosis needs development of multiple high-resolution molecular imaging modalities capable of tracking disease progression, quantifying the role of inflammation, and evaluating the effects of potential therapeutics [68].* In vivo* molecular imaging provides molecular and cellular information that can be used for emerging therapeutic biomarkers to study AAA disease prevalence [68].

In addition, hormone therapy (HT) is used in the endothelial function of 46,XY disorders of sexual development (DSD) patients with female phenotype. HT was found to improve biochemical and ultrasound markers of endothelial function in 46,XY DSD patients with female phenotype [159]. For this purpose oral doses of 17*β*-estradiol/1 mg norethisterone acetate are prescribed for daily consumption. It balance the lipid profile, mainly total cholesterol (TC), LDL, HDL, triglycerides (TG), and Atherogenic Index of Plasma (AIP), as well as levels of VE-cadherin, E-selectin, thrombomodulin, and vWf. Metabolic disorders, including obesity and insulin resistance, have their basis in dysregulated lipid metabolism and low-grade inflammation [159]. More often, lipase-related genes were found associated with obesity and secreted phospholipase A_2_s (sPLA_2_s), PLA2G5 and PLA2G2E, were robustly induced in adipocytes of obese mice [160]. PLA2G2E altered minor lipoprotein phospholipids, phosphatidylserine and phosphatidylethanolamine, and moderately facilitated lipid accumulation in adipose tissue and liver. Collectively, the identification of “metabolic sPLA_2_s” adds this gene family to a growing list of lipolytic enzymes that act as metabolic coordinators [160]. As a flippase, ATP13A2 may transport an organic molecule, such as a lipid or a peptide, from one membrane leaflet to the other. A flippase might control local lipid dynamics during vesicle formation and membrane fusion events [161] (Table 2).

There is a well-defined association of dietary, circulating, and supplement fatty acids with coronary risk [162]. Moreover, relative risks for coronary disease can be predicted by using consumption index of various fatty acids (CI, 0.69 to 1.36) for *α*-linolenic, 0.94 (CI, 0.86 to 1.03) for long-chain *ω*-3 polyunsaturated, and 0.89 (CI, 0.71 to 1.12) for *ω*-6 polyunsaturated fatty acid supplementations. Therefore, patient should be encouraged to have high consumption of polyunsaturated fatty acids and low consumption of total saturated fats. Thus, normal physiological level of various fatty acids also works as therapeutic biomarker in coronary heart diseases [163]. A high consumption of omega-3 long-chain polyunsaturated fatty acids, and particularly docosahexaenoic acid (DHA), has been suggested to reduce the risk of cardiovascular disease (CVD) and acts as a therapeutic marker [163]. Similarly, Docosahexaenoic acid supplementation in the diet increases vascular function and lowers down risk factors for cardiovascular disease [163]. A high consumption of omega-3 long-chain polyunsaturated fatty acids, and particularly docosahexaenoic acid (DHA), successfully reduces the risk of cardiovascular disease (CVD) [163]. Thus, DHA supplementation in the diet may have benefits for primary prevention and secondary prevention of CVDs and improves endothelial functions. Nevertheless, lower triglyceride concentrations with DHA supplementation also benefit in the prevention of CVD. Omega-3 Index is emerging as a risk factor for fatal and nonfatal cardiovascular events [164]. It has been defined as eicosapentaenoic plus docosahexaenoic acids in erythrocytes [164]. Omega-3 Index is used as a therapeutic marker [18] (Table 2).

### 10.2. Omega-3 Index as a Risk Factor

There is a strong association of dietary, circulating, and supplement fatty acids with coronary risks [163]. The Omega-3 Index (eicosapentaenoic acid + docosahexaenoic acid) content in red blood cell membranes is well-confirmed novel risk marker for cardiac death [137]. More often, Omega-3 Index and prognosis are used in finding acute coronary chest pain with a low dietary intake of omega-3 fatty acids in patients [137]. It is also used to predict all-cause mortality due to sudden cardiac death following hospitalization with an acute coronary syndrome (ACS). More specifically, arachidonic acid (AA) is used in risk assessment [137] in a population with a low intake of fish and fish oils, but adjusted Omega-3 Index did not predict fatal events following hospitalization in patients with acute chest pain and suspected ACS [137]. Various fatty acids also work as biomarkers in coronary heart diseases. Their level shows effects of age, sex, body mass index, and apolipoprotein E (ApoE) genotype on cardiovascular biomarker response to an n-3 polyunsaturated fatty acid supplementation [138] (Table 2). These factors contribute to the interindividual variability observed in the metabolic response to an n-3 PUFA supplementation [138]. In addition, apolipoprotein E (ApoE) genotype is associated with the metabolic response to an n-3 polyunsaturated fatty acid (PUFA) supplementation [138]. Moreover, n-3 PUFA supplementation significantly decreases plasma triglyceride levels (*P* = 0.0002) with an increase in fasting glucose (FG) levels (*P* = 0.02). Similarly, plasma phospholipid and dietary alpha linolenic acid are associated with incident atrial fibrillation in older adults [139]. Moreover, a relationship of *α*-linolenic acid (ALA 18:3n-3) is confirmed with an intermediate-chain essential n-3 polyunsaturated fatty acid derived from plants and vegetable oils, with incident atrial fibrillation (AF). Both plasma phospholipid and dietary ALA act as biomarker and this relationship is affected by age, sex, race, clinic, education, smoking, alcohol, body mass index, waist circumference, diabetes, heart failure, stroke, treated hypertension, and physical activity. Similarly, n-3 LC-PUFA-supplemented yoghurt effects circulating eicosanoids and cardiovascular risk factors [313]. Moreover, an increase of n-3 LC-PUFA in RBC and plasma lipids due to intake of n-3 LC-PUFA enriched yoghurt resulted in a reduction of cardiovascular risk factors and inflammatory mediators. Hence, daily consumption of n-3 PUFA enriched yoghurt can become an effective way of supplementing the daily diet and improving cardiovascular health [313]. Mainly, omega-3 polyunsaturated fatty acids (n-3 PUFA) occur in marine n-3 fatty acids and natural FA improves cardiovascular health [140]. In particular, marine n-3 PUFA such as eicosapentaenoic acid (EPA) and docosahexaenoic acid (DHA) have been shown to possess cardiovascular protective qualities. Due to the originality of the index, additional evidence is also required to assess and predict CVD [140]. There is strong evidence that the pharmaceutical grade n-3 fatty acid drug Lovaza, (previously Omacor), is effective in reducing triglyceride levels in humans [140]. The Omega-3 Index is used as a good indicator or risk factor for cardiovascular diseases [164]. It is a promising novel biomarker for assessing long-term EPA + DHA status in humans [140]. Similarly, fatty acid lipophilic index is also used as a novel marker to predict risk of CHD because individual FA levels are based on FA affinity and fluidity in relation to CHD risk [314].

Moreover, plasma levels of total trans-FA, long-chain n-3 FA, and polyunsaturated to saturated fat ratio can prepare an adverse profile of cardiovascular risk markers and increased risk of CHD in men [141]. Higher plasma n-3 polyunsaturated fatty acids (PUFA) have been associated with a lower risk of age related cognitive decline and with beneficially affecting cardiometabolic risk factors. A relationship exists between metabolic disorders such as diabetes type 2 and cognitive decline [141]. Fish oil containing n-3 PUFA (3 g daily) if consumed for 5 weeks shows improvement in cognitive performance in healthy subjects. In addition, inverse relations are also obtained between cardiometabolic risk factors and cognitive performance, which indicate a potential of dietary prevention strategies to delay onset of metabolic disorders and associated cognitive decline [141]. There is an association between the n-3 fatty acid *α*-linolenic acid (ALA) and the incidence of congestive heart failure (CHF). Both plasma phospholipid concentrations and dietary consumption of ALA are associated with incident CHF [184]. These prevent CVD risks and available fatty acid measurements can be included in biomarker analyses. Omega-3 Index is emerging as a risk factor for fatal and nonfatal cardiovascular events [164]. It is also defined as eicosapentaenoic plus docosahexaenoic acids level in erythrocytes [164]. Thus, the Omega-3 Index fulfills important criteria for novel biomarkers and its precision value shows an important role in cardiology and probably expands its application to other areas, like psychiatry and pregnancy [164]. Omega-3 index is good predictor of acute myocardial infarction [315] (Table 2).

However, dietary characteristics associated with low omega-3 levels with acute myocardial infarction (AMI) need more investigations to find relationship between risk and probable highly needful omega-3 fatty acid consumption from treatment point of view. As in studies fish intake, older age, race other than white, and omega-3 supplementation were found to be independently associated with a higher Omega-3 Index, whereas frequent fast food intake, smoking, and diabetes mellitus were found to be associated with a lower Omega-3 Index. Potentially modifiable factors, such as patient-reported fast food intake, fish intake, and smoking, are independently associated with the Omega-3 Index in patients with AMI. These characteristics may be useful to identify patients who would benefit the most from omega-3 supplementation and lifestyle modification [315]. The Omega-3 Index is expressed as a percentage of total fatty acids, mainly EPA and DHA in erythrocyte membranes. Hence, it acts as both risk marker and risk factor for CHD death [316] and determinants of the Omega-3 Index are used in increased risk for CHD [316]. Inversely low Omega-3 Index is associated with cardiovascular risk factors [142] (Table 2).

Major depressive disorder is associated with cardiovascular risk factors and low Omega-3 Index [142]. Omega-3 fatty acids have been suggested as disease modulators for both CVD and MDD [142]. Cardiovascular disease (CVD) and major depressive disorder (MDD) are frequent worldwide and have a high comorbidity rate. Several conventional risk factors such as high triglyceride (mean, 152 mg/dL versus 100 mg/dL; *P* < 0.001) and fasting glucose (mean, 96 mg/dL versus 87 mg/dL; *P* = 0.005) values as well as greater waist circumference (mean, 97 cm versus 87 cm; *P* = 0.019) and higher body mass index (calculated as kg/m^2^; mean, 26 versus 24; *P* = 0.011) were found prevalent in MDD patients in comparison with undiseased controls. The Omega-3 Index (mean, 3.9% versus 5.1%; *P* < 0.001) and individual omega-3 fatty acids were significantly lower in MDD patients [142]. But an Omega-3 Index <4% was found to be associated with high concentrations of the proinflammatory cytokine interleukin-6 (*χ* ^2^ = 7.8, *P* = 0.02). Therefore, conventional cardiovascular risk factors, the Omega-3 Index, and interleukin-6 levels indicate an elevated cardiovascular risk profile in MDD patients currently free of CVD [142]. No doubt, Omega-3 Index has greater clinical utility for therapeutic intervention [317]. Moreover, Omega-3 Index functions as an actual risk factor that plays a pathophysiologic role in the disease. Its optimal levels appear to be 8% or greater. The Omega-3 Index both acts as a risk biomarker and as a risk factor [317] (Table 2).

Omega-3 fatty acid supplements improve the cardiovascular risk profile of subjects with metabolic syndrome, including markers of inflammation and autoimmunity [143]. It shows effects on weight, systolic blood pressure, lipid profile, and markers of inflammation and autoimmunity [143]. Fish oil contains high concentrations of omega-3 fatty acids which shows anti-inflammatory properties. It appears that omega-3 direct treatment with omega-3 supplements is associated with a significant fall in body weight (*P* < 0.05), systolic blood pressures (*P* < 0.05), serum low-density lipoprotein cholesterol (*P* < 0.05), and total cholesterol (*P* < 0.05), triglycerides (*P* < 0.05), high-sensitivity C-reactive protein (hs-CRP) (*P* < 0.01), and Hsp27 antibody titers (*P* < 0.05). No significant changes are observed in the control group. Blood levels of omega-3 fatty acids reflect the interplay of metabolism and the intake of omega-3-rich foods (oily fish) [317]. These standards include consistency, strength of association, biological plausibility, coherence, dose-response relationship, clinical utility, cost effectiveness, and prospective validation.

A diet based on high-heat-treated foods promotes risk factors for diabetes mellitus and cardiovascular diseases [318]. Replacing high-heat-treatment techniques by mild cooking techniques may help to positively modulate biomarkers associated with an increased risk of diabetes mellitus and cardiovascular diseases. Long-term lifestyle shows positive impact of change on erythrocyte fatty acid profile after acute coronary syndromes [319]. Moreover, dietary requirements and lifestyle advice, plasmatic lipids, and the fatty acid composition of erythrocyte membrane phospholipids also indicate acute coronary syndromes. Among dietary factors, n-3 polyunsaturated fatty acids reduce mortality from cardiovascular diseases [319]. The positive impact was seen in the blood lipid and erythrocyte fatty acid levels. It also reduces plasma low-density lipoprotein cholesterol and triglyceride concentrations and improves n-3 polyunsaturated fatty acid percentages in phospholipids. Hence, global score, lipid variables, and the nature of the polyunsaturated fatty acids in erythrocyte phospholipids help to evaluate patients with high coronary artery disease risk and possible benefits of long-term dietary and lifestyle advice [319] (Table 2).

## 11. Genetic Markers

Cardiovascular disease (CVD) is a leading cause of mortality and morbidity throughout the world. Efforts have been made for finding reasons and therapeutics of lipid related morbidities. For wider exploration of risks, large genomic studies (GWASs) have been done around the world in various population groups as well as in animal models. In addition, for finding solutions and proper diagnostics, a number of common genetic variants with modest effects on coronary artery disease, myocardial infarction, stroke, and dilated cardiomyopathy have been identified and important modifiable risk factors of CVD are established with a large number of predominantly common variant associations, for example, with blood pressure and blood lipid levels. In each case, despite the large numbers of loci identified, only a small proportion of the phenotypic variance is obtained. There is a possibility that few rare variants with large effects may account for cardiovascular risks which may be driven by individual rare mutations. Therefore, identification of rare variants responsible for monogenic disease may also provide further insight into occurrence and progression biological disease mechanisms [144] (Table 2).

Similarly, mutations occurred in low LDL cholesterol and ApoB genes have provided insight into lipid metabolism, disease associations, and the basis for drug development to lower down LDL cholesterol in patients [145]. Several mutations in the ApoB, proprotein convertase subtilisin/kexin type 9 (PCSK9), and MTP genes result in low or absent levels of ApoB and LDL cholesterol in plasma, which cause familial hypobetalipoproteinemia and abetalipoproteinemia. Mutations in the ANGPTL3 gene cause familial combined hypolipidemia [145]. ANGPTL3 mutations cause low levels of LDL cholesterol and low HDL cholesterol in compound heterozygotes and homozygous individuals, decrease reverse cholesterol transport, and lower glucose levels. A loss-of-function mutation in PCSK9 causes familial hypobetalipoproteinemia, which appears to lower risk for coronary artery disease and has no adverse sequelae. Therefore, early diagnosis and treatment are necessary to prevent adverse sequelae from familial hypobetalipoproteinemia and abetalipoproteinemia [145]. LDL cholesterol (LDLc) uptake by LDLR is regulated at the transcriptional level by the cleavage-dependent activation of membrane-associated sterol response element-binding protein (SREBP-2). PP2A activity regulates cholesterol homeostasis and LDLc uptake [146]. PP2A activity is also required for SREBP-2 DNA binding. Activated SREBP-2 translocates to the nucleus, where it binds to an LDLR (low-density lipoprotein receptor) promoter sterol response element (SRE), increasing LDLR gene expression and LDLc uptake [146]. In response to cholesterol depletion, PP2A directly interacts with SREBP-2 and alters its phosphorylation state and causes an increase in SREBP-2 binding to an LDLR SRE site. Thus, increased binding resulted in induced LDLR gene expression and increased LDL uptake. No doubt, PP2A activity regulates cholesterol homeostasis and LDLc uptake and indicates abnormality if it is disturbed [146] (Table 2).

MetS has become a worldwide epidemic that directly increases the risk of cardiovascular diseases and type 2 diabetes mellitus. The metabolic syndrome (MetS) is a polygenic multifactorial metabolic disorder with strong socioeconomic influence. Moreover, analysis of polymorphism of genes is associated with the development of coronary heart disease (CHD). It also reveals frequency distribution of genotypes and alleles that depends on the ethnic characteristics of the populations [147]. Best example of gene polymorphisms is apolipoprotein genes such as ApoB, ApoC111, ApoE, X2 of ApoB, and S2 of ApoCIII which find association with alcoholism and cardiovascular disorders. Apolipoprotein genes are proved to be good genetic markers of hypertriglyceridemia [147]. The human ApoE gene, coding apolipoprotein E, has three common polymorphisms in human population: e2, e3, and e4, which are proved to be associated with impaired lipid metabolism [148]. In 3T3-L1 cells, FKBP51 is a prominent marker of the differentiated state. JAZF1 plays an important role in lipid metabolism and may thus provide a potential tool for the treatment of obesity and lipid metabolism disorders among other diseases [320]. MicroRNAs are proved noble diagnostic and prognostic biomarkers in atherosclerosis [149, 321] and CAD pathogenesis [150] (Table 2).

Mediator (MED) complex plays key roles in eukaryotic gene transcription and have direct role in CVD initiation and progression. MED involvement in these pathologies is correlated with missense mutations in MED13L gene with transposition of the great arteries [150]. Nowadays, also MED13 and MED15 have been associated with human congenital heart diseases and others could be added, like MED12 that is involved in early mouse development and heart formation [150]. Interestingly, a missense mutation in MED30 gene causes a progressive cardiomyopathy in homozygous mice suggesting a potential role for this subunit also in human CVDs [153]. Moreover, several subunits like MED1, MED13, MED14, MED15, MED23, MED25, and CDK8 exert important roles in glucose and lipid metabolism. Although these evidences derive from* in vitro* and animal model studies, they indicate that their deregulation may have a significant role in human CVD-related metabolic disorders [150]. MED can work as novel biomarkers to be used in combination with imaging techniques for early diagnosis and to develop targets for novel therapeutic approaches [150] (Table 2).

## 12. Future Biomarkers of CVD Risks

### 12.1. Lipid Metabolomics

**Figure 5:**
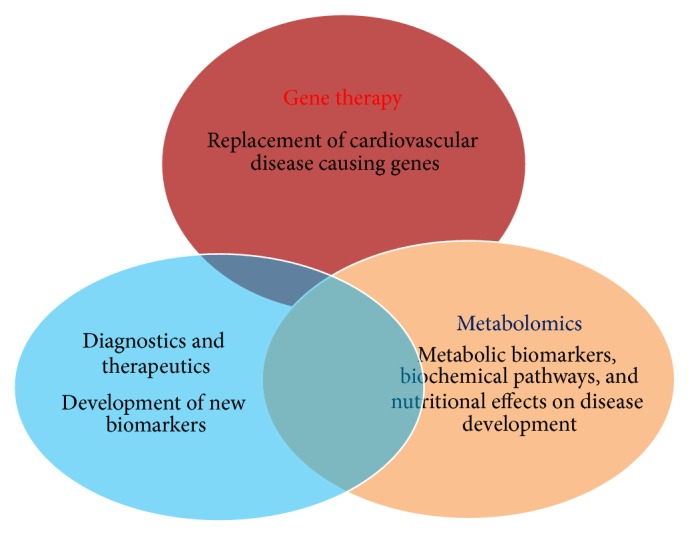
Showing essentiality for integration of diagnostics, therapeutics, metabolomics, and gene therapy for most possible treatment of cardiovascular disease.

Metabolic profiling, or metabolomics, has developed into a mature science in recent years. It has major applications in the study of metabolic disorders. Metabolomics includes study design and data analysis in diabetes, CVDs, HF, and obesity research. Recent advances made in metabolomics are used in identification of markers for altered metabolic pathways, biomarker discovery, and therapeutic challenge, metabolic markers of drug efficacy, and off-target effects. The role of genetic variance and intermediate metabolic phenotypes and its relevance to diabetes and lipid disorders are also addressed [152]. Hence, mining of the genome for lipid genes can clear presence of triglycerides in chylomicrons and its packaging and repackaging (in very low-density lipoproteins; VLDL). When hydrolyzed, remnant and low-density lipoproteins (LDL) are cleared from the circulation [153]. Gene discoveries have also provided insights into high-density lipoprotein (HDLp) biogenesis and remodeling [153]. More specifically, after advent of genomics main lipid pathways are uncovered and certain modulators or adaptor proteins such as those encoded by LDLRAP1, ApoA5, ANGPLT3/4, and PCSK9 are discovered by genome wide association studies (GWAS). More often, lipid abnormalities and its associated health implications in man have been identified after so many disease responsible genes, its presence on loci, statistical analyses, and functional annotations. These might show large impacts on lipoprotein traits as gene products that are already known. But importance of new candidate genes is challenging because these may show very low frequencies of large impact variants in the population [153]. More often, selection may also work at lipoprotein structure level earlier than its functional evolution, because feeding of variations in alpha and beta chains shifts and reshifts amino acids. Hence, there might be some genes which most possibly make such changes in lipoprotein structure. One of the genes showing the strongest evidence of selection, ApoB (apolipoprotein B), encodes the primary lipoprotein component of low-density lipoprotein (LDL). In addition, functional mutations in ApoB may explain how polar bears are able to cope with life-long elevated LDL levels that are associated with high risk of heart disease in humans [154] (Figures 4 and 5). MicroRNAs emerge as biomarkers in distinguishing HFpEF versus HFrEF markers [155] (Table 2).

A consortium of metabolic risk factors accelerates the onset of diabetes, heart disease, stroke, and certain cancers. DNA based diagnostics assists in identification of molecular basis of monogenic dyslipidemias. However, recent reports of the application of whole genome or whole exome sequencing in families with severe dyslipidemias have largely identified genetic variants in known lipid genes. However, high-throughput DNA sequencing in families with previously uncharacterized monogenic dyslipidemias has failed to reveal new genes for regulation of plasma lipids. Therefore, it is not necessary to sequence whole genomes or exomes, but monogenic dyslipidemias could be diagnosed using a more dedicated approach that focuses primarily on genes already known to act within lipoprotein metabolic pathways in most of patients (Figure 5) [151]. Moreover, terminal deoxynucleotidyl transferase dUTP nick end labeling (TUNEL) assay demonstrated coptisine suppressed myocardial apoptosis, which may be related to the upregulation of Bcl-2 protein and inhibition of caspase-3 activation (Table 2).

### 12.2. Integration of Traditional Risk Factors

Noninvasive arterial wall imaging is used to establish reasons of subclinical atherosclerosis. These techniques are used to predict risk and to evaluate the potential efficacy of therapeutic agents. Similarly, role of genetics in cardiovascular disease become more apparent and genetic profiling of large population cohorts is used to identify new markers of risk and novel targets for therapeutic modification. No doubt, potential of genotyping to tailor therapy in individuals remains a hypothesis that needs further exploration. Similarly, body mass index simply predicts presence of metabolic syndrome (MetS) and cardiovascular disease (CVD) risks in elderly population [322] (Figure 5). Obesity is often associated with abnormalities in cardiac morphology and function. Obesity-related cardiomyopathy is caused by impaired cardiac energetics that could be detected by measuring oxygen consumption in isolated cardiac mitochondria. The expression of proteins involved in energy metabolism, and markers of oxidative stress and calcium homeostasis. Similarly,* in vivo* cardiac phosphocreatine-to-ATP ratio and* ex vivo* oxygen consumption in isolated cardiac mitochondria is an indicator of diastolic dysfunction or impaired cardiac energetics. More often, myocardial lipid accumulation was associated with oxidative stress and fibrosis, but not apoptosis. Hence, for better prediction of CVD a cohort or integration of both traditional risk factors with both emerging biomarkers is highly needed (Table 2).

Biochemical markers and noninvasive methods such as the electrocardiogram and echocardiography have a role in diagnosis of idiopathic inflammatory myopathies in man [323] (Figure 5). Furthermore, HFD feeding strongly reduced the phosphorylation of phospholamban, a prominent regulator of cardiac calcium homeostasis and contractility. HFD-induced early stage cardiomyopathy is associated with lipotoxicity-associated oxidative stress, fibrosis, and disturbed calcium homeostasis, rather than impaired cardiac energetic status in mice [324]. But secondary factors such as low-density lipoprotein cholesterol are potentially ambiguous and did not clear CVDs at molecular level [288]. Prevention of venous thromboembolism and pulmonary thromboembolism (PTE) needs anticoagulant therapy because of increased mortality [325]. Composite hemangioendothelioma (CHE) is an intermediate-grade vascular tumor which is a common vascular tumors exhibiting composite variants [326]. Cerebral microbleeds (CMBs) are associated with increased risk of stroke and poor cognition. Vascular risk factors and medications used for stroke prevention may increase the risk of CMB. However, prevalence of CMB is associated with risk factors which are also associated with markers of hypertensive vasculopathy, low cholesterol, and APOE *ε*4 with lobar CMB [327]. Its potential adverse effect is improved by statin in combination of other cohorts [327]. Corticosteroids and immunosuppressive therapy were also found effective in the control of disease activity. In additions, regular surveillance by clinical instruments and imaging is warranted to estimate the disease progression and morbidity status [328]. Chest pain and elevated troponins in a patient with prior coronary artery disease are also noted as an important diagnostic approach [329]. In addition, impairment in cardiac fibroblasts inhibits proper heart function and needs cell-specific markers [330]. However, an unbiased comparative gene expression profile of the cardiac fibroblast pool, identification and characterization of key genes in cardiac fibroblast function, and their contribution to myocardial development and regeneration is highly required [330]. It is true that cardiogenic genes expressed in cardiac fibroblasts contribute to heart development and repair [330] (Table 2) (Figure 5).

Abdominal obesity is associated with a dramatic increase in cardiovascular risk. This is likely to result from the interaction of metabolic risk factors (hypertriglyceridemia, low HDL cholesterol, small dense LDL, insulin resistance, hypertension, and inflammation). It is also apparent that abdominal adipose cells elaborate a range of factors, termed as adipocytokines, and these factors show effects on metabolic regulation and directly on the artery wall. Moreover, few important markers such as elevated levels of leptin and resistin are both associated with an adverse cardiovascular outcome related to adipocytokines that may predict cardiovascular risk. In contrast, adiponectin, which appears to have a favorable influence on a number of pathological events in the artery wall, predicts relative protection from cardiovascular disease (Table 2).

**Figure 6:**
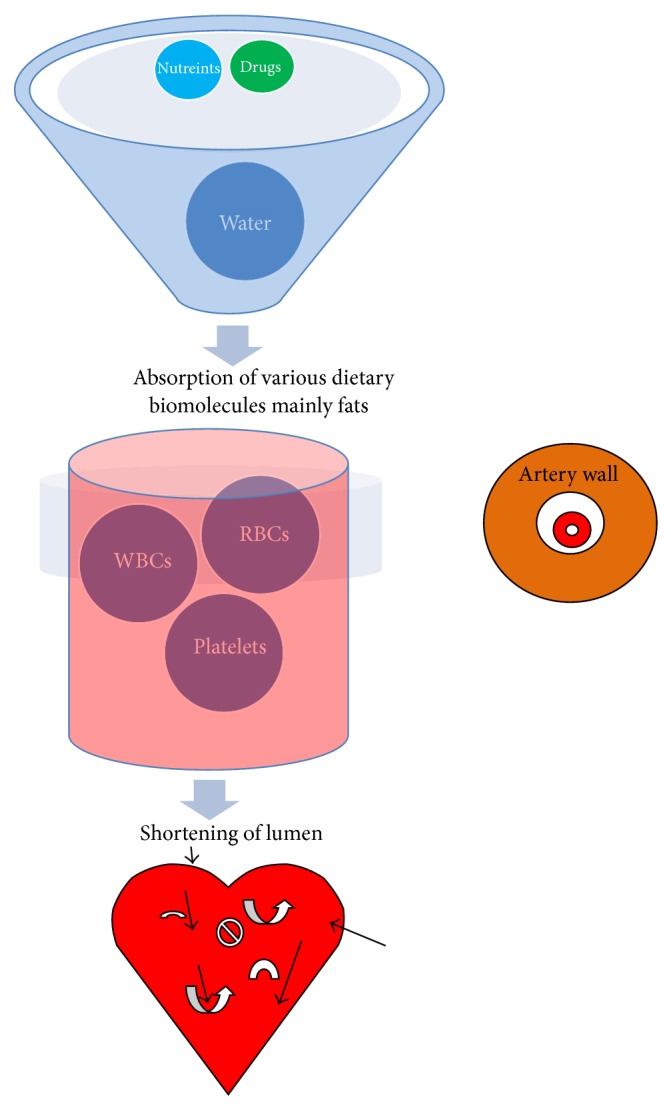
Shortening of artery wall due to deposition of cholesterol and its binding lipoproteins.

Thrombin has been reported to play a pivotal role in the initiation of angiogenesis by indirectly regulating and organizing a network of angiogenic molecules. However, intramyocardial thrombin promotes angiogenesis and improves cardiac function in experimental rabbit model of acute myocardial infarction [269]. Thrombus formation within the artery lumen is the pivotal event that promotes luminal compromise and systemic levels of factors are found to be involved in regulation of thrombosis which predict cardiovascular risk. Elevated levels of prothrombotic factors, tissue factor, and von Willebrand factor, as well as reduced levels of protective factors, PAI-1, and thrombomodulin, have been reported in association with a greater risk of cardiovascular dysfunction [269]. Homocysteine is a factor involved in the promotion of both thrombosis and inflammation. Although lowering levels of homocysteine with folic acid has not been demonstrated to be protective in clinical trials, elevated systemic levels do correlate with cardiovascular risk. Platelet-derived microparticles may play an important role in the regulation of thrombotic and inflammatory events [269] (Table 2 and Figure 6).

Ethanolamides of long-chain fatty acids are a class of endogenous lipid mediators generally referred to as N-acylethanolamines (NAEs). NAEs include anti-inflammatory and analgesic palmitoylethanolamide, anorexic oleoylethanolamide, stearoylethanolamide, and the endocannabinoid anandamide. Traumatic brain injury (TBI), associated with a high morbidity and mortality, is a complex process evoking systemic immune responses as well as direct local responses in the brain tissues. TBI induces immediate neuropathologic effects such as cumulative neural damage and degeneration. More often, TBI leads to increased catabolism of phospholipids, resulting in a series of phospholipid breakdown products, some of which have potent biological activity. Ischemia-reperfusion (I/R) injury resulting from stroke leads to metabolic distress, oxidative stress, and neuroinflammation. Therefore, NAEs play very important role in lipid signaling in brain and make multipotential actions on neuronal cell death and neuroinflammatory pathways which can be developed as biomarkers for lipid based disorders in several groups of patients with multiple effects [22] (Table 2).

Similarly, elevated levels of metalloproteinases (MMP) and reduced levels of their tissue inhibitors (TIMPs) have been reported in association with elevated cardiovascular risk in some studies. Favourable effects on these systemic factors have been reported in association with statin therapy. It remains to be determined whether MMP activity would provide effective risk stratification. Proteolytic enzymes like matrix metalloproteinases (MMP) are regulated by a group of endogenous factors/enzymes. These enzymes show breakdown of collagen integrity within the fibrous cap in male and display higher MMP level alteration in comparison to female patients [331]. Trehalase can become a possible marker of intestinal ischemia-reperfusion injury [332] because of its release, binding, and perfusion inside cardiac muscles that display cardiomyopathy disease [100]. Coptisine treatment also attenuated the proinflammatory cytokines including interleukin-1*β* (IL-1*β*), IL-6, and tumor necrosis factor-*α* in heart tissue [100]. By measuring level of these inflammatory molecules, myocardial ischemia/reperfusion injury can be identified in patients [100] (Table 2).

### 12.3. Treatment of Lipid Metabolic Disorders

Berberine is known to improve glucose and lipid metabolism disorders, but it is poorly absorbed into the blood stream from the gut. Berberine acts directly in the terminal ileums and can improve blood glucose levels in diabetic rats. Important mechanisms involved may be in the MAPK and GnRh-Glp-1 pathways in the ileum [333]. Interestingly, regular strawberry consumption augments plasma antioxidant activity and decrease lipid peroxidation. Its juice or extract shows very preventive potential against oxidative stress-dependent disorders. Strawberry consumption exerts effects on the luminol enhanced whole blood chemiluminescence (LBCL) reflecting oxidants generation by circulating phagocytes and these phagocytes are important source of oxidants that may contribute to systemic oxidative stress. However, decrease in resting LBCL clear that regular strawberry consumption may suppress baseline formation of oxidants by circulating phagocytes. This may decrease the risk of systemic imbalance between oxidants and antioxidants and become one of the important mechanisms of health-promoting effect of these fruits consumption on cardiac functions [334] (Table 2).

### 12.4. Dietary Control of Cardiovascular Risks

The ketogenic diet (KD) is a broad-spectrum therapy for medically intractable epilepsy and is receiving growing attention as a potential treatment for neurological disorders arising in part from bioenergetic dysregulation. The high fat, low-carbohydrate classic KD, as well as dietary variations such as the medium-chain triglyceride diet, the modified Atkins diet, the low-glycemic index treatment, and caloric restriction, enhances cellular metabolic and mitochondrial function. Similarly, fibrous low calorie fat free diet can improve cellular lipid metabolism and enchance desorption of cholesterol from intracellular pools, because a low fat diet or ketogenic diet restricts glycolysis and increases fatty acid oxidation, resulting in ketosis, replenishment of the TCA cycle (i.e., anaplerosis). It implicates in restoration of neurotransmitter and ion channel function and enhanced mitochondrial respiration. It also restores key signaling pathways that evolved to sense the energetic state of the cell and that help maintain cellular homeostasis. Ketogenic pathways include peroxisome proliferator-activated receptors and AMP-activated kinase [335]. Fish consumption is considered beneficial for health as it decreases cardiovascular disease (CVD) risk through effects on plasma lipids and inflammation. More specifically, salmon protein hydrolysate (SPH) influences lipid metabolism to have antiatherosclerotic and anti-inflammatory properties [336] (Table 2) (Figure 5).

Similarly, dietary long-chain n-3 polyunsaturated fatty acids (LCn-3PUFA) improve endothelial function in medium-large-sized arteries but have effects on small peripheral arteries, responsible for most arterial resistance. Moreover, increased dietary consumption of LCn-3PUFA might be a cost-effective strategy to improve peripheral vasoactivity [222]. But effects of increasing LCn-3PUFA intake with the usual diet on small artery reactive hyperemia index (saRHI) are well noticed. Consumption of functional foods enriched with phytosterols (PSRs) and phytostanols (PSNs) reduces LDLc concentrations by 10% as average. Although recommended as part of any lipid-lowering diet in the first intervention step, PSRs/PSNs maintain their LDL reduction capacity when administered with lipid-lowering drugs; therefore, these are used as an adjuvant to drug therapy. PSR/PSN supplementation shows LDL reducing effects of alone or as an add-on to hypolipidemic drugs interpretation for its clinical use [222]. More specifically, a 5% (w/w) SPH diet reduced atherosclerosis in apoE^−/−^ mice and attenuate risk factors related to atherosclerotic disorders by acting both at vascular and systemic levels and not directly related to changes in plasma lipids or fatty acids [336]. SPH-feeding decreased the plasma concentration of IL-1*β*, IL-6, TNF-*α*, and GM-CSF, whereas plasma cholesterol and triacylglycerols (TAG) were unchanged, accompanied by unchanged mitochondrial fatty acid oxidation and acyl-coenzyme A: cholesterol acyltransferase (ACAT) activity [336]. Lipid mediators, in the inflammatory processes, have potential value of controlling phospholipids metabolism through sPLA_2_ inhibition study [337] (Table 2) (Figure 5).

Neuronal membranes are rich in polyunsaturated fatty acids, which are particularly susceptible to oxidative stress. Moreover, biological roles of products produced by lipid peroxidation can assist in exploration of pathological mechanisms associated with neurological disorders and have clinical applications as biomarkers [338]. Lipid peroxidation biomarkers indicate formation of reactive oxygen species which are measured for evaluating oxidative stress that mainly occurs in neurological disorders, including Alzheimer's disease, Down syndrome, Parkinson's disease, and stroke [338]. Isoproterenol-induced myocardial lipid peroxidation and protein oxidation are also significantly decreased by carnosic acid pretreatment. No doubt, therapeutic application of carnosic acid might be beneficial in treating cardiovascular disorders [338]. Lipid components in biological membranes are essential for maintaining cellular functions. Phosphoinositides, the phosphorylated derivatives of phosphatidylinositol (PI), regulate many critical cell processes involving membrane signaling, trafficking, and reorganization [339]. Multiple metabolic pathways including phosphoinositide kinases and phosphatases and phospholipases tightly control spatiotemporal concentration of membrane phosphoinositides. Metabolizing enzymes responsible for PI 4,5-bisphosphate (PI(4,5)P2) production or degradation play a regulatory role in Toll-like receptor (TLR) signaling and trafficking [339]. These enzymes include PI 4-phosphate 5-kinase, phosphatase and tensin homolog, PI 3-kinase, and phospholipase C. PI(4,5)P2 mediates the interaction with target cytosolic proteins to induce their membrane translocation, regulate vesicular trafficking, and serve as a precursor for other signaling lipids [339]. TLR activation is important for the innate immune response and is implicated in diverse pathophysiological disorders. TLR signaling is controlled by specific interactions with distinct signaling and sorting adaptors. Importantly, TLR signaling machinery is differentially formed depending on a specific membrane compartment during signaling cascades [339] (Figure 5).

### 12.5. How to Decrease the Risk of CVD in All Age Groups

Lowering the blood concentration of low-density lipoprotein (LDL) cholesterol is the primary strategy employed in treating atherosclerotic disorders. However, statins are most commonly prescribed to prevent cardiovascular events in just 30% to 40% of treated patients. Therefore, additional treatment is required for patients in whom statins have been ineffective. Similarly, probucol, a lipid-lowering drug, shows potent antioxidative effects, which when added to treatment with atorvastatin reduces chances of atherosclerosis in animal models. But atherosclerosis is induced by feeding 0.5% cholesterol for 8 weeks in the diet. Atorvastatin decreased the plasma concentration of non-high-density lipoprotein cholesterol (non-HDLc) dose-dependently; atorvastatin 0.003% decreased the plasma concentration of non-HDLc by 25% and the area of atherosclerotic lesions by 21%. Probucol decreased the plasma concentration of non-HDLc to the same extent as atorvastatin (i.e., by 22%) and the area of atherosclerotic lesions by 41%. Probucol with 0.003% atorvastatin decreased the plasma concentration of non-HDLc by 38% and the area of atherosclerotic lesions by 61%. More importantly, coadministration of probucol with atorvastatin did not affect the antioxidative effects of probucol. Probucol has significant add-on antiatherosclerotic effects when combined with atorvastatin treatment [340].

Lipid lowering, particularly with HMG CoA reductase inhibitors (“statins”) reduces the risk of cardiovascular disease. Patients with chronic liver and kidney disease present challenges to the use of lipid medications [341]. There is evidence that most lipid-lowering medications create serious problems if their proper prescription is not followed according to physician. Sometimes these are proved unsafe. In contrast, in chronic kidney disease, dosing of lipid medications may require substantial modification depending on creatinine clearance [341]. There are significant alterations in lipid metabolism in chronic kidney disease with concomitant increases in cardiovascular risk. More specifically, lipid physiology and cardiovascular risk in specific liver and kidney diseases are affected by lipid lowering and the use of statin and nonstatin therapies [341].

Lipolysis regulates energy homeostasis through the hydrolysis of intracellular triglycerides and the release of fatty acids for use as energy substrates or lipid mediators in cellular processes. Genes encoding proteins that regulate energy homeostasis through lipolysis are much likely to play an important role in determining susceptibility to metabolic disorders. These regulate transcription factors responsive to peroxisome-proliferator-activated receptor *γ* (PPAR-*γ*) and show downstream target regulation in adipose tissue in patients with the DD genotype which alter the regulation of pathways influencing adipogenesis, insulin sensitivity, and lipid metabolism. These findings indicate the physiological significance of HSL in adipocyte function and the regulation of systemic lipid and glucose homeostasis and underscore the severe metabolic consequences of impaired lipolysis [342]. N-Palmitoylethanolamide (PEA) is emerging as a novel therapeutic agent in the treatment of neuropathic pain and neurodegenerative diseases. Unfortunately, PEA poorly reaches the central nervous system (CNS), after peripheral administration, since it is inactivated through intracellular hydrolysis by lipid amidases [343].

Familial hypercholesterolemia (FH) is the most common and severe monogenic form of hypercholesterolemia. This is an autosomal codominant disease characterized by an increased plasma low-density lipoprotein (LDL) cholesterol concentration and premature coronary heart disease (CHD). The clinical phenotype depends on the gene involved and severity of mutation (or mutations) present. Patients with homozygous or compound heterozygous FH showed more severe hypercholesterolemia (LDL cholesterol >13 mmol/L) due to a gene dosing effect and without treatment have accelerated atherosclerotic CHD from birth and frequently die of CHD before age of 30. Dyslipidaemia was found to be more prevalent in smokers and alcoholics with diabetes. These persons also face strong coronary heart disease [344]. Therefore, to cure hypercholesterolemia cholesterol-lowering therapies are shown to reduce both mortality and major adverse cardiovascular events in individuals with FH. Lipoprotein apheresis concomitant with lipid-lowering therapy is the treatment of choice for homozygous FH patients [345].

Dyslipoproteinemia is a cardinal feature of the metabolic syndrome that accelerates atherosclerosis. It is characterized by high plasma concentrations of triglyceride-rich and apolipoprotein B- (ApoB-) containing lipoproteins, with depressed high-density lipoprotein (HDL) and increased small dense low-density lipoprotein (LDL) particle concentrations [346]. Dysregulation of lipoprotein metabolism in the metabolic syndrome may be due to a combination of overproduction of very-low density lipoprotein (VLDL) ApoB, decreased catabolism of ApoB-containing particles, and increased catabolism of HDL-ApoAI particles. These abnormalities are due to a global metabolic effect of insulin resistance and visceral obesity [347]. Hence, lifestyle modifications mainly related to dietary restriction and increased exercise and pharmacological treatments may favorably alter lipoprotein transport by decreasing the hepatic secretion of VLDL-ApoB and the catabolism of HDL-ApoAI that increases the clearance of LDL-ApoB [347]. There are several therapies in phase of development for correcting triglyceride-rich lipoprotein and HDL metabolism. However, its clinical efficacy, safety, tolerability, and cost-effectiveness remain to be demonstrated [347].

Homozygous familial hypercholesterolemia (HoFH) is associated with severe hypercholesterolemia and premature cardiovascular morbidity and mortality. Lomitapide reduces production of apolipoprotein B-containing lipoproteins and significantly reduces serum levels of LDL cholesterol [332]. Lomitapide is a novel microsomal triglyceride transfer protein inhibitor that does not depend on the ability to upregulate LDL receptors on the surface of hepatocytes. Patients with HoFH have attenuated responsiveness to lipid lowering therapies such as statins, cholesterol absorption inhibition, and bile acid binding resins because of impaired LDL receptor expression [332]. The most frequent cause of HoFH is loss of function mutations in the gene for the low-density lipoprotein receptor, resulting in reduced clearance of low-density lipoprotein (LDL) cholesterol from the circulation [332].

### 12.6. Daily Physical Exercise

Major cardiovascular disorders occur earlier in life because of dietary problems, lack of exercise, improper medication, and genetic reasons. Nowdays, this has become a common habit of eating spicy food, intaking alcohol, and smoking which inhibit normal metabolic functions and accelerate deformities related to cardiac muscle cell metabolism. Hence, daily exercise, indoor games, and athletics burn extra lipids from the body. It increases oxygen consumption and catabolism of unnecessary fat deposited inside different tissues and from adipocytes. For example, both swimming and soccer training in adolescent correct the lipid-lipoprotein profiles. An exhaustive, aerobics, and exercise cut down cardiovascular risk and enhance antirisk factors. It improves HDL, ApoAI, and ApoB levels and burn bad cholesterol [348]. Apolipoproteins act as mediators of crosstalk between adipose tissue and liver and influence development of obesity and hepatosteatosis mainly nonalcoholic fatty liver disease (NAFLD). However, exchangeable apolipoproteins can be used as therapeutic markers [349]. But there are reports on lipid-lowering therapy in patient which imposes adverse effect on kidney and causes end-stage renal failure that also leads to cardiac failures.

Lipid absorption involves hydrolysis of dietary fat in the lumen of the intestine, followed by the uptake of hydrolyzed products by enterocytes. Lipids are resynthesized in the endoplasmic reticulum and are either secreted with chylomicrons and HDLp or stored as cytoplasmic lipid droplets. Lipids in the form of droplets are hydrolyzed and are secreted at a later time. Secretion of lipids by the chylomicron and HDL pathways are dependent on microsomal triglyceride transfer protein (MTP) and ATP-binding cassette family A protein 1, respectively, and are regulated independently. Gene-ablation studies showed that MTP function and chylomicron assembly are essential for the absorption of triglycerides. Ablation of MTP abolishes triglyceride absorption and results in massive triglyceride accumulation in enterocytes. Although the majority of phospholipid, cholesterol, and vitamin E are absorbed through the chylomicron pathway, a significant amount of these lipids are also absorbed via the HDL pathway. Chylomicron assembly and secretion are increased by the enhanced availability of fatty acids, whereas the HDL pathway is upregulated by liver X receptor agonists. Triglycerides are exclusively transported with chylomicrons and this process is critically dependent on MTP. In addition to chylomicrons, absorption of phospholipids, free cholesterol, retinol, and vitamin E also involves HDLp. These two pathways are complementary and are regulated independently. They may be targeted to lower lipid absorption in order to control hyperlipidemia, obesity, metabolic syndrome, steatosis, insulin resistance, atherosclerosis, and other disorders [350]. Hence, an early diagnosis and treatment are necessary to prevent adverse sequelae from familial hypobetalipoproteinemia and abetalipoproteinemia [145, 351].

GH disorders suggested an impact of IGF1 and IGF-binding protein-3 (IGFBP3) on lipid metabolism [352]. There is cross-sectional and longitudinal associations between IGF1 or IGFBP3 serum levels and lipids (total LDL or HDL cholesterol and triglycerides) which indicate association between the GH/IGF axis and lipid metabolism [353]. Plasma and cardiac resisting levels were also significantly decreased by SQE supplementation. SQE supplementation decreased the accumulation of lipid droplets, inflammatory cell infiltrations, levels of triglycerides, and total lipids in the liver and effectively downregulated expression of sterol regulatory element binding protein-1 (SREBP-1), fatty acid synthetase (FAS), and uncoupling protein-2 (UCP-2). In adipose tissue, mRNA levels of CCAAT/enhancer-binding protein *β* (C/EBP*β*) are suppressed which is a potential treatment for high fat-related disorders. It could also improve lipid profiles, modulating lipid metabolism [352]. Similarly, Niemann-Pick disease type C (NPC) is caused by defects in cholesterol efflux from lysosomes due to mutations in genes coding for NPC1 and NPC2 proteins. It results in massive accumulation of unesterified cholesterol in late endosomes/lysosomes. GST-PFO can be a convenient and reliable probe for revealing cholesterol deposits in cells and can be useful in diagnostics of NPC disease [353]. Hence, there seems an essential need of integration of diagnostics, therapeutics, metabolomics, and gene therapy for most possible treatment of cardiovascular disease (Figure 5).

## 13. Summary

From studies it has been come out that in last two-three decades major adverse cardiovascular events (MACE) have been enormously increased. Every year millions of CVD cases are reported in the hospitals but a large number of people that never reach to hospital and expire as unreported. There is hundreds of lipid, carbohydrate, and protein related cardiac dysfunctions which are associated with CVD disease. These are considered as disease favoring factors and also known as emerging risks factors because they indicate presence of some short of cardiovascular dysfunction. Many of these diseases are detected during rehospitalization of patients due to morbidities like unstable angina, heart failure, nonfatal myocardial infarction, arteriosclerosis, and cardiovascular diseases. Besides risk factors there are some faulty dietary consumption like sugar-rich or fat-rich diets which increase CVD risks manyfold, because sugar-rich foods are inversely associated with 7 unsaturated long-chain fatty acids. These attribute oxidative stress and impose disordered lipid profiles. In addition, an increased use of drugs, beverages, and sex also induce oxidative stress and contribute to CVD risks. Atherosclerosis and diabetes are one of the most common disorders among the elderly which are correlated with these factors. Depression is also found to be associated with the development of atherosclerosis and diabetes. Advanced glycation end products play a pivotal role in atherosclerosis [354]. Similarly, inflammation also plays important roles at all stages of atherosclerosis. Inflammation and endothelial dysfunction have been implicated in the pathogenesis of atherosclerotic vascular disease and cause metabolic disturbances like diabetes, atherosclerosis, or metabolic syndrome. Cerebrovascular disease (CVD) and Alzheimer disease are significant causes of cognitive impairment in the elderly. Similarly, chronic kidney disease (CKD) is also an independent risk factor for coronary artery disease (CAD). Gene disorders cause acute coronary syndrome (ACS) and need one of the most frequent differential diagnoses in emergency medicine.

More often, atherosclerotic peripheral arterial disease (PAD) is one of the most prevalent, morbid, and mortal diseases. Chemokines play important roles in atherosclerotic vascular disease. These have major effects on the initiation and progression of atherosclerosis by controlling the trafficking of inflammatory cells* in vivo* through interaction with their receptors. Long-term intake of long-chain n-3 polyunsaturated fatty acids (n-3 PUFAs), especially eicosapentaenoic acid (EPA), is associated with a low risk for cardiovascular disease. It is well known that people with high levels of body fat remain at higher risk for developing diabetes mellitus, kidney disease, and cardiovascular disorders. Since individuals who are slightly overweight, or even individuals of normal weight, can vary in body fat distribution, their metabolic profiles and the degree of association of these profiles with cardiometabolic risk factors may differ. Moreover, fat distribution might be more of a predictive factor for cardiorenometabolic risk compared to obesity itself, which has led researchers to investigate whether ectopic fat accumulation may partially account for the development of cardiorenometabolic disorders. In addition, visceral obesity, fat accumulation in the liver and muscle, results in intrahepatic and intramuscular lipid storage which is also associated with insulin resistance and adverse metabolic phenotypes. More recently, pericardial fat, perivascular fat, and perirenal fat were found to be associated with coronary atherosclerosis, cardiovascular diseases, and kidney damage, respectively. Thus, regional fat distribution may play a key role in understanding the development of cardiorenometabolic diseases in nonobese people [355]. Hence, fat consumption or extreme calorie consumtion burning by passing through an aerobic exercise provides some relief, defeating drugs. In addition, high fat intake in diet should be avoided by all categories of people because it shows long-term effects and evokes obesity genes.

A large number of novel biomarkers that reflect a broad range of pathological events involved in the progression of atherosclerosis have been reported in association with cardiovascular risk. Moreover, LDL cholesterol level, HDLp and apolipoprotein levels, lipophorins and LTPs ratio, sphingolipids, Omega-3 Index and ST2, immunohistochemical, oxidative stress, inflammatory, anatomical, imaging, genetic markers, and therapeutic biomarkers are proved to be much better for clinical diagnosis of CVDs. Further, assessment of global risk may require integration of multiple biomarkers reflecting the different pathological pathways involved in atherosclerosis (Figure 5). Hence, all risk factors which can assist in incremental risk prediction must be included in diagnosis, to tailor therapy or to monitor the effects of therapy in a cost-effective manner. Each of the pathological pathways involved in the generation and subsequent rupture of atherosclerotic plaque might theoretically reveal systemic markers that may be of utility in risk prediction and in monitoring the response to therapy. Additional markers of oxidative stress may be of high utility if developed. Systemic levels of the MPO products, chlorotyrosine, and nitrotyrosine and use of statin were found to be the best therapeutic biomarkers for best cardioprotective measures. In addition, various metabolites of arachidonic acid can be measured in both blood and urine and have been reported as associated with cardiovascular risks. In the cardiovascular system, miRNAs not only impact on physiological pathways like cardiac development and angiogenesis, but also play an important role in disease mechanisms and progression of myocardial hypertrophy, acute myocardial infarction, heart failure, or arrhythmias. Hence, an association between inflammatory markers and future HF risk in patients with stable CAD needs further explorations for finding possible solution of myocardial infarction (MI) in man. Other inflammatory markers such as interleukin-1 receptor-1 (IL1R1) and its ligand, IL1*β*, are upregulated in cardiovascular disease, obesity, and infection. Apolipoproteins are very heterogeneous protein family, implicated in plasma lipoprotein structural stabilization, lipid metabolism, inflammation, or immunity. However, by measuring serum and plasma lipoproteins, their components and lipid profile of patients may not only predict occurrence of CVDs based on lipoprotein roles in atherogenesis but also help in development of new therapeutic strategies for the treatment of lipoprotein-associated disorders. Thus, improving the quality of HDL may represent a better therapeutic target than simply raising the HDL level and assessment of HDL function may prove informative in refining our understanding of HDL-mediated atheroprotection. Metabolomics may reveal novel metabolic biomarkers of dietary intake and provide insight into biochemical pathways underlying nutritional effects on disease development.

Lipid disorders are also the risk factors for CVDs. Therefore, an earlier diagnosis and treatment are necessary to prevent different types of lipid abnormalities and to predict emerging risks of various cardiovascular diseases and disorders (Figure 5). There is a need to prepare a detailed metabolic calendar for daily dietary use to minimize the CVD risks in patients. More specifically obesity-induced perturbations in metabolic function should be checked from time to time. Though dietary lipid abnormalities can be reversed by changing the food habits, exercise, and use of fat mobilizing drugs and replacement therapy, genetic abnormalities are not possible to restore them immediately and need effective control measures with proper clinical care. It is an important issue which is directly related to public health. Hence, combined efforts are needed to develop and explore new risk biomarkers for an accurate and proper disease diagnosis. In addition, more sophisticated therapeutic markers are also needed for achieving good therapeutic targets. Conclusively this huge target could not be achieved without making integrating efforts made by biochemists, immunologists, molecular biologists to unfold the mystery of CVDs, its more accurate diagnosis, and therapeutics as well.

## Figures and Tables

**Table 1:** Important emerging risk biomarkers in cardiovascular disease and disorders.

Name of disease	Effects	Risk score	Biomarker	References
Homozygous familial hypercholesterolemia	Premature cardiovascular morbidity and mortality	10–20%	Total cholesterol, LDL cholesterol	

Hypertriglyceridemia/ hypertriglyceridemia	Elevated levels of Lp(a)	20%	Lipid profile	

Chronic kidney disease	Elevated levels of Lp(a)	10%	Lipid profile	

Cholelithiasis	Gall stone formation due to cholesterol and salts	20%	LDL cholesterol and small dense LDL particles	

Hypercholesterolemia	Very high CVD risks	20%	LDL cholesterol and small dense LDL particles	

Atherosclerosis	Arterial obstruction, chest pain	20–25% ABCA1 efflux	Polyunsaturated fat (PUFA) and carbohydrates, serum *γ*-glutamyl transferase activity, blood genomic profiling, and *α*4*β*7 integrin (LPAM-1)	[6, 7, 45–48]

Coronary heart disease	Monocytosis, high diabetics, hypertension, and chronic kidney diseases	20%,	Impaired sterol efflux, efflux capacity of high-density lipoprotein, HDL), myeloperoxidase increasing circulating HDL	[7]

Hyperglycaemia or type 1 diabetes	CVD and mortality	25%,	(TC), (TG), HDL, LDL, and anthropometric and biochemical parameters	[8]

Dyslipidemia	Hypoperfusion, high inflammation, and low BP	10%	TC, (TG), HDL, LDL, and anthropometric and biochemical parameters	[8, 42]

Atherosclerotic peripheral arterial disease	Prevalent, morbid, and mortal diseases	20% Shortening of lumen	LDL cholesterol	[9]

Ischemic heart disease (IHD)	Endothelial dysfunction, vascular inflammation	10–20%	Lipids, cholesterol, calcium, and cellular debris	[11]

Diastolic dysfunction and diastolic heart failure	Asymptomatic hypertension	20%	Myocardial remodeling	[12]

Chronic heart failures	ADP-induced platelet aggregation, triglycerides, end-diastolic volume, end-diastolic dimension, and ventricular septal thickness death	15–20%	Lipidemic, hemostasiological, and hemodynamic indicators, Willebrand factor, and D-dimer,	[13]

Myocardial infarction	Very high morbidity, severe pain	20–25%	Circulating microRNAs level in patients	[17]

Lipid stress and storage	Influence cholesterol availability in lipid rafts in immune cells	High LDL/HDL cholesterol levels	Omega-3 Index	[15, 16, 18]

Neuronal dysfunction	Neuronal cell death and neuroinflammatory	10–15%	27-hydroxycholesterol, plasma HDL, N-acylethanolamines (NAEs)	[21]

Transient global cerebral ischemia	Cardiac arrest and cardiovascular problems	5–10%	*ω*-3 PUFAs	[22]

Hypoglycaemia	Cardiac implications	5–10%	Elevated levels of Lp(a) and low HDL cholesterol	[23]

Hypertriglyceridemia/coronary artery disease (CAD)/acute coronary syndrome	Severe effect on BMR and peripheral and cardiac circulation	5–10%	Altered serum lipid	[1, 33]

HDL metabolism disorders	Severe inflammation and pain	5–10%	Lipid droplets (LDs)	[24]

Nephrotic syndrome	Renal filtration chocked	5–10%	LDL cholesterol, triglycerides, and Lp(a)	

Fatal myocardial infarction and brain stroke	Cardiovascular risks, morbidity, and mortality in elderly men	20–25%	Fat-specific protein Fsp27, fat storage-inducing transmembrane (FIT) proteins, seipin,and ADP-ribosylation factor 1-coat protein complex I	[24]

Systemic lupus erythematosus	Problem of PCV and hemoglobin	5%	Factors, proteins, ions, and stimulators of heart muscles	[34]

Acute myocardial infarction	Death of part of myocardial muscles, central chest pain, and severe crushing	20–25%	Serum soluble ST2 and interleukin-33	[36, 37]

Hypertension and dyslipidemia, hypercholesterolemia	Cardiovascular risk factors	15–20%	Total cholesterol and low-density lipoproteins	[41]

SCVRs	Tachyarrhythmias, bradyarrhythmias	5–10%	BP and LDL-C, high BMI	[43]

Hyperhomocysteinemia	High TC and pathogenesis	5%	LDL-C, HDL-C, TG, ApoAI, and ApoB Lp(a)	[44]

AVDs, type 2 diabetes, or metabolic syndrome	Increased levels of triglycerides, low levels of high density lipoprotein cholesterol, and postprandial lipemia	20–25%	MetS	[49, 50]

Procardiovascular risks, cardiovascular risks	Inflammation, obesity, and thrombosis	5–10%	Sedentary behavior, *β*-trace protein from GFR marker	[38, 40, 51]

Metabolic lipid disorders	Circulatory dysfunctions, high BP, peripheral pain, and high or low BMR	5–10%	MALDI-MS, imaging and lipidomics for clinical diagnosis, and proteome analysis	[52, 53]

Ischemic heart disease	Circulatory dysfunctions	Smoking, hypertension, age, family history	Endothelial dysfunction, monocyte accumulation, endothelial apoptosis, and thrombus formation	[54]

Low HDL-C syndromes	Increased risk of CAD	5%	Sphingomyelin phosphodiesterase 1 and glucocerebrosidase	[55]

Hypothyroidism and gall stone	Severe pain, inflammation	5%	TSH level and sodium and potassium salts	[56]

Multiple CVDs, diabetes, stroke, and recurrent ischaemia syndrome	Hepatic inflammation due to common carotid intima-media thickness	10–20%	Multiple biomarkers, vascular imaging	[57, 58]

Angina pectoris	Obesity, arterial thickness, BMI and respiration rate, and severe chest pain	10–20%	Coronary angiography	[24]

Antiphospholipid syndrome	Venous thrombosis	5%	microRNAs	[59, 60]

Myocardial infarction	Pregnancy associated plasma protein-A (PAPP-A in serum)	15%	Severe blood pressure changes, central chest pain, and silent or knocking angina	[61]

**Table 2:** Different risk indicating methods and biomarkers used for clinical diagnosis of cardiovascular disease and disorders.

Method	Sensitivity/biomarker efficiency	Confirmatory diagnosis	References
Coronary CT angiography	Highly sensitive	Cardiac characterization with contrast studies, pressure measurement, and O_2_ saturation. Detect valvular heart disease and intracardiac problems, biomarkers in acute coronary syndrome	[62, 63]

Spectral analysis of electrocardiography	Highly sensitive	Flow of blood across valves or septal defects are determined	[63]

Aortic wave reflection and pulse pressure amplification	Moderately	Heart rate, rhythm, volume, and accessing synchronicity of heart beats,	[64]

ECG	Highly sensitive	Cardiac arrhythmias and conduction abnormalities, LVH defects, mitral stenosis, pulmonary embolism, confirming ischaemic heart disease, and myocardial infarction	

CXR	Moderately sensitive	Identify cardiac enlargement and cardiac chamber defects	

**Imaging biomarkers**		Hemorrhagic complications, acute stroke	[65]

Palpography and elastography/thermography	Moderately sensitive	Demonstrate increased temperature at the site of inflamed, vulnerable plaques, and strain of fibrous caps	

Perfusion imaging	Moderately sensitive		

^31^P magnetic resonance spectroscopy	Highly sensitive	Intramyocellular lipid content, stroke, and characterization of adipose tissue	

Magnetic resonance imaging (MRI)	Highly sensitive	Coronary artery disease hypertrophic cardiomyopathy and heart wall motion abnormalities	

Framingham risk score	Highly sensitive		[66]

Intraplaque neovascularization	Moderately sensitive	Carotid atherosclerosis	[67]

Ultrasound (US) and computed tomography (CT)	Moderately sensitive	Stenoses, coronary calcification	

Bioluminescence, single-photon emission computed tomography (SPECT)	Highly sensitive	To estimate the 10-year risk of developing coronary heart disease	

Positron emission tomography (PET)	Highly sensitive	Oedema, inflammation, fibrosis, and IIM	[68, 69]

Cardiac magnetic resonance	Highly sensitive	Left atrial volume and function Feature-tracking cardiac magnetic resonance (CMR), maximal and minimal LA volume indexes, development of heart failure (HF) in asymptomatic individuals.	[70]

Unstable carotid plaque		High-risk for stroke patients	[71]

Pulse wave velocity	Aortic wall material/stiffness	Various cardiovascular pathologies	[72]

Transthoracic echocardiography/MRI	Highly sensitive	Atherosclerosis in the aorta and carotid arteries	

Coronary angiography	Moderately sensitive	Obstructive coronary artery disease	

Intravascular ultrasound (IVUS)	Highly sensitive		

Optical coherence tomography	Highly sensitive	Degree of inflammatory activity within plaque, measures the thickness of the fibrous cap	

MRI-visible perivascular spaces	Highly sensitive	Cerebral small vessel disease	[73]

Electroanatomic, fluoroscopy	Highly sensitive	Aging and cerebral small vessel disease	[73]

Electroanatomic mapping	Highly sensitive	Display catheter positions and stored electrograms jointly with anatomic information of the target heart chamber generated through other imaging modalities	[74]

Static FLAIR anatomical images	Highly sensitive	WM integrity	[75]

Visible retinal lesions	Highly sensitive	Dot-blot hemorrhages, retinal neovascularization	[76]

DM-AA score	Moderately sensitive	Development of CVD	[77]

Epicardial adipose/epicardial fat determination	Moderately sensitive	Coronary artery disease, atherogenic and inflammatory cytokines	[78]

Infantile hemangiography	Moderately sensitive	Capillaries blockage	[79]

Cerebral amyloid angiopathy	Moderately sensitive	Spontaneous intracerebral haemorrhage (ICH)	[27]

**Serum biomarkers**		*α*4*β*7 integrin (LPAM-1) and its ligand, mucosal addressin cell adhesion molecule (MAdCAM-1), focal adhesion kinase (FAK) and MAPK/ERK1/2 in macrophage	

	Triglyceride to HDL cholesterol ration	Lipid abnormalities	

	Total cholesterol (TC) to HDL-C ratio	Low to high cholesterol levels provide appropriate information about risks of cardiovascular diseases	[80]

	Fasting and nonfasting triglyceride levels	Mixed dyslipidemic patterns	

	PON1 activity/HDL-C ratio	Endothelial dysfunctions	[81]

	HDL, LDL, triglycerides and total cholesterol levels	Type 2 diabetes mellitus (T2DM)	

	Cholesterol, LDL-C, ApoB, Lp(a), and ApoA1/ApoB ratio	Endothelial dysfunctions, atherosclerosis	[80]

**Lipid-lipoprotein ratio**	Oxidized forms of LDL	Severe cardiovascular risks, atherogenic lipid particles	

	Levels of ApoB and ApoAI or ratio of ApoB : ApoAI	Myocardial infarction	

	Statin	Slowing progression of coronary atherosclerosis	[82]

	Lp-PLA2	Lp-PLA_2_ is platelet-activating factor (PAF) acetylhydrolase	

	ACAT acetyl cholesterol transferase	Fatty acid metabolism, synthesis and degradation of ketone bodies	[83]

	HDL protein	HDL particles remove fats and cholesterol from cells, including within artery wall atheroma, and transport it back to the liver for excretion or reutilization	[84]

	M-FABPs	Atherosclerosis	[85]

	Sterol regulatory DNA elements and binding with proteins	AT, CVD, and lipid homeostasis	[86]

	Specific mRNA level	Rapid utilization of fatty acids	[87]

	Enzyme acyl CoA: transferase	Increase and decrease in cholesterol level	

**HDLs and apolipoproteins level**			

	Non-high-density lipoprotein and low-density lipoprotein cholesterol	Cardiovascular diseases	[88, 89]

	Postprandial lipid profiles in combination with ApoB	Heterozygous familial hypercholesterolemia, familial combined hyperlipidemia, and familial hypertriglyceridemia	[90]

Sphingolipids	Sphingomyelin(SM) and sphingosine 1-phosphate (Sph-1-P)	Atherosclerotic disorders	[91]

**Lipid transfer particles as biomarkers **	** Different types of LTPs**	Transcytosis, transport, and signaling for lipid metabolism	[92]

Blood pressure (BP), height, weight, lipid profiles, blood glucose (BG), body mass index (BMI), fasting insulin (FINS), serum uric acid (SUA), and the urinary albumin/creatinine ratio (UACR)	Moderately sensitive	Metabolic disorders in nondiabetic elderly patients	[93]

	Plasma levels of high-density lipoprotein cholesterol	Coronary artery	[66]

	Framingham risk score (FRS)	Coronary artery	[66]

			
**Immunohistochemical markers**			

	MDM2 overexpression; MFAP4 in serum	Cardiovascular conditions	[94, 95]

	N-cadherin and connexin-43	Arrhythmogenic right ventricular cardiomyopathy (ARVC)	[96]

	ARVC plakoglobin		

	Intravascular papillary endothelial hyperplasia	Hyperplastic endothelial cells	[97]

	Localization of lipid binding proteins	Become good markers for CVDs	[98]

	StarD5 cholesterol binding proteins	lipid transport in health and disease, steroidogenic acute regulatory protein- (StAR-) related lipid-3 transfer (START) domain family	[99]

	Rho, Rho-kinase 1, and ROCK2	Regulating the shape and movement of cells by acting on the cytoskeleton	[100]

	Sterol regulatory element binding protein-2	Activated SREBPs t bind to specific sterol regulatory element DNA sequences. Indicating cellular cholesterol levels	[101]

	Endothelial markers CD31	Myocardial ischemia	[100]

**Oxidative stress-related biomarkers**			[102]

	Advanced oxidation protein products (AOPPs, circulating OGN and NGAL/MMP9 complex)	Increased OS	[103]

	TBARS, MetS	Oxidative stress, oxidative PTM	[104, 105]

	Reactive oxygen/nitrogen species/superoxide production	Neurodegeneration	[104]

	F2-IsoPs and F4-NPs	Neurodegenerative diseases	[106]

**Inflammatory biomarkers**			

	Level of chemokines and number of its receptors, and inflammatory lipids	CAD, atherosclerotic vascular disease, mainly chronic inflammation in the case of atherosclerosis	[107, 108]

	*γ*′ Fibrinogen acts	Thrombotic disease	[109]

	Interleukin-1 receptor-like 1 (ST2) and interleukin-33, and interleukin-1 receptor family	Cardiovascular risk, cardiovascular diseases ST2 is considered to play a causal role in chronic cardiovascular diseases such as atherosclerosis and heart failure. sST2 as a biomarker for adverse cardiovascular events,potential mechanistic role of the IL-33/ST2 pathway in chronic inflammatory cardiovascular disease Brachial artery flow-mediated dilation (FMD) is a reliable, non-invasive method of assessing endothelial function increased levels of the pro-inflammatory cytokine IL-6 display impaired endothelial function	[28, 110]

	IL1R1 and its ligand, IL1*β*, and elevated IL-6 levels	Cardiovascular disease, obesity, and infection, activating platelets and megakaryocytes to promote atherothrombosis. Cardiovascular risk factors	[111, 112]

	Complement C3	Cardiometabolic risk in psoriasis	[113]

	CCR5, chemokine expression of CCL2, CCL3, CCL5 and CXCL10, and RAGE and FABP4	Prognostic biomarker for plaque stability	[114–116]

	*β*2 integrins (CD11/CD18)	Acute inflammation	

	VCAM-1, ICAM-1 MCP-1, VCAM-1, ICAM-1, IL-1, and TNF-*α*	Inflammation, arteriosclerosis, and lipid peroxidation problems	[117]

	C-reactive protein	Inflammation	[118]

	Myeloperoxidase MPO	Leukocyte-derived prooxidant enzyme	[119]

	PAF-AH	Inflammatory markers, oxidation of phospholipids	[119]

	Accumulation of free radicals	Posttraumatic stress disorder (PTSD)	

	Interleukin-33, copeptin	Heart failure	[120, 121]

**ST2 as CVD markers**			

	Transmembrane receptor (ST2L)	Endothelial intima thickness	[122]

	ST2 and galectin-3 (Gal-3)	Heart failure	[123]

	ST2, GDF-15, and hsTnI	Atrial fibrillation	[124, 125]

	Copeptin, MR-proADM, abd MR- proANP	CV death and HF	[126]

**Physiological markers**			

	Enzyme indoleamine 2,3-dioxygenase (IDO)	Cellular immune response, ROS production by activated immune effector cells like macrophages	[127]

	RAAS	Development and progression of CVD, cardiovascular and renal diseases, and inflammation	[128]

	Highly toxic peroxynitrite (ONOO(−), superoxide anion (O_2_(−))	Vascular inflammation	[128]

	Neopterin and kynurenine to tryptophan ratio	Robust markers of immune activation *in vitro*, IDO enzyme activity	[127]

	(ACE)/angiotensin II (Ang II)/AT1	Vasoconstriction, cell proliferation, and fibrosis	[129]

	Erbin	Inhibitor of pathological cardiac hypertrophy	[130]

	Endothelial MPs, E-selectin; CD51; CD105	Potential biomarkers for COPD	[131]

	Serum concentrations of factor I–XII, platelet count	Evaluate hemostasis in pregnancy	[132]

	STIM1	Normal cardiac function, regulation of ER, and mitochondrial function	[133]

	Blood pressure, fasting glucose, HDL, LDL, or C-reactive protein levels, insulin, triglyceride, and cholesterol	Higher risk of CVD	

	Vitamin D status	Cardiometabolic problems	[62]

**Anatomical markers**			

	**Left ventricular hypertrophy**	High risk of CVD, left ventricular systolic dysfunction	[134]

	Galectin-3/plasma galectin-3 levels	Outflow tract tachycardias, hypertrophied hearts	[135, 136]

**Omega-3 Index as biomarker**	(eicosapentaenoic acid + docosahexaenoic acid) content in red blood cell membranes	Acute coronary	[137]

n-3 PUFA	Plasma phospholipid and dietary alpha linolenic acid, DHA	Increasing n-3 LC-PUFA in RBC and plasma lipids, improving cardiovascular health, and significantly decreasing plasma triglyceride levels	[138–140]

	n-3 fatty acid *α*-linolenic acid	Cardiometabolic risk factors, congestive heart failure	[141]

	Omega-3 fatty acids	Positive effects on weight, systolic blood pressure, lipid profile, and markers of inflammation and autoimmunity	[142, 143]

**Genetic markers**	Rare genetic variants	Lipid related morbidities, genes	[144]

	Mutations causing low LDL-cholesterol and ApoB	CVD progression, lipid metabolism	[145]

	Mutations in ApoB, proprotein convertase subtilisin/kexin type 9 MTP genes, and ANGPTL3 gene	Familial combined hypolipidemia	[145]

	Sterol response element-binding protein	LDL-cholesterol (LDL-C) uptake	[146]

	Analysis of polymorphism of genes	Coronary heart disease, ApoB; ApoC111; ApoE; X2 of ApoB; and S2 of ApoCIII	[147]

	Human ApoE gene e2, e3, and e4	Impaired lipid metabolism	[148]

	MicroRNAs	Diagnostic and prognostic biomarkers in atherosclerosis	[149]

	Missense mutation in MED30 gene; MED1, MED13, MED14, MED15, MED23, MED25, and CDK8	CVD-related metabolic disorders, progressive cardiomyopathy	[150]

DNA based diagnostics	Genetic variants in known lipid genes	Severe dyslipidemias	[151]

**Lipid metabolomics**	Metabolomics	Study design and data analysis in diabetes, CVDs, HF, and obesity research	

LC-MS/MS-based metabololipidomics	Resolvin (Rv) D1, RvD2, protectin (PD) 1, and lipoxin (LX) A4	Lipid disorders, genetic variance and intermediate metabolic phenotypes, peripheral vascular disease, PD1, and 17-HDHA levels	[152]

	by LDLRAP1, ApoA5, ANGPLT3/4, and PCSK9	Modulators or adaptor proteins	[153]

	Functional mutations in ApoB	Long elevated LDL levels, risk of heart disease	[154]

	MicroRNAs	HFpEF versus HFrEF markers	[155]

**Therapeutic markers**			

	Hs-cTnI	Multiple analysis, troponin I	[70]

	NT-proBNP and sST2	Cardiac death (SCD) risk, ICD therapy implantable cardioverter defibrillator	[156]

	Standard lipid-lowering therapies	Age-related vascular disorders	[157]

	Adiponectin, Local coronary circulation	Antiatherosclerotic properties	[158]

	Multiple high-resolution molecular imaging modalities	Abdominal aortic aneurysm disease Multiple high-resolution molecular imaging modalities capable of tracking disease progression, quantifying the role of inflammation, and evaluating the effects of potential therapeutics anatomical imaging, which include ultrasound (US) and computed tomography (CT), previous molecular imaging efforts have used magnetic resonance (MR), near-infrared fluorescence (NIRF), bioluminescence, single-photon emission computed tomography (SPECT), and positron emission tomography (PET)	[68]

	Hormone therapy	Biochemical and ultrasound markers of endothelial function, improving DSD patients	[159]

	VE-cadherin, E-selectin, thrombomodulin, and vWf	Improve endothelial functions	[159]

	PLA2G2E	Act as metabolic coordinators	[160]

	Flippase, ATP13A2	Local lipid dynamics during vesicle formation and membrane fusion events	[161]

	Consumption index of various fatty acids	Coronary heart diseases	[162]

	Docosahexaenoic acid (DHA)	Therapeutic marker, reduce the risk of cardiovascular disease	[163]

	Omega-3 Index	Emerging as a risk factor for fatal and nonfatal cardiovascular events, therapeutic markers	[164]

## Abbreviations

HoFH: Hypercholesterolemia
LDL: Low-density lipoprotein
HDL: High-density lipoproteins
TG: Triglycerides
IHD: Ischemic heart disease
PUFAs: Polyunsaturated fatty acids
LDs: Lipid droplets
Fsp27: Fat-specific protein
FIT: Fat storage-inducing transmembrane
apo: Apolipoprotein
LDLc: Low-density lipoprotein cholesterol
Lp(a): Lipoprotein (a)
BMI: Body mass index
GGT: Glutamyl transferase activity
LPL: Lipoprotein lipase
ANGPTL3: Angiopoietin-like 3 proteins
HL: Hepatic lipase
EAT: Epicardial adipose tissue
PAD: Peripheral artery disease
PAPP-A: Pregnancy associated plasma protein-A
Lp-PLA2: Lipoprotein-associated phospholipase A2
M-FABPs: Mammalian fatty acid binding protein
ASCVD: Atherosclerotic cardiovascular disease
SCP2: Sterol carrier protein
PON: Paraoxonase
NAFLD: Nonalcoholic fatty liver disease
SF: Serum ferritin
FINS: Fasting insulin
SUA: Serum uric acid
PP: Postprandial
FRS: Framingham risk score
ARVC: Arrhythmogenic right ventricular cardiomyopathy
CBPs: Cholesterol binding proteins
AOPPs: Advanced oxidation protein products
DCM: Diabetic cardiomyopathy
CCR5: Chemokine receptor 5
CRP: C-reactive protein
PTSD: Posttraumatic stress disorder
IL: Interleukin
GDF: Growth differentiation factor
AF: Atrial filtration
LVH: Left ventricular hypertrophy
LVSD: Left ventricular systolic dysfunction
LDLR: Low-density lipoprotein receptor
LA: Left atrial
IMT: Intimal-medial thickness
OCT: Optical coherence tomography
NO: Nitric oxide
PVS: Perivascular spaces
TIA: Transient ischaemic attack
EAMS: Electroanatomic mapping systems
ECS: Endocannabinoid system
HT: Hormone therapy
DHA: Docosahexaenoic acid
AMI: Acute myocardial infarction
MetS: Metabolic syndrome
CMBs: Cerebral microbleeds
NAEs: N-Acylethanolamines
MMP: Metalloproteinases
KD: Ketogenic diet
saRHI: Small artery reactive hyperemia index
TLR: Toll-like receptor
SREBP-1: Sterol regulatory element binding protein-1
FAS: Fatty acid synthetase
UCP-2: Uncoupling protein-2
TGN: Trans-Golgi network
ERC: Endocytic recycling compartment.

## References

[1] Bamba V. (2014). Update on screening, etiology, and treatment of dyslipidemia in children. *The Journal of Clinical Endocrinology & Metabolism*.

[2] Brucker N., Charão M. F., Moro A. M. (2014). Atherosclerotic process in taxi drivers occupationally exposed to air pollution and co-morbidities. *Environmental Research*.

[3] Brown T. M., Bittner V. (2008). Biomarkers of atherosclerosis: clinical applications. *Current Cardiology Reports*.

[4] Cooper J. A., Miller G. J., Humphries S. E. (2005). A comparison of the PROCAM and Framingham point-scoring systems for estimation of individual risk of coronary heart disease in the Second Northwick Park Heart Study. *Atherosclerosis*.

[5] Grundy S. M., D'Agostino R. B., Mosca L. (2001). Cardiovascular risk assessment based on US cohort studies: findings from a National Heart, Lung, and Blood Institute Workshop. *Circulation*.

[6] Kalantarian S., Rimm E. B., Herrington D. M., Mozaffarian D. (2014). Dietary macronutrients, genetic variation, and progression of coronary atherosclerosis among women. *American Heart Journal*.

[7] Shao B., Tang C., Sinha A. (2014). Humans with atherosclerosis have impaired ABCA1 cholesterol efflux and enhanced high-density lipoprotein oxidation by myeloperoxidase. *Circulation Research*.

[8] Distiller L. A. (2014). Why do some patients with type 1 diabetes live so long?. *World Journal of Diabetes*.

[9] Mueller T., Hinterreiter F., Luft C., Poelz W., Haltmayer M., Dieplinger B. (2014). Mortality rates and mortality predictors in patients with symptomatic peripheral artery disease stratified according to age and diabetes. *Journal of Vascular Surgery*.

[10] Unis A., Abdelbary A., Hamza M. (2014). Comparison of the effects of escitalopram and atorvastatin on diet-induced atherosclerosis in rats. *Canadian Journal of Physiology and Pharmacology*.

[11] Hadi N. R., Mohammad B. I., Ajeena I. M., Sahib H. H. (2013). Antiatherosclerotic potential of clopidogrel: antioxidant and anti-inflammatory approaches. *BioMed Research International*.

[12] Collier P., Watson C. J., Voon V. (2011). Can emerging biomarkers of myocardial remodelling identify asymptomatic hypertensive patients at risk for diastolic dysfunction and diastolic heart failure?. *European Journal of Heart Failure*.

[13] Kachkovskiǐ M. A., Simerzin V. V., Rybanenko O. A., Kirichenko N. A. (2014). Hemostasiological, lipidemic, and hemodynamic indicators associated with the risk of cardiovascular death in high- and very high-risk patients according to the SCORE scale. *Terapevticheskiǐ arkhiv*.

[14] Whelton S. P., Narla V., Blaha M. J. (2014). Association between resting heart rate and inflammatory biomarkers (high-sensitivity c-reactive protein, interleukin-6, and fibrinogen) (from the multi-ethnic study of atherosclerosis). *The American Journal of Cardiology*.

[15] Rubenfire M., Brook R. D. (2013). HDL cholesterol and cardiovascular outcomes: what is the evidence?. *Current Cardiology Reports*.

[16] Catapano A. L., Pirillo A., Bonacina F., Norata G. D. (2014). HDL in innate and adaptive immunity. *Cardiovascular Research*.

[17] Zampetaki A., Willeit P., Tilling L. (2012). Prospective study on circulating microRNAs and risk of myocardial infarction. *Journal of the American College of Cardiology*.

[18] von Schacky C. (2014). Omega-3 index and cardiovascular health. *Nutrients*.

[19] Heringlake M., Charitos E. I., Gatz N. (2013). Growth differentiation factor 15: a novel risk marker adjunct to the EuroSCORE for risk stratification in cardiac surgery patients. *Journal of the American College of Cardiology*.

[20] Grundy S. M. (2012). Use of emerging lipoprotein risk factors in assessment of cardiovascular risk. *The Journal of the American Medical Association*.

[21] Luo C., Ren H., Wan J.-B. (2014). Enriched endogenous omega-3 fatty acids in mice protect against global ischemia injury. *The Journal of Lipid Research*.

[22] Esposito E., Cordaro M., Cuzzocrea S. (2014). Roles of fatty acid ethanolamides (FAE) in traumatic and ischemic brain injury. *Pharmacological Research*.

[23] Hanefeld M., Duetting E., Bramlage P. (2013). Cardiac implications of hypoglycaemia in patients with diabetes—a systematic review. *Cardiovascular Diabetology*.

[24] Tan J. S. Y., Seow C. J. P., Goh V. J., Silver D. L. (2014). Recent advances in understanding proteins involved in lipid droplet formation, growth and fusion. *Journal of Genetics and Genomics*.

[25] Gupta S., Gudapati R., Gaurav K., Bhise M. (2013). Emerging risk factors for cardiovascular diseases: Indian context. *Indian Journal of Endocrinology and Metabolism*.

[26] Rich-Edwards J. W., Fraser A., Lawlor D. A., Catov J. M. (2014). Pregnancy characteristics and women's future cardiovascular health: an underused opportunity to improve women's health?. *Epidemiologic Reviews*.

[27] Hodcroft C. J., Rossiter M. C., Buch A. N. (2014). Cannabis-associated myocardial infarction in a young man with normal coronary arteries. *The Journal of Emergency Medicine*.

[28] Weiner S. D., Ahmed H. N., Jin Z. (2014). Systemic inflammation and brachial artery endothelial function in the Multi-Ethnic Study of Atherosclerosis (MESA). *Heart*.

[29] Hurks R., Vink A., Hoefer I. E. (2014). Atherosclerotic risk factors and atherosclerotic postoperative events are associated with low inflammation in abdominal aortic aneurysms. *Atherosclerosis*.

[30] Oni E. T., Agatston A. S., Blaha M. J. (2013). A systematic review: burden and severity of subclinical cardiovascular disease among those with nonalcoholic fatty liver; should we care?. *Atherosclerosis*.

[31] Nursalim A., Suryaatmadja M., Panggabean M. (2013). Potential clinical application of novel cardiac biomarkers for acute myocardial infarction. *Acta Medica Indonesiana*.

[32] Eggers K. M., Al-Shakarchi J., Berglund L. (2013). High-sensitive cardiac troponin T and its relations to cardiovascular risk factors, morbidity, and mortality in elderly men. *American Heart Journal*.

[33] O'Malley R. G., Bonaca M. P., Scirica B. M. (2014). Prognostic performance of multiple biomarkers in patients with non-ST-segment elevation acute coronary syndrome: analysis from the MERLIN-TIMI 36 trial (Metabolic Efficiency with Ranolazine for Less Ischemia in Non-ST-Elevation Acute Coronary Syndromes-Thrombolysis in Myocardial Infarction 36). *Journal of the American College of Cardiology*.

[34] Lippi G., Cervellin G. (2014). Risk assessment of post-infarction heart failure. Systematic review on the role of emerging biomarkers. *Critical Reviews in Clinical Laboratory Sciences*.

[35] Miner M., Nehra A., Jackson G. (2014). All men with vasculogenic erectile dysfunction require a cardiovascular workup. *The American Journal of Medicine*.

[36] Zhang Q., Xiao X., Li M. (2014). Berberine moderates glucose metabolism through the GnRH-GLP-1 and MAPK pathways in the intestine. *BMC Complementary and Alternative Medicine*.

[37] O'Meara E., de Denus S., Rouleau J.-L., Desai A. (2013). Circulating biomarkers in patients with heart failure and preserved ejection fraction. *Current Heart Failure Reports*.

[38] Samad F., Ruf W. (2013). Inflammation, obesity, and thrombosis. *Blood*.

[39] López-Mejías R., Genre F., González-Juanatey C., González-Gay M. A. (2014). Autoantibodies and biomarkers of endothelial cell activation in atherosclerosis. *Vasa*.

[40] Saunders T. J., Chaput J.-P., Tremblay M. S. (2014). Sedentary behaviour as an emerging risk factor for cardiometabolic diseases in children and youth. *Canadian Journal of Diabetes*.

[41] Sakurai H., Hanyu H. (2014). Lipid abnormality. *Nihon Rinsho*.

[42] Paltsev A. I., Myakotnykh V. S., Torgashov M. N. (2014). Blood lipid parameters in combat veterans with posttraumatic stress disorders. *Terapevticheskiǐ arkhiv*.

[43] Chopra I., Kamal K. M. (2014). Factors associated with therapeutic goal attainment in patients with concomitant hypertension and dyslipidemia. *Hospital Practice*.

[44] Baszczuk A., Musialik K., Kopczyński J. (2014). Hyperhomocysteinemia, lipid and lipoprotein disturbances in patients with primary hypertension. *Advances in Medical Sciences*.

[45] Bradley R., Fitzpatrick A. L., Jenny N. S., Lee D.-H., Jacobs D. R. (2013). Associations between total serum GGT activity and metabolic risk: MESA. *Biomarkers in Medicine*.

[46] Lee I.-T., Wang C.-Y., Huang C.-N., Fu C.-C., Sheu W. H.-H. (2013). High triglyceride-to-HDL cholesterol ratio associated with albuminuria in type 2 diabetic subjects. *Journal of Diabetes and Its Complications*.

[47] Daubail B., Durier J., Jacquin A. (2014). Factors associated with early recurrence at the first evaluation of patients with transient ischemic attack. *Journal of Clinical Neuroscience*.

[48] Zhi K., Li M., Zhang X. (2014). *α*4*β*7 integrin (LPAM-1) is upregulated at atherosclerotic lesions and is involved in
atherosclerosis progression. *Cellular Physiology and Biochemistry*.

[49] Jaiswal M., Schinske A., Pop-Busui R. (2014). Lipids and lipid management in diabetes. *Best Practice and Research: Clinical Endocrinology and Metabolism*.

[50] Mitu O., Roca M., Leon M. M., Mitu F. (2014). Predictive value of a positive exercise stress testing and correlations withcardiovascular risk factors. *Revista Medico-Chirurgicala a Societatii de Medici si Naturalisti din Iasi*.

[51] Orenes-Piñero E., Manzano-Fernández S., López-Cuenca Á., Marín F., Valdés M., Januzzi J. L. (2013). *β* -trace protein: from GFR marker to cardiovascular risk predictor. *Clinical Journal of the American Society of Nephrology*.

[52] Arafah K., Longuespée R., Desmons A., Kerdraon O., Fournier I., Salzet M. (2014). Lipidomics for clinical diagnosis: dye-assisted laser desorption/ionization (DALDI) method for lipids detection in MALDI mass spectrometry imaging. *OMICS*.

[53] di Girolamo F., del Chierico F., Caenaro G., Lante I., Muraca M., Putignani L. (2012). Human serum proteome analysis: new source of markers in metabolic disorders. *Biomarkers in Medicine*.

[54] Rye K.-A. (2014). Biomarkers associated with high-density lipoproteins in atherosclerotic kidney disease. *Clinical and Experimental Nephrology*.

[55] Ahmadzadeh A., Azizi F. (2014). Genes associated with low serum high-density lipoprotein cholesterol. *Archives of Iranian Medicine*.

[56] Ajdarkosh H., Khansari M. R., Sohrabi M. R. (2013). Thyroid dysfunction and choleduocholithiasis. *Middle East Journal of Digestive Diseases*.

[57] Wu J., Zhang H., Zheng H., Jiang Y. (2014). Hepatic inflammation scores correlate with common carotid intima-media thickness in rats with NAFLD induced by a high-fat diet. *BMC Veterinary Research*.

[58] Fonville S., Zandbergen A. A., Koudstaal P. J., den Hertog H. M. (2014). Prediabetes in patients with stroke or transient ischemic attack: prevalence, risk and clinical management. *Cerebrovascular Diseases*.

[59] Virupannavar S., Brandau A., Guggenheim C., Laird-Fick H. (2014). Possible association of etanercept, venous thrombosis, and induction of antiphospholipid syndrome. *Case Reports in Rheumatology*.

[60] Peng Y. (2014). Expert consensus on blood pressure management of diabetic patients in China. *Journal of Diabetes*.

[61] Bowring J., Mahto M., Mandal D., Baker P. N. (2008). Stroke in pregnancy associated with syphilis. *Journal of Obstetrics and Gynaecology Research*.

[62] Mihl C., Loeffen D., Versteylen M. O. (2014). Automated quantification of epicardial adipose tissue (EAT) in coronary CT angiography; comparison with manual assessment and correlation with coronary artery disease. *Journal of Cardiovascular Computed Tomography*.

[63] Chan C. P. Y., Rainer T. H. (2013). Pathophysiological roles and clinical importance of biomarkers in acute coronary syndrome. *Advances in Clinical Chemistry*.

[64] Kew S., Hamilton J. K., Ye C., Hanley A. J., Zinman B., Retnakaran R. (2013). Vitamin D status and cardiometabolic assessment in infancy. *Pediatric Research*.

[65] Sillanpää N. (2013). Role of perfusion imaging in acute stroke. *Panminerva Medica*.

[66] Feitosa M. F., Wojczynski M. K., Straka R. (2014). Genetic analysis of long-lived families reveals novel variants influencing high density-lipoprotein cholesterol. *Frontiers in Genetics*.

[67] van den Oord S. C., Akkus Z., Renaud G. (2014). Assessment of carotid atherosclerosis, intraplaque neovascularization, and plaque ulceration using quantitative contrast-enhanced ultrasound in asymptomatic patients with diabetes mellitus. *European Heart Journal—Cardiovascular Imaging*.

[68] Ramaswamy A. K., Hamilton M., Joshi R. V. (2013). Molecular imaging of experimental abdominal aortic aneurysms. *The Scientific World Journal*.

[69] Mavrogeni S., Sfikakis P. P., Dimitroulas T., Kolovou G., Kitas G. D. (2014). Cardiac and muscular involvement in idiopathic inflammatory myopathies: noninvasive diagnostic assessment and the role of cardiovascular and skeletal magnetic resonance imaging. *Inflammation & Allergy-Drug Targets*.

[70] Apple F. S., Steffen L. M., Pearce L. A., Murakami M. M., Luepker R. V. (2012). Increased cardiac troponin i as measured by a high-sensitivity assay is associated with high odds of cardiovascular death: the Minnesota Heart Survey. *Clinical Chemistry*.

[71] Naylor A. R. (2014). Identifying the high-risk carotid plaque. *Journal of Cardiovascular Surgery*.

[72] Shahmirzadi D., Konofagou E. E. (2014). Quantification of arterial wall inhomogeneity size, distribution, and modulus contrast using FSI numerical pulse wave propagation. *Artery Research*.

[73] Ali O. A., Chapman M., Nguyen T. H. (2014). Interactions between inflammatory activation and endothelial dysfunction selectively modulate valve disease progression in patients with bicuspid aortic valve. *Heart*.

[74] Bourier F., Fahrig R., Wang P. (2014). Accuracy assessment of catheter guidance technology in electrophysiology procedures: a comparison of a new 3D-based fluoroscopy navigation system to current electroanatomic mapping systems. *Journal of Cardiovascular Electrophysiology*.

[75] Makedonov I., Black S. E., MacIntosh B. J. (2013). BOLD fMRI in the white matter as a marker of aging and small vessel disease. *PLoS ONE*.

[76] Berkowitz B. A., Bissig D., Dutczak O., Corbett S., North R., Roberts R. (2013). MRI biomarkers for evaluation of treatment efficacy in preclinical diabetic retinopathy. *Expert Opinion on Medical Diagnostics*.

[77] Zheng Y., Wang L., Krupka T. M. (2013). The feasibility of using high frequency ultrasound to assess nerve ending neuropathy in patients with diabetic foot. *European Journal of Radiology*.

[78] de Graaf M. A., Broersen A., Kitslaar P. H. (2013). Automatic quantification and characterization of coronary atherosclerosis with computed tomography coronary angiography: cross-correlation with intravascular ultrasound virtual histology. *International Journal of Cardiovascular Imaging*.

[79] Osaki T. H., Jakobiec F. A., Mendoza P. R., Lee Y., Fay A. M. (2013). Immunohistochemical investigations of orbital infantile hemangiomas and adult encapsulated cavernous venous lesions (Malformation versus hemangioma). *Ophthalmic Plastic and Reconstructive Surgery*.

[80] Zhan Y., Yu J., Ding R., Sun Y., Hu D. (2014). Triglyceride to high density lipoprotein cholesterol ratio, total cholesterol to high density lipoprotein cholesterol ratio and low ankle brachial index in an elderly population. *Vasa*.

[81] Fekih O., Triki S., Hellara I. (2014). Can paraoxonase 1 polymorphisms (L55 M and Q192 R) protect children with type 1 diabetes against lipid abnormalities?. *Journal of Clinical Lipidology*.

[82] Lepedda A. J., Zinellu A., Nieddu G. (2014). Human serum albumin Cys34 oxidative modifications following infiltration in the carotid atherosclerotic plaque. *Oxidative Medicine and Cellular Longevity*.

[83] Chang C. C. Y., Huh H. Y., Cadigan K. M., Chang T. Y. (1993). Molecular cloning and functional expression of human acyl-coenzyme A:cholesterol acyltransferase cDNA in mutant Chinese hamster ovary cells. *The Journal of Biological Chemistry*.

[84] Armando J., Mendez Oram J. F., Bieman E. L. (1991). Protein kinase C as a mediator of high density lipoprotein receptor dependent efflux of intracellular cholesterol. *The Journal of Biological Chemistry*.

[85] Miller W. C., Hickson R. C., Bass N. M. (1988). Fatty acid binding proteins in the three types of rat skeletal muscle. *Proceedings of the Society for Experimental Biology and Medicine*.

[86] Sakai J., Rawson R. B. (2001). The sterol regulatory element-binding protein pathway: control of lipid homeostasis through regulated intracellular transport. *Current Opinion in Lipidology*.

[87] Haunerland N. H., Andolfatto P., Chisholm J. M., Wang Z., Chen X. (1992). Development changes in muscle FABP expression concentration and intracellular distribution in the desert locust, *Schistocerca gregaria*. *European Journal of Biochemistry*.

[88] Shokrani M. (2014). Emerging approaches to the examination of lipoproteins for cardio-metabolic risk stratification. *MLO: Medical Laboratory Observer*.

[89] Ram C. V. S. (2013). Fixed-dose triple-combination treatments in the management of hypertension. *Managed Care*.

[90] Stenovec M., Trkov S., Kreft M., Zorec R. (2014). Alterations of calcium homoeostasis in cultured rat astrocytes evoked by bioactive sphingolipids. *Acta Physiologica*.

[91] Ohkawa R., Kurano M., Nakamura K. (2013). Sphingolipids, possible biomarkers for atherosclerotic disorders. *Rinsho Byori*.

[92] Ehnholm C., Mahley R. W., Chappell D. A., Weisgraber K. H., Ludwig E., Witztum J. L. (1984). Role of apolipoprotein E in the lipolytic conversion of *β*-very low density lipoproteins to low density lipoproteins in type III hyperlipoproteinemia. *Proceedings of the National Academy of Sciences of the United States of America*.

[93] Li B., Lin W., Lin N., Dong X., Liu L. (2014). Study of the correlation between serum ferritin levels and the aggregation of metabolic disorders in non-diabetic elderly patients. *Experimental and Therapeutic Medicine*.

[94] Neuville A., Collin F., Bruneval P. (2014). Intimal sarcoma is the most frequent primary cardiac sarcoma: clinicopathologic and molecular retrospective analysis of 100 primary cardiac sarcomas. *The American Journal of Surgical Pathology*.

[95] Wulf-Johansson H., Johansson S. L., Schlosser A. (2013). Localization of microfibrillar-associated protein 4 (MFAP4) in human tissues: clinical evaluation of serum MFAP4 and its association with various cardiovascular conditions. *PLoS ONE*.

[96] Kwon Y.-S., Park T. I., Cho Y., Bae M. H., Kim S. (2013). Clinical usefulness of immunohistochemistry for plakoglobin, N-cadherin, and connexin-43 in the diagnosis of arrhythmogenic right ventricular cardiomyopathy. *International Journal of Clinical and Experimental Pathology*.

[97] Akdur N. C., Donmez M., Gozel S., Ustun H., Hucumenoglu S. (2013). Intravascular papillary endothelial hyperplasia: histomorphological and immunohistochemical features. *Diagnostic Pathology*.

[98] Upadhyay R. K., Agarwal H. C. (2007). Role of lipoproteins in cholesterol trafficking in insects: a review. *Journal of Applied Biosciences*.

[99] Pandak W. M., Rodriguez-Agudo D., Ren S. (2006). Localization of StarD5 cholesterol binding protein. *Journal of Lipid Research*.

[100] Guo X. R., Zheng S. C., Liu L., Feng Q. L. (2009). The sterol carrier protein 2/3-oxoacyl-CoA thiolase (SCPx) is involved in cholesterol uptake in the midgut of Spodoptera litura: Gene cloning, expression, localization and functional analyses. *BMC Molecular Biology*.

[101] Kim J.-H., Ong W.-Y. (2009). Localization of the transcription factor, sterol regulatory element binding protein-2 (SREBP-2) in the normal rat brain and changes after kainate-induced excitotoxic injury. *Journal of Chemical Neuroanatomy*.

[102] Piwowar A. (2014). Biochemical and clinical aspects of advanced oxidation protein products in kidney diseases and metabolic disturbances. *Postępy Higieny i Medycyny Doświadczalnej*.

[103] Cheng C.-Y., Su S.-Y., Tang N.-Y., Ho T.-Y., Chiang S.-Y., Hsieh C.-L. (2008). Ferulic acid provides neuroprotection against oxidative stress-related apoptosis after cerebral ischemia/reperfusion injury by inhibiting ICAM-1 mRNA expression in rats. *Brain Research*.

[104] Méndez L., Pazos M., Giralt M. (2014). Targets of protein carbonylation in spontaneously hypertensive obese Koletsky rats and healthy Wistar counterparts: a potential role on metabolic disorders. *Journal of Proteomics*.

[105] Dzięgielewska-Gęsiak S., Wysocka E., Michalak S., Nowakowska-Zajdel E., Kokot T., Muc-Wierzgoń M. (2014). Role of lipid peroxidation products, plasma total antioxidant status, and Cu-, Zn-superoxide dismutase activity as biomarkers of oxidative stress in elderly prediabetics. *Oxidative Medicine and Cellular Longevity*.

[106] Miller E., Morel A., Saso L., Saluk J. (2014). Isoprostanes and neuroprostanes as biomarkers of oxidative stress in neurodegenerative diseases. *Oxidative Medicine and Cellular Longevity*.

[107] Stoner L., Lucero A. A., Palmer B. R., Jones L. M., Young J. M., Faulkner J. (2013). Inflammatory biomarkers for predicting cardiovascular disease. *Clinical Biochemistry*.

[108] Rolin J., Maghazachi A. A. (2014). Implications of chemokines, chemokine receptors, and inflammatory lipids in atherosclerosis. *Journal of Leukocyte Biology*.

[109] Farrell D. H. (2012). *γ*′ Fibrinogen as a novel marker of thrombotic disease. *Clinical Chemistry and Laboratory Medicine*.

[110] Willems S., Hoefer I., Pasterkamp G. (2012). The role of the Interleukin 1 receptor-like 1 (ST2) and Interleukin-33 pathway in cardiovascular disease and cardiovascular risk assessment. *Minerva Medica*.

[111] Beaulieu L. M., Lin E., Mick E. (2014). Interleukin 1 receptor 1 and interleukin 1*β* regulate megakaryocyte maturation, platelet activation, and transcript profile during inflammation in mice and humans. *Arteriosclerosis, Thrombosis, and Vascular Biology*.

[112] Desai N. R., Kohli P., Giugliano R. P. (2013). AMG145, a monoclonal antibody against proprotein convertase subtilisin kexin type 9, significantly reduces lipoprotein(a) in hypercholesterolemic patients receiving statin therapy: an analysis from the LDL-C assessment with proprotein convertase subtilisin kexin type 9 monoclonal antibody inhibition combined with statin therapy (LAPLACE)-thrombolysis in myocardial infarction (TIMI) 57 Trial. *Circulation*.

[113] Torres T., Bettencourt N., Mendonça D., Vasconcelos C., Silva B. M., Selores M. (2014). Complement C3 as a marker of cardiometabolic risk in psoriasis. *Archives of Dermatological Research*.

[114] Luehmann H. P., Pressly E. D., Detering L. (2014). PET/CT imaging of chemokine receptor CCR5 in vascular injury model using targeted nanoparticle. *Journal of Nuclear Medicine*.

[115] Stålman A., Bring D., Ackermann P. W. (2014). Chemokine expression of CCL2, CCL3, CCL5 and CXCL10 during early inflammatory tendon healing precedes nerve regeneration: an immunohistochemical study in the rat. *Knee Surgery, Sports Traumatology, Arthroscopy*.

[116] Hardaway A. L., Podgorski I. (2013). IL-1*β*, RAGE and FABP4: targeting the dynamic trio in metabolic inflammation and related pathologies. *Future Medicinal Chemistry*.

[117] Nibbs R. J. B., Graham G. J. (2013). Immune regulation by atypical chemokine receptors. *Nature Reviews Immunology*.

[118] Lee B. S., Kim S. H., Oh J. (2014). C-reactive protein inhibits survivin expression via Akt/mTOR pathway downregulation by PTEN expression in cardiac myocytes. *PLoS ONE*.

[119] Marathe G. K., Pandit C., Lakshmikanth C. L., Chaithra V. H., Jacob S. P., D'Souza C. J. M. (2014). To hydrolyse or not to hydrolyse: the dilemma of platelet activating factor acetylhydrolase (PAF-AH). *The Journal of Lipid Research*.

[120] Dhillon O. S., Narayan H. K., Quinn P. A., Squire I. B., Davies J. E., Ng L. L. (2011). Interleukin 33 and ST2 in non-ST-elevation myocardial infarction: comparison with Global Registry of Acute Coronary Events Risk Scoring and NT-proBNP. *American Heart Journal*.

[121] Vasile V. C., Jaffe A. S. (2014). Emerging biomarkers for acute heart conditions. *Current Opinion in Cardiology*.

[122] Januzzi J. L. (2013). ST2 as a cardiovascular risk biomarker: from the bench to the bedside. *Journal of Cardiovascular Translational Research*.

[123] Dhillon O. S., Narayan H. K., Khan S. Q. (2013). Pre-discharge risk stratification in unselected STEMI: is there a role for ST2 or its natural ligand IL-33 when compared with contemporary risk markers?. *International Journal of Cardiology*.

[124] Rienstra M., Yin X., Larson M. G. (2014). Relation between soluble ST2, growth differentiation factor-15, and high-sensitivity troponin I and incident atrial fibrillation. *The American Heart Journal*.

[125] Ho J. E., Hwang S. J., Wollert K. C. (2013). Biomarkers of cardiovascular stress and incident chronic kidney disease. *Clinical Chemistry*.

[126] Young D., Camhi S., Wu T., Hagberg J., Stefanick M. (2013). Relationships among changes in C-reactive protein and cardiovascular disease risk factors with lifestyle interventions. *Nutrition, Metabolism and Cardiovascular Diseases*.

[127] Mangge H., Becker K., Fuchs D., Gostner J. M. (2014). Antioxidants, inflammation and cardiovascular disease. *World Journal of Cardiology*.

[128] Pacurari M., Kafoury R., Tchounwou P. B., Ndebele K. (2014). The renin-angiotensin-aldosterone system in vascular inflammation and remodeling. *International Journal of Inflammation*.

[129] Muñoz M. C., Burghi V., Miquet J. G. (2014). Downregulation of the ACE2/Ang-(1-7)/Mas axis in transgenic mice overexpressing GH. *Journal of Endocrinology*.

[130] Rachmin I., Tshori S., Smith Y. (2014). Erbin is a negative modulator of cardiac hypertrophy. *Proceedings of the National Academy of Sciences of the United States of America*.

[131] Takahashi T., Kubo H. (2014). The role of microparticles in chronic obstructive pulmonary disease. *International Journal of Chronic Obstructive Pulmonary Disease*.

[132] Hammerova L., Chabada J., Drobny J., Batorova A. (2014). Longitudinal evaluation of markers of hemostasis in pregnancy. *Bratislava Medical Journal*.

[133] Collins H. E., He L., Zou L. (2014). Stromal interaction molecule 1 is essential for normal cardiac homeostasis through modulation of ER and mitochondrial function. *American Journal of Physiology—Heart and Circulatory Physiology*.

[134] Xanthakis V., Larson M. G., Wollert K. C. (2013). Association of novel biomarkers of cardiovascular stress with left ventricular hypertrophy and dysfunction: implications for screening. *Journal of the American Heart Association*.

[135] Anderson R. H., Brown N. A., Mohun T. J., Moorman A. F. (2013). Insights from cardiac development relevant to congenital defects and adult clinical anatomy. *Journal of Cardiovascular Translational Research*.

[136] Beiras-Fernandez A., Weis F., Rothkopf J. (2013). Local expression of myocardial galectin-3 does not correlate with its serum levels in patients undergoing heart transplantation. *Annals of Transplantation*.

[137] de la Fuente R. L., Naesgaard P. A., Nilsen S. T. (2013). Omega-3 index and prognosis in acute coronary chest pain patients with a low dietary intake of omega-3. *Scandinavian Cardiovascular Journal*.

[138] Thifault E., Cormier H., Bouchard-Mercier A. (2013). Effects of age, sex, body mass index and APOE genotype on cardiovascular biomarker response to an n-3 polyunsaturated fatty acid supplementation. *Journal of Nutrigenetics and Nutrigenomics*.

[139] Fretts A. M., Mozaffarian D., Siscovick D. S. (2013). Associations of plasma phospholipid and dietary alpha linolenic acid with incident atrial fibrillation in older adults: the Cardiovascular Health Study. *Journal of the American Heart Association*.

[140] Nicholson T., Khademi H., Moghadasian M. H. (2013). The role of marine n-3 fatty acids in improving cardiovascular health: a review. *Food & Function*.

[141] Nilsson A., Radeborg K., Salo I., Björck I. (2012). Effects of supplementation with n-3 polyunsaturated fatty acids on cognitive performance and cardiometabolic risk markers in healthy 51 to 72 years old subjects: a randomized controlled cross-over study. *Nutrition Journal*.

[142] Baghai T. C., Varallo-Bedarida G., Born C. (2011). Major depressive disorder is associated with cardiovascular risk factors and low omega-3 index. *Journal of Clinical Psychiatry*.

[143] Ebrahimi M., Ghayour-Mobarhan M., Rezaiean S. (2009). Omega-3 fatty acid supplements improve the cardiovascular risk profile of subjects with metabolic syndrome, including markers of inflammation and auto-immunity. *Acta cardiologica*.

[144] Wain L. V. (2014). Rare variants and cardiovascular disease. *Briefings in Functional Genomics*.

[145] Welty F. K. (2014). Hypobetalipoproteinemia and abetalipoproteinemia. *Current Opinion in Lipidology*.

[146] Rice L. M., Donigan M., Yang M. (2014). Protein phosphatase 2A (PP2A) regulates low density lipoprotein uptake through regulating sterol response element-binding protein-2 (SREBP-2) DNA binding. *The Journal of Biological Chemistry*.

[147] Berkinbayev S., Rysuly M., Mussayev A. (2014). Apolipoprotein gene polymorphisms (APOB, APOC111, APOE) in the development of coronary heart disease in ethnic groups of Kazakhstan. *Journal of Genetic Syndromes & Gene Therapy*.

[148] Vučinić N., Djan I., Stokić E. (2014). Different associations of apoE gene polymorphism with metabolic syndrome in the Vojvodina Province (Serbia). *Molecular Biology Reports*.

[149] Sherrill J. D., Kiran K. C., Blanchard C. (2014). Analysis and expansion of the eosinophilic esophagitis transcriptome by RNA sequencing. *Genes and Immunity*.

[150] Schiano C., Casamassimi A., Vietri M. T., Rienzo M., Napoli C. (2014). The roles of mediator complex in cardiovascular diseases. *Biochimica et Biophysica Acta—Gene Regulatory Mechanisms*.

[151] Farhan S. M. K., Hegele R. A. (2014). Exome sequencing: new insights into lipoprotein disorders. *Current Cardiology Reports*.

[152] Suhre K. (2014). Metabolic profiling in diabetes. *Journal of Endocrinology*.

[153] Kuivenhoven J. A., Hegele R. A. (2014). Mining the genome for lipid genes. *Biochimica et Biophysica Acta—Molecular Basis of Disease*.

[154] Liu X., Lu L., Yao P. (2014). Lipopolysaccharide binding protein, obesity status and incidence of metabolic syndrome: a prospective study among middle-aged and older Chinese. *Diabetologia*.

[155] Nair N., Gupta S., Collier I. X., Gongora E., Vijayaraghavan K. (2014). Can microRNAs emerge as biomarkers in distinguishing HFpEF versus HFrEF?. *International Journal of Cardiology*.

[156] Scott P. A., Townsend P. A., Ng L. L. (2011). Defining potential to benefit from implantable cardioverter defibrillator therapy: the role of biomarkers. *Europace*.

[157] Cebe T., Atukeren P., Yanar K. (2014). Oxidation scrutiny in persuaded aging and chronological aging at systemic redox homeostasis level. *Experimental Gerontology*.

[158] Kawago M., Yoshimasu T., Tabata Y. (2014). Intrapleural administration of gelatin-embedded, sustained-release basic fibroblast growth factor for the regeneration of emphysematous lungs in rats. *The Journal of Thoracic and Cardiovascular Surgery*.

[159] Tsimaris P., Deligeoroglou E., Athanasopoulos N. (2014). The effect of hormone therapy on biochemical and ultrasound parameters associated with atherosclerosis in 46,XY DSD individuals with female phenotype. *Gynecological Endocrinology*.

[160] Sato H., Taketomi Y., Ushida A. (2014). The adipocyte-inducible secreted phospholipases PLA2G5 and PLA2G2E play distinct roles in obesity. *Cell Metabolism*.

[161] van Veen S., Sørensen D. M., Holemans T., Holen H. W., Palmgren M. G., Vangheluwe P. (2014). Cellular function and pathological role of ATP13A2 and related P-type transport ATPases in Parkinson's disease and other neurological disorders. *Frontiers in Molecular Neuroscience*.

[162] Chowdhury R., Warnakula S., Kunutsor S. (2014). Association of dietary, circulating, and supplement fatty acids with coronary risk: a systematic review and meta-analysis. *Annals of Internal Medicine*.

[163] Singhal A., Lanigan J., Storry C. (2013). Docosahexaenoic acid supplementation, vascular function and risk factors for cardiovascular disease: a randomized controlled trial in young adults. *Journal of the American Heart Association*.

[164] von Schacky C. (2011). The Omega-3 index as a risk factor for cardiovascular diseases. *Prostaglandins and Other Lipid Mediators*.

[165] Mitu F., Cobzaru R., Leon M. M. (2013). Influence of metabolic syndrome profile on cardiovascular risk. *Revista Medico-Chirurgicală a Societăţii de Medici şi Naturalişti din Iaşi*.

[166] de Vries M., Klop B., Castro Cabezas M. (2014). The use of the non-fasting lipid profile for lipid-lowering therapy in clinical practice—point of view. *Atherosclerosis*.

[167] Li L., Han J., Wang Z. (2014). Mass spectrometry methodology in lipid analysis. *International Journal of Molecular Sciences*.

[168] Sessa R., Pietro M. D., Filardo S., Turriziani O. (2014). Infectious burden and atherosclerosis: a clinical issue. *World Journal of Clinical Cases*.

[169] Rangel-Castilla L., Kalani M. Y. S., Russin J. (2014). 108 Cerebral revascularization in the endovascular era: clinical indications, surgical results and outcomes at the barrow neurological institute. *Neurosurgery*.

[170] Katsiki N., Mikhailidis D. P., Wierzbicki A. S. (2013). Epicardial fat and vascular risk: a narrative review. *Current Opinion in Cardiology*.

[171] Weitz J. I. (2014). Insights into the role of thrombin in the pathogenesis of recurrent ischaemia after acute coronary syndrome. *Thrombosis and Haemostasis*.

[172] Naumov V., Gorokhova S., Atkov O. (2014). P324Circadian genes in the regulation of lipids in coronary artery disease. *Cardiovascular Research*.

[173] Golia E., Limongelli G., Natale F. (2014). Inflammation and cardiovascular disease: from pathogenesis to therapeutic target. *Current Atherosclerosis Reports*.

[174] McMahon M., Skaggs B. (2014). Pathogenesis and treatment of atherosclerosis in lupus. *Rheumatic Disease Clinics of North America*.

[175] Martinic-Popovic I., Simundic A. M., Dukic L. (2014). The association of inflammatory markers with cerebral vasoreactivity and carotid atherosclerosis in transient ischaemic attack. *Clinical Biochemistry*.

[176] Yu W., Zha W., Guo S., Cheng H., Wu J., Liu C. (2014). Flos puerariae extract prevents myocardial apoptosis via attenuation oxidative stress in streptozotocin-induced diabetic mice. *PLoS ONE*.

[177] Arnold S. V., Kosiborod M., Tang F. (2014). Changes in low-density lipoprotein cholesterol levels after discharge for acute myocardial infarction in a real-world patient population. *American Journal of Epidemiology*.

[178] Kanmanthareddy A., Vallakati A., Sridhar A. (2014). The impact of atrial fibrillation and its treatment on dementia. *Current Cardiology Reports*.

[179] Kostapanos M. S., Elisaf M. S. (2014). High density lipoproteins and type 2 diabetes: emerging concepts in their relationship. *World Journal of Experimental Medicine*.

[180] Santos-Gallego C. G., Rosenson R. S. (2014). Role of HDL in those with diabetes. *Current Cardiology Reports*.

[181] Le Goff W., Guerin M., Chapman M. J. (2004). Pharmacological modulation of cholesteryl ester transfer protein, a new therapeutic target in atherogenic dyslipidemia. *Pharmacology & Therapeutics*.

[182] Jiang H., Yan W.-H., Li C.-J., Wang A.-P., Dou J.-T., Mu Y.-M. (2014). Elevated white blood cell count is associated with higher risk of glucose metabolism disorders in middle-aged and elderly Chinese people. *International Journal of Environmental Research and Public Health*.

[183] Nikus K., Birnbaum Y., Eskola M., Sclarovsky S., Zhong-Qun Z., Pahlm O. (2014). Updated electrocardiographic classification of acute coronary syndromes. *Current Cardiology Reviews*.

[184] Mozaffarian D., Shi P., Morris J. S. (2012). Mercury exposure and risk of hypertension in US men and women in 2 prospective cohorts. *Hypertension*.

[185] Yayan J. (2013). Emerging families of biomarkers for coronary artery disease: inflammatory mediators. *Vascular Health and Risk Management*.

[186] Chan D. C., Barrett P. H. R., Watts G. F. (2014). The metabolic and pharmacologic bases for treating atherogenic dyslipidaemia. *Best Practice & Research Clinical Endocrinology & Metabolism*.

[187] Cho S. W., Kim B. K., Kim J. H. (2013). Non-invasively measured aortic wave reflection and pulse pressure amplification are related to the severity of coronary artery disease. *Journal of Cardiology*.

[188] Kloos W., Vogel B., Blessing E. (2014). MiRNAs in peripheral artery disease—something gripping this way comes. *Vasa*.

[189] Truong Q. A., Januzzi J. L., Szymonifka J. (2014). Coronary sinus biomarker sampling compared to peripheral venous blood for predicting outcomes in patients with severe heart failure undergoing cardiac resynchronization therapy: the BIOCRT study. *Heart Rhythm*.

[190] Joosten M. M., Pai J. K., Bertoia M. L. (2014). *β*2-microglobulin, cystatin C, and creatinine and risk of symptomatic peripheral artery disease. *Journal of the American Heart Association*.

[191] Berezin A., Kremzer A. (2014). P194Serum uric acid as independent predictor of decreased number of circulating proangiogenic progenitor cells in asymptomatic coronary artery disease patients. *Cardiovascular Research*.

[192] Dumitriu I. E., Baruah P., Kaski J. (2014). P733Regulatory B cells from patients with coronary artery disease display numerical and functional alterations: a novel immune defect in atherosclerosis. *Cardiovascular Research*.

[193] Ryu S. K., Mallat Z., Benessiano J. (2012). Phospholipase A_2_ enzymes, high-dose atorvastatin, and prediction of ischemic events after acute coronary syndromes. *Circulation*.

[194] Kwaan H. C. (2014). From fibrinolysis to the plasminogen-plasmin system and beyond: a remarkable growth of knowledge, with personal observations on the history of fibrinolysis. *Seminars in Thrombosis and Hemostasis*.

[195] Velagapudi P., Turagam M. K., Leal M. A., Kocheril A. G. (2013). Atrial fibrosis: a risk stratifier for atrial fibrillation. *Expert Review of Cardiovascular Therapy*.

[196] Bryk D., Olejarz W., Zapolska-Downar D. (2014). Mitogen-activated protein kinases in atherosclerosis. *Postępy Higieny i Medycyny Doświadczalnej*.

[197] Luo J. Y., Ma Y. T., Xie X. (2014). Association of intercellular adhesion molecule1 gene polymorphism with coronary heart disease. *Molecular Medicine Reports*.

[198] McCall D. O., McKinley M. C., Noad R. (2011). The assessment of vascular function during dietary intervention trials in human subjects. *British Journal of Nutrition*.

[199] Bhattacharyya S., Hayward C., Pepper J., Senior R. (2012). Risk stratification in asymptomatic severe aortic stenosis: a critical appraisal. *European Heart Journal*.

[200] Chowdhury R., Warnakula S., Kunutsor S. (2014). Association of dietary, circulating, and supplement fatty acids with coronary risk: a systematic review and meta-analysis. *Annals of Internal Medicine*.

[201] Shah S. N., Arneja J. (2013). Efficacy of rosuvastatin in achieving target HDL, LDL, triglycerides and total cholesterol levels in type 2 diabetes mellitus (T2DM) with newly diagnosed dyslipidaemia: an open label, nonrandomised, non-interventional and observational study in India. *Journal of Association of Physicians of India*.

[202] Kato H., Nakanishi T., Arai H., Nishida H. I., Nishida T. (1989). Purification, microheterogeneity, and stability of human lipid transfer protein. *The Journal of Biological Chemistry*.

[203] Soulages J. L., Wells M. A. (1994). Lipophorin: the structure of an insect lipoprotein and its role in lipid transport in insects. *Advances in Protein Chemistry*.

[204] Oram J. F. (1983). Effects of high density lipoprotein subfractions on cholesterol homeostasis in human fibroblasts and arterial smooth muscle cells. *Arteriosclerosis*.

[205] Ockner R. K., Manning J. A. (1974). Fatty acid binding protein in small intestine. Identification, isolation, and evidence for its role in cellular fatty acid transport. *The Journal of Clinical Investigation*.

[206] Ockner R. K., Manning J. A., Kane J. P. (1982). Fatty acid binding protein. Isolation from rat liver, characterization, and immunochemical quantification. *The Journal of Biological Chemistry*.

[207] Veerkamp J. H., Peeters R. A., Maatman R. G. H. J. (1991). Structural and functional features of different types of cytoplasmic fatty acid-binding proteins. *Biochimica et Biophysica Acta*.

[208] Athanikar J. N., Osborne T. F. (1998). Specificity in cholesterol regulation of gene expression by coevolution of sterol regulatory DNA element and its binding protein. *Proceedings of the National Academy of Sciences of the United States of America*.

[209] Smith A. F., Tsuchida K., Hanneman E., Suzuki T. C., Wells M. A. (1992). Isolation, characterization, and cDNA sequence of two fatty acid-binding proteins from the midgut of Manduca sexta larvae. *The Journal of Biological Chemistry*.

[210] van Heusden M. C., van der Horst D. J., van Doorn J. M., Beenakkers A. M. T. (1987). Partial purification of locust flight muscle lipoprotein lipase (LpL): apparent differences from mammalian LpL. *Comparative Biochemistry and Physiology—Part B: Biochemistry and*.

[211] Stahl A., Hirsch D. J., Gimeno R. E. (1999). Identification of the major intestinal fatty acid transport protein. *Molecular Cell*.

[212] Nemecz G., Schroeder F. (1991). Selective binding of cholesterol by recombinant fatty acid binding proteins. *The Journal of Biological Chemistry*.

[213] Richeri G. V., Ogata R. T., Kleinfield A. M. (1994). Equilibrium constants for the binding of fatty acids with fatty acid-binding proteins from adipocyte, intestine, heart, and liver measured with the fluorescent probe ADIFAB. *The Journal of Biological Chemistry*.

[214] Haunerland N. H., Chisholm J. M. (1990). Fatty acid binding protein in flight muscle of the locus, *Schistocerca gregaria*. *Biochimica et Biophysica Acta*.

[215] Ayala-Sanmartin J. (2001). Cholesterol enhances phospholipid binding and aggregation of annexins by their core domain. *Biochemical and Biophysical Research Communications*.

[216] Pentchev P. G., Brady R. O., Blanchette-Mackie E. J. (1994). The Niemann-Pick C lesion and its relationship to the intracellular distribution and utilization of LDL cholesterol. *Biochimica et Biophysica Acta*.

[217] Xu X.-X., Tabas I. (1991). Lipoproteins activate acyl-coenzyme A: cholesterol acyltransferase in macrophages only after cellular cholesterol pools are expanded to a critical threshold level. *Journal of Biological Chemistry*.

[218] Olofsson S.-O., Asp L., Borén J. (1999). The assembly and secretion of apolipoprotein B-containing lipoproteins. *Current Opinion in Lipidology*.

[219] Chang T. Y., Chang C. C. Y., Cheng D. (1997). Acyl-coenzyme A: cholesterol acyltransferase. *Annual Review of Biochemistry*.

[220] Chappell D. A., Fry G. L., Waknitz M. A., Berns J. J. (1991). Ligand size as a determinant for catabolism by the low density lipoprotein (LDL) receptor pathway. *The Journal of Biological Chemistry*.

[221] Lange Y., Ye J., Rigney M., Steck T. (2000). Cholesterol movement in Niemann-Pick type C cells and in cells treated with amphiphiles. *Journal of Biological Chemistry*.

[222] Merino J., Masana L., Guijarro C., Ascaso J., Lagares M., Civeira F. (2014). Recomendations for clinical use of food enriched phytosterols/phytostanols handling hypercholesterolemia. *Clínica e Investigación en Arteriosclerosis*.

[223] Greyshock N., Guyton J. R., Sebastian S., Okorodudu D., Pagon R. A., Adam M. P., Ardinger H. H. (1993–2014). APOE p.Leu167del-related lipid disorders. *GeneReviews*.

[224] Hoogeveen E. K., Geleijnse J. M., Kromhout D. (2014). No effect of n-3 fatty acids supplementation on NT-proBNP after myocardial infarction: The Alpha Omega Trial. *European Journal of Preventive Cardiology*.

[225] Strickland D. K., Au D. T., Cunfer P., Muratoglu S. C. (2014). Low-density lipoprotein receptor-related protein-1: role in the regulation of vascular integrity. *Arteriosclerosis, Thrombosis, and Vascular Biology*.

[226] Hirayama S., Miida T. (2012). Small dense LDL: an emerging risk factor for cardiovascular disease. *Clinica Chimica Acta*.

[227] Xu X.-X., Tabas I. (1991). Lipoproteins activate acyl-coenzyme A:cholesterol acyl transferase in macrophages only after cellular cholesterol pools are expanded to a critical threshold level. *Journal of Biological Chemistry*.

[228] Ikonen E. (2008). Cellular cholesterol trafficking and compartmentalization. *Nature Reviews Molecular Cell Biology*.

[229] Clarke S. D., Armstrong M. K. (1989). Cellular lipid binding proteins: expression, function, and nutritional regulation. *The FASEB Journal*.

[230] Kosmas C. E., Christodoulidis G., Cheng J.-W., Vittorio T. J., Lerakis S. (2014). High-density lipoprotein functionality in coronary artery disease. *The American Journal of the Medical Sciences*.

[231] Sniderman A., Kwiterovich P. O. (2013). Update on the detection and treatment of atherogenic low-density lipoproteins. *Current Opinion in Endocrinology, Diabetes and Obesity*.

[232] Ramjee V., Sperling L. S., Jacobson T. A. (2011). Non-high-density lipoprotein cholesterol versus apolipoprotein B in cardiovascular risk stratification: do the math. *Journal of the American College of Cardiology*.

[233] de Vries M., Klop B., Cabezas M. C. (2014). The use of the non-fasting lipid profile for lipid-lowering therapy in clinical practice—point of view. *Atherosclerosis*.

[234] Koldamova R., Fitz N. F., Lefterov I. (2014). ATP-binding cassette transporter A1: from metabolism to neurodegeneration. *Neurobiology of Disease*.

[235] Stechschulte L. A., Hinds T. D., Khuder S. S., Shou W., Najjar S. M., Sanchez E. R. (2014). FKBP51 controls cellular adipogenesis through p38 kinase-mediated phosphorylation of GR*α* and PPAR*γ*. *Molecular Endocrinology*.

[236] Wu C.-L., Zhao S.-P., Yu B.-L. (2014). Intracellular role of exchangeable apolipoproteins in energy homeostasis, obesity and non-alcoholic fatty liver disease. *Biological reviews of the Cambridge Philosophical Society*.

[237] Kwong M., Wasan K. M. (2002). Cholesteryl ester transfer protein facilitates the movement of water-insoluble drugs between lipoproteins: a novel biological function for a well-characterized lipid transfer protein. *Biochemical Pharmacology*.

[238] Rao R., Albers J. J., Wolfbauer G., Pownall H. J. (1997). Molecular and macromolecular specificity of human plasma phospholipid transfer protein. *Biochemistry*.

[239] Ohnishi T., Oikawa K., Kay C. M., Yokoyama S. (1995). Modulation of substrate selectivity in plasma lipid transfer protein reaction over structural variation of lipid particle. *Biochimica et Biophysica Acta*.

[240] Ohnishi T., Tan C., Yokoyama S. (1994). Selective transfer of cholesteryl ester over triglyceride by human plasma lipid transfer protein between apolipoprotein-activated lipid microemulsions. *Biochemistry*.

[241] Nishida H. I., Arai H., Nishida T. (1993). Cholesterol ester transfer mediated by lipid transfer protein as influenced by changes in the charge characteristics of plasma lipoproteins. *The Journal of Biological Chemistry*.

[242] Ohnishi T., Yokoyama S. (1993). Activation of human plasma lipid transfer protein by apolipoproteins. *Biochemistry*.

[243] Ryan R. O., Yokoyama S., Liu H., Czarnecka H., Oikawa K., Kay C. M. (1992). Human apolipoprotein A-I liberated from high-density lipoprotein without denaturation. *Biochemistry*.

[244] Ohnishi T., Oikawa K., Kay C. M., Yokoyama S. (1995). Modulation of substrate selectivity in plasma lipid transfer protein reaction over structural variation of lipid particle. *Biochimica et Biophysica Acta—Lipids and Lipid Metabolism*.

[245] Silver E. T., Scraba D. G., Ryan R. O. (1990). Lipid transfer particle-induced transformation of human high density lipoprotein into apolipoprotein A-I-deficient low density particles. *The Journal of Biological Chemistry*.

[246] Morton R. E. (1990). Interaction of lipid transfer protein with plasma lipoproteins and cell membranes. *Experientia*.

[247] Morton R. E., Steinbrunner J. V. (1990). Concentration of neutral lipids in the phospholipid surface of substrate particles determines lipid transfer protein activity. *Journal of Lipid Research*.

[248] Groener J. E. M., van Gent T., van Tol A. (1989). Effect of lipid transfer protein on plasma lipids, apolipoproteins and metabolism of high-density lipoprotein cholesteryl ester in the rat. *Biochimica et Biophysica Acta*.

[249] Nishide T., Tollefson J. H., Albers J. J. (1989). Inhibition of lipid transfer by a unique high density lipoprotein subclass containing an inhibitor protein. *Journal of Lipid Research*.

[250] Morton R. E. (1988). Free cholesterol is a potent regulatore of lipid transfer protein function. *The Journal of Biological Chemistry*.

[251] Abbey M., Bastiras S., Calvert G. D. (1985). Immunoprecipitation of lipid transfer protein activity by an antibody against human plasma lipid transfer protein-I. *Biochimica et Biophysica Acta—Lipids and Lipid Metabolism*.

[252] Sviridov D. D., Misharin A. Y., Safonova I. G., Bushmakina N. G., Repin V. S., Smirnov V. N. (1988). Binding of partially reassembled high-density lipoprotein to isolated human small intestine epithelial cells. Effect of lipid composition. *Biochimica et Biophysica Acta*.

[253] van der Horst D. J., Stoppie P., Huybrechts R., de Loof A., Beenakkers A. M. T. (1981). Immunological relationships between the diacylglycerol-transporting lipoproteins in the haemolymph of Locusta. *Comparative Biochemistry and Physiology*.

[254] van der Horst D. J., van Doorn J. M., de Keijzer A. N., Beenakkers A. M. T. (1981). Interconversions of diacylglycerol-transporting lipoproteins in the haemolymph of *Locusta migratoria*. *Insect Biochemistry*.

[255] Wells M. A., Ryan R. O., Kawooya J. K., Law J. H. (1987). The role of apolipophorin III in in vivo lipoprotein interconversions in adult *Manduca sexta*. *The Journal of Biological Chemistry*.

[256] Singh T. K. A., Ryan R. O. (1991). Lipid transfer particle-catalyzed transfer of lipoprotein-associated diacylglycerol and long chain aliphatic hydrocarbons. *Archives of Biochemistry and Biophysics*.

[257] Sukhorukova E. G., Bekoeva S. A., Korzhevskaia V. F., Tsukanova A. F., Korzhevskiǐ D. É. (2013). Diagnostic potential of the histochemical methods used in histological studies of the heart. *Sudebno-Meditsinskaia Ekspertiza*.

[258] Mayer R. M., Treadwell C. R., Gallo L. L., Vahouny G. V. (1985). Intestinal mucins and cholesterol uptake *in vitro*. *Biochimica et Biophysica Acta*.

[259] Haruo C., Downer R. G. H., Koui T. (1977). The role of diacylglycerol-carrying lipoprotein I in lipid transport during insect vitellogenesis. *Biochimica et Biophysica Acta—Lipids and Lipid Metabolism*.

[260] Klucken J., Büchler C., Orsó E. (2000). ABCG1 (ABC8), the human homolog of the *Drosophila* white gene, is a regulator of macrophage cholesterol and phospholipid transport. *Proceedings of the National Academy of Sciences of the United States of America*.

[261] Upadhyay R. K., Agarwal H. C., Dhar R. (2002). Protein mediated cholesterol absorption in locusts *Schistocerca gregaria* (Forskal) and *Locusta migratoria* (Linn). *Indian Journal of Experimental Biology*.

[262] Yun H. K., Jouni Z. E., Wells M. A. (2002). Characterization of cholesterol transport from midgut to fat body in *Manduca sexta* larvae. *Insect Biochemistry and Molecular Biology*.

[263] Vahouny G. V., Chanderbhan R., Stewart P. (1985). Phospholipids, sterol carrier protein _2_ and adrenal steroidogenesis. *Biochimica et Biophysica Acta*.

[264] Tam S.-P., Mok L., Chimini G., Vasa M., Deeley R. G. (2006). ABCA1 mediates high-affinity uptake of 25-hydroxycholesterol by membrane vesicles and rapid efflux of oxysterol by intact cells. *The American Journal of Physiology—Cell Physiology*.

[265] Radhakrishnan A., Sun L.-P., Kwon H. J., Brown M. S., Goldstein J. L. (2004). Direct binding of cholesterol to the purified membrane region of SCAP: mechanism for a sterol-sensing domain. *Molecular Cell*.

[266] Wang J., Sun F., Zhang D.-W. (2006). Sterol transfer by ABCG5 and ABCG8: *in vitro* assay and reconstitution. *The Journal of Biological Chemistry*.

[267] Bass N. M. (1988). The cellular fatty acid binding proteins: aspects of structure, regulation, and function. *International Review of Cytology*.

[268] Brown M. S., Goldstein J. L. (1986). A receptor-mediated pathway for cholesterol homeostasis. *Science*.

[269] Mitsos S., Koletsis E. N., Katsanos K. (2014). Intramyocardial thrombin promotes angiogenesis and improves cardiac function in an experimental rabbit model of acute myocardial infarction. *Journal of Thoracic and Cardiovascular Surgery*.

[270] Steyers F. J., Miller C. M. (2014). Endothelial dysfunction in chronic inflammatory diseases. *International Journal of Molecular Sciences*.

[271] Zheng T. P., Yang F., Gao Y. (2014). Increased plasma DPP4 activities predict new-onset atherosclerosis in association with its proinflammatory effects in Chinese over a four year period: a prospective study. *Atherosclerosis*.

[272] Yin K., Agrawal D. K. (2014). Vitamin D and inflammatory diseases. *Journal of Inflammation Research*.

[273] Joshi Y. B., Praticò D. (2014). Lipid peroxidation in psychiatric illness: overview of clinical evidence. *Oxidative Medicine and Cellular Longevity*.

[274] Schnabel R. B., Seiffert M., Wilde S. (2015). Kidney injury and mortality after transcatheter aortic valve implantation in a routine clinical cohort. *Catheterization and Cardiovascular Interventions*.

[275] Grassia G., MacRitchie N., Platt A. M., Brewer J. M., Garside P., Maffia P. (2013). Plasmacytoid dendritic cells: biomarkers or potential therapeutic targets in atherosclerosis?. *Pharmacology & Therapeutics*.

[276] Lappegård K. T., Garred P., Jonasson L. (2014). A vital role for complement in heart disease. *Molecular Immunology*.

[277] Fullerton J. N., O'Brien A. J., Gilroy D. W. (2013). Pathways mediating resolution of inflammation: when enough is too much. *Journal of Pathology*.

[278] Weckbach L. T., Gola A., Winkelmann M. (2014). The cytokine midkine supports neutrophil trafficking during acute inflammation by promoting adhesion via *β*2 integrins (CD11/CD18). *Blood*.

[279] Martynowicz H., Janus A., Nowacki D., Mazur G. (2014). The role of chemokines in hypertension. *Advances in Clinical and Experimental Medicine*.

[280] Al-Ghoul W. M., Kim M. S., Fazal N., Azim A. C., Ali A. (2014). Evidence for simvastatin anti-inflammatory actions based on quantitative analyses of NETosis and other inflammation/oxidation markers. *Results in Immunology*.

[281] de Andrade K. R., de Castro G. R. W., Vicente G. (2014). Evaluation of circulating levels of inflammatory and bone formation markers in axial spondyloarthritis. *International Immunopharmacology*.

[282] Swede H., Hajduk A. M., Sharma J. (2014). Baseline serum C-reactive protein and death from colorectal cancer in the NHANES III cohort. *International Journal of Cancer*.

[283] Kaptoge S., di Angelantonio E., Pennells L., Wood A. M., White I. R., Gao P. (2012). C-reactive protein, fibrinogen, and cardiovascular disease prediction. *The New England Journal of Medicine*.

[284] Ferroni P., Riondino S., Vazzana N., Santoro N., Guadagni F., Davì G. (2012). Biomarkers of platelet activation in acute coronary syndromes. *Thrombosis and Haemostasis*.

[285] Jantsch J., Binger K. J., Müller D. N., Titze J. (2014). Macrophages in homeostatic immune function. *Frontiers in Physiology*.

[286] Voǐtsekhovskis V. V., Voǐtsekhovska I. G., Shkesters A. (2014). Advances of selenium supplementation in posttraumatic stress disorder risk group patients. *Biomeditsinskaia Khimiia*.

[287] Kreis P., Leondaritis G., Lieberam I., Eickholt B. J. (2014). Subcellular targeting and dynamic regulation of PTEN: implications for neuronal cells and neurological disorders. *Frontiers in Molecular Neuroscience*.

[288] Korman B. D., Huang C.-C., Skamra C. (2014). Inflammatory expression profiles in monocyte to macrophage differentiation in patients with systemic lupus erythematosus and relationship with atherosclerosis. *Arthritis Research & Therapy*.

[289] Chen L. Q., de Lemos J. A., Das S. R., Ayers C. R., Rohatgi A. (2013). Soluble ST2 is associated with all-cause and cardiovascular mortality in a population-based cohort: the dallas heart study. *Clinical Chemistry*.

[290] Shah K. B., Kop W. J., Christenson R. H. (2011). Prognostic utility of ST2 in patients with acute dyspnea and preserved left ventricular ejection fraction. *Clinical Chemistry*.

[291] Ky B., French B., McCloskey K. (2011). High-sensitivity ST2 for prediction of adverse outcomes in chronic heart failure. *Circulation: Heart Failure*.

[292] Felker G. M., Fiuzat M., Thompson V. (2013). Soluble ST2 in ambulatory patients with heart failure: association with functional capacity and long-term outcomes. *Circulation: Heart Failure*.

[293] Bayes-Genis A., de Antonio M., Vila J. (2014). Head-to-head comparison of 2 myocardial fibrosis biomarkers for long-term heart failure risk stratification: ST2 versus galectin-3. *Journal of the American College of Cardiology*.

[294] Dieplinger B., Egger M., Haltmayer M. (2014). Increased soluble ST2 predicts long-term mortality in patients with stable coronary artery disease: results from the Ludwigshafen risk and cardiovascular health study. *Clinical Chemistry*.

[295] Kohli P., Bonaca M. P., Kakkar R. (2012). Role of ST2 in non-ST-elevation acute coronary syndrome in the MERLIN-TIMI 36 trial. *Clinical Chemistry*.

[296] Wang T. J., Wollert K. C., Larson M. G. (2012). Prognostic utility of novel biomarkers of cardiovascular stress: the framingham heart study. *Circulation*.

[297] Fitchett D. H., Mancini G. B. J., Gregoire J., Anderson T., McPherson R. (2014). Risk stratification and selection for statin therapy: going beyond framingham. *Canadian Journal of Cardiology*.

[298] Habibi M., Chahal H., Opdahl A. (2014). Association of CMR-measured LA function with heart failure development: results from the MESA Study. *JACC: Cardiovascular Imaging*.

[299] Panovsky R., Pleva M., Feitova V., Kruzliak P., Meluzin J., Kincl V. (2014). The prognostic impact of myocardial late gadolinium enhancement. *Cardiology in Review*.

[300] Ferrario C. M., Strawn W. B. (2006). Role of the renin-angiotensin-aldosterone system and proinflammatory mediators in cardiovascular disease. *The American Journal of Cardiology*.

[301] Raut S., Hamill M., Daniels S., Heath A. B., The Subcommittee on Factor XIII/Fibrinogen (2014). Value assignment to the WHO 3rd International Standard for Blood Coagulation Fibrinogen Plasma (09/264): communication from the SSC of the ISTH. *Journal of Thrombosis and Haemostasis*.

[302] Raut S., Hamill M., Daniels S., Heath A. B. (2014). Value assignment to the WHO 3rd International Standard for Blood Coagulation Fibrinogen Plasma (09/264): communication from the SSC of the ISTH. *Journal of Thrombosis and Haemostasis*.

[303] Zsíros N., Paragh G., Harangi M. (2014). Clinical significance and treatment options of increased lipoprotein(a). *Orvosi Hetilap*.

[304] Santoro D., Gagliostro G., Alibrandi A. (2014). Vitamin D receptor gene polymorphism and left ventricular hypertrophy in chronic kidney disease. *Nutrients*.

[305] Tornhammar P., Ueda P., Hult M., Simila H., Eyles D., Norman M. (2014). Season of birth, neonatal vitamin D status, and cardiovascular disease risk at 35 y of age: a cohort study from Sweden. *American Journal of Clinical Nutrition*.

[306] Hirai D. M., Copp S. W., Holdsworth C. T. (2014). Skeletal muscle microvascular oxygenation dynamics in heart failure: exercise training and nitric oxide-mediated function. *The American Journal of Physiology—Heart and Circulatory Physiology*.

[307] Matsuoka K., Asano Y., Higo S. (2014). Noninvasive and quantitative live imaging reveals a potential stress-responsive enhancer in the failing heart. *The FASEB Journal*.

[308] Hurford R., Charidimou A., Fox Z., Cipolotti L., Jager R., Werring D. J. (2014). MRI-visible perivascular spaces: relationship to cognition and small vessel disease MRI markers in ischaemic stroke and TIA. *Journal of Neurology, Neurosurgery and Psychiatry*.

[309] Hurford R., Charidimou A., Fox Z., Cipolotti L., Jager R., Werring D. J. (2014). MRI-visible perivascular spaces: Relationship to cognition and small vessel disease MRI markers in ischaemic stroke and TIA. *Journal of Neurology, Neurosurgery and Psychiatry*.

[310] Clària J., Nguyen B. T., Madenci A. L., Ozaki C. K., Serhan C. N. (2013). Diversity of lipid mediators in human adipose tissue depots. *The American Journal of Physiology—Cell Physiology*.

[311] Moylan S., Berk M., Dean O. M. (2014). Oxidative & nitrosative stress in depression: why so much stress?. *Neuroscience and Biobehavioral Reviews*.

[312] Lipina C., Irving A. J., Hundal H. S. (2014). Mitochondria: a possible nexus for the regulation of energy homeostasis by the endocannabinoid system. *American Physiological Society—Endocrinology and Metabolism*.

[313] Dawczynski C., Massey K. A., Ness C. (2013). Randomized placebo-controlled intervention with n-3 LC-PUFA-supplemented yoghurt: effects on circulating eicosanoids and cardiovascular risk factors. *Clinical Nutrition*.

[314] Wu H., Ding E. L., Toledo E. T. (2013). A novel fatty acid lipophilic index and risk of CHD in US men: the Health Professionals Follow-Up Study. *British Journal of Nutrition*.

[315] Salisbury A. C., Amin A. P., Harris W. S. (2011). Predictors of omega-3 index in patients with acute myocardial infarction. *Mayo Clinic Proceedings*.

[316] Sala-Vila A., Harris W. S., Cofán M. (2011). Determinants of the omega-3 index in a Mediterranean population at increased risk for CHD. *British Journal of Nutrition*.

[317] Harris W. S. (2009). The omega-3 index: from biomarker to risk marker to risk factor. *Current Atherosclerosis Reports*.

[318] Birlouez-Aragon I., Saavedra G., Tessier F. J. (2010). A diet based on high-heat-treated foods promotes risk factors for diabetes mellitus and cardiovascular diseases. *The American Journal of Clinical Nutrition*.

[319] Caspar-Bauguila S., Garcia J., Galiniera A. (2010). Positive impact of long-term lifestyle change on erythrocyte fatty acid profile after acute coronary syndromes. *Archives of Cardiovascular Diseases*.

[320] Ming G. F., Li X., Yin J. Y. (2014). JAZF1 regulates visfatin expression in adipocytes via PPAR*α* and PPAR*β*/*δ* signaling. *Metabolism*.

[321] Koutsis G., Siasos G., Spengos K. (2013). The emerging role of microRNA in stroke. *Current Topics in Medicinal Chemistry*.

[322] Mirbolouk M., Asgari S., Sheikholeslami F., Mirbolouk F., Azizi F., Hadaegh F. (2014). Different obesity phenotypes, and incident cardiovascular disease and mortality events in elderly iranians: Tehran lipid and glucose study. *Geriatrics and Gerontology International*.

[323] Mavrogeni S., Sfikakis P., Dimitroulas T., Kolovou G., Kitas G. (2014). Cardiac and muscular involvement in idiopathic inflammatory myopathies: noninvasive diagnostic assessment and the role of cardiovascular and skeletal magnetic resonance imaging. *Inflammation & Allergy-Drug Targets*.

[324] Abdurrachim D., Ciapaite J., Wessels B. (2014). Cardiac diastolic dysfunction in high-fat diet fed mice is associated with lipotoxicity without impairment of cardiac energetics in vivo. *Biochimica et Biophysica Acta*.

[325] Yorozu T. (2014). Prevention of venous thromboembolism and anticoagulant therapy. *Japanese Journal of Anesthesiology*.

[326] Mahmoudizad R., Samrao A., Bentow J. J., Peng S.-K., Bhatia N. (2014). Composite hemangioendothelioma: an unusual presentation of a rare vascular tumor. *American Journal of Clinical Pathology*.

[327] Romero J. R., Preis S. R., Beiser A. (2014). Risk factors, stroke prevention treatments, and prevalence of cerebral microbleeds in the framingham heart study. *Stroke*.

[328] Goel R., Kumar T. S., Danda D. (2014). Childhood-onset Takayasu arteritis—experience from a tertiary care center in south India. *Journal of Rheumatology*.

[329] Riaz I. B., Singh A., Janardhanan R. (2014). Chest pain and elevated troponins in a patient with prior coronary artery disease: a diagnostic dilemma. *The American Journal of Medicine*.

[330] Furtado M. B., Costa M. W., Pranoto E. A. (2014). Cardiogenic genes expressed in cardiac fibroblasts contribute to heart development and repair. *Circulation Research*.

[331] Yadav S. S., Singh M. K., Dwivedi P. (2014). Significance of impaired serum gelatinases activities in metabolic syndrome. *Toxicology International*.

[332] Tóth Š., Pekárová T., Varga J. (2013). Trehalase as a possible marker of intestinal ischemia—reperfusion injury. *Acta Biochimica Polonica*.

[333] Zhang Q., Xiao X., Li M. (2014). Berberine moderates glucose metabolism through the GnRH-GLP-1 and MAPK pathways in the intestine. *BMC Complementary and Alternative Medicine*.

[334] Bialasiewicz P., Prymont-Przyminska A., Zwolinska A. (2014). Addition of strawberries to the usual diet decreases resting chemiluminescence of fasting blood in healthy subjects-possible health-promoting effect of these fruits consumption. *Journal of the American College of Nutrition*.

[335] Gano L. B., Patel M., Rho J. M. (2014). Ketogenic diets, mitochondria, and neurological diseases. *The Journal of Lipid Research*.

[336] Parolini C., Vik R., Busnelli M. (2014). A salmon protein hydrolysate exerts lipid-independent anti-atherosclerotic activity in apoE-deficient mice. *PLoS ONE*.

[337] Vivek H. K., Swamy S. G., Priya B. S., Sethi G., Rangappa K. S., Swamy S. N. (2014). A facile assay to monitor secretory phospholipase A_2_ using 8-anilino-1-naphthalenesulfonic acid. *Analytical Biochemistry*.

[338] Shichiri M. (2014). The role of lipid peroxidation in neurological disorders. *Journal of Clinical Biochemistry and Nutrition*.

[339] Sahu B. D., Putcha U. K., Kuncha M., Rachamalla S. S., Sistla R. (2014). Carnosic acid promotes myocardial antioxidant response and prevents isoproterenol-induced myocardial oxidative stress and apoptosis in mice. *Molecular and Cellular Biochemistry*.

[340] Le O. T., Nguyen T. T., Lee S. Y. (2014). Phosphoinositide turnover in Toll-like receptor signaling and trafficking. *BMB Reports*.

[341] Keyamura Y., Nagano C., Kohashi M. (2014). Add-on effect of probucol in atherosclerotic, cholesterol-fed rabbits treated with atorvastatin. *PLoS ONE*.

[342] Herrick C., Litvin M., Goldberg A. C. (2014). Lipid lowering in liver and chronic kidney disease. *Best Practice and Research: Clinical Endocrinology and Metabolism*.

[343] Albert J. S., Yerges-Armstrong L. M., Horenstein R. B. (2014). Null mutation in hormone-sensitive lipase gene and risk of type 2 diabetes. *The New England Journal of Medicine*.

[344] Luongo E., Russo R., Avagliano C. (2014). Galactosyl prodrug of palmitoylethanolamide: Synthesis, stability, cell permeation and cytoprotective activity. *European Journal of Pharmaceutical Sciences*.

[345] Vinod R. K., Shashikala M., Suresh K. P. (2013). Distribution of certain risk factors among dyslipidaemic patients, morbid with first episode coronary heart disease. *Journal of the Indian Medical Association*.

[346] Page M. M., Bell D. A., Hooper A. J., Watts G. F., Burnett J. R. (2014). Lipoprotein apheresis and new therapies for severe familial hypercholesterolemia in adults and children. *Best Practice and Research: Clinical Endocrinology and Metabolism*.

[347] Chan D. C., Barrett P. H. R., Watts G. F. (2014). The metabolic and pharmacologic bases for treating atherogenic dyslipidaemia. *Best Practice and Research: Clinical Endocrinology and Metabolism*.

[348] Toth P. P., Shah P. K., Wilkinson M. J., Davidson M. H., McCullough P. A. (2014). Use of microsomal triglyceride transfer protein inhibitors in patients with homozygous familial hypercholesterolemia: translating clinical trial experience into clinical practice. *Reviews in Cardiovascular Medicine*.

[349] Koozehchian M. S., Nazem F., Kreider R. B. (2014). The role of exercise training on lipoprotein profiles in adolescent males. *Lipids in Health and Disease*.

[350] Hussain M. M. (2014). Intestinal lipid absorption and lipoprotein formation. *Current Opinion in Lipidology*.

[351] Kim J., Kim Y.-S., Lee H. A. (2014). *Sasa quelpaertensis* leaf extract improves high fat diet-induced lipid abnormalities and regulation of lipid metabolism genes in rats. *Journal of Medicinal Food*.

[352] Eggert M.-L., Wallaschofski H., Grotevendt A. (2014). Cross-sectional and longitudinal relation of IGF1 and IGF-binding protein 3 with lipid metabolism. *European Journal of Endocrinology*.

[353] Kwiatkowska K., Marszałek-Sadowska E., Traczyk G. (2014). Visualization of cholesterol deposits in lysosomes of Niemann-Pick type C fibroblasts using recombinant perfringolysin O. *Orphanet Journal of Rare Diseases*.

[354] De Vos L. C., Mulder D. J., Smit A. J. (2014). Skin autofluorescence is associated with 5-year mortality and cardiovascular events in patients with peripheral artery disease. *Arteriosclerosis, Thrombosis, and Vascular Biology*.

[355] Lim L., Banwell C., Bain C. (2014). Sugar sweetened beverages and weight gain over 4 years in a Thai national cohort—a prospective analysis. *PLoS ONE*.

